# Prediction of single cell expression from low-plex immunofluorescence images for guiding precision oncology

**DOI:** 10.1038/s41698-026-01601-z

**Published:** 2026-07-25

**Authors:** Markus Heidrich, Daniel Nilsson, Asger Meldgaard Frank, Mohammad Kazemi Majdabadi, Maria Louise Elkjær, Jan Baumbach, Karin Sundfeldt, Sara Ek, Anna Gerdtsson

**Affiliations:** 1 Department of Immunotechnology, Lund University, Lund, Sweden; 2 Institute for Computational Systems Biology, Hamburg University, Hamburg, Germany; 3 Department of Obstetrics and Gynecology, Sahlgrenska Academy at University of Gothenburg, Gothenburg, Sweden

**Keywords:** Biomarkers, Cancer, Computational biology and bioinformatics, Oncology

## Abstract

Spatial omics allows for comprehensive investigation of the tumor immune microenvironment (TIME). Stratifying patients by their TIMEs contributes with insights in tumor immune response and has the potential to guide treatment decision-making in the immuno-oncology setting. However, high-plex spatial omics approaches still suffer from high costs and limited direct clinical applicability. We address this issue by presenting a deep learning model, Image2Count, for deconvoluting molecular expression from low-plex immunofluorescence imaging. Explicitly, our model learns visual representations of cells in a contrastive manner, utilizing Graph Neural Networks to predict expressions from cell graphs, enabling the trained model to predict high-plex single-cell expression from just four marker images. We measure model performance using an ovarian cancer GeoMx dataset with “bulk” Region of Interest 72-plex protein counts, a Cyclic IF (t-CyCIF) 25-plex single-cell resolution colorectal cancer dataset, and a 960-plex RNA single-cell resolution CosMx non-small cell lung cancer dataset. Image2Count is able to predict distinct spatial expression patterns of subsets of tumor, immune and stromal cells, and displays a generally improved accuracy when considering neighborhoods of cells over single cells. Concordance of pathways enriched in true and predicted data indicates the ability to capture biologically relevant information. Our model paves the way for clinically implementable TIME stratification based on low-plex immunofluorescence images, and allows for standard single-cell analysis workflows to interpret multicellular expression data from regions of interest.

## Introduction

Tumor microenvironment complexity and heterogeneity hamper the selection of patients for immunotherapy. Hence, stratification of patients and identification of drug targets for individualized medicine, based on molecular profiling, is clinically relevant and an area of active research^1–3^. Recent studies have shown that the tumor immune microenvironment (TIME) can hold information that is prognostic for treatment outcome^4–6^. Regions of interests (ROIs) in the tissue can be analyzed using spatial omics approaches, through Hematoxylin and Eosin (H&E) or multiplex imaging combined with spot- or region-based transcriptomics and/or proteomics^4,6–9^. Several approaches have emerged to leverage spatial omics data, including identification of spatial niches^7,9–11^, correction of batch effects^10^, and imputation of missing count data^11^. Despite this progress, image data remain underutilized. The association between stained tissue regions and their expression data may reveal biological signals that are difficult to detect when data are analyzed in a disassociated fashion. While new technologies enable even sub-cellular localization of transcripts^8,12^, their use in clinical settings remains limited due to high cost, time consumption and complexity.

Several studies have addressed this issue by predicting expression data from images^13^, particularly by linking H&E staining to spot counts from the Visium platform^8^. Most workflows are based on cropping images into patches and encoding visual information for each patch, while others encode visual information for each cell in a cell graph^14^. Schmau et al. embedded H&E patches into a visual representation through a pre-trained ResNet50 and applied a multilayer Perceptron (MLP) to produce tile-level predictions, which were then aggregated in a weighted manner into slide-level predictions while minimizing the distance to corresponding slide RNA-seq data^15^. Monjo et al. trained a modified VGG16 architecture to predict Visium counts^8^ from corresponding H&E spots^16^. Fatemi et al. used a pre-trained InceptionV3 model to estimate zero-inflated negative binomial distributions of Visium spot gene expression from corresponding H&E slide patches by optimizing the negative log likelihood of the predicted distribution, and further compared these predictions to a Vision Transformer and Graph Attention Networks (GATs) trained with the same loss as the InceptionV3 model^17^. In related work, the same group jointly trained a ResNet50 for cell-level visual feature extraction from H&E images and a GAT predicting cell expression from a cell graph by minimizing the mean squared error (MSE) between Visium spot gene counts and predicted counts, and penalizing single-cell expression through optimal flow methods^14^. Additional approaches predicting Visium spot expression utilizing corresponding H&E images include Swin Transformers^18^, fine-tuning pre-trained DenseNet121 models^19^, the pathology foundation model UNI including multi-head self-attention^20^, and hierarchical vision transformers predicting superpixel-level expression^21^.

Computational models capable of learning correlations between image features and expression patterns could ultimately be implemented in the clinical setting to predict TIME characteristics from routine tissue samples, even when spatial omics data are lacking. While most prior work has focused on H&E images, our model Image2Count instead learns from low-plex immunofluorescence (IF) images, thereby incorporating cell-type-specific information in the extracted visual features, which is lacking in H&E images. Image2Count was trained and tested using data and images from different spatial omics platforms, including t-CyCIF^22^, CosMx^23^, and GeoMx^24^. While t-CyCIF and CosMx provide single-cell resolution, GeoMx yields ROI-level biomarker (proteins or genes) expression in regions typically comprising hundreds of cells. Consequently, GeoMx data in particular will benefit from deconvolution of “bulk” ROI measurements. Rather than predicting expression for image patches or ROIs, Image2Count predicts cell-level expression counts, enabling downstream single-cell analysis of predicted data using standard workflows. Furthermore, our design permits training on a relatively limited number (<1000) of ROIs while achieving competitive performance. We trained a ResNet^25^ model with SimCLR^26^ in a contrastive fashion to learn cell-level visual representations, which serve as graph nodes for a GNN that aggregates neighborhood information to predict cell expression. For comparison, we also trained on the same embeddings a linear prediction head for SimCLR pre-trained ResNet and a non-linear multi-layer Feed Forward (FFW) network with residual connections, both of which ignore spatial context. We further performed single-cell analysis of predicted cell expression and generated visualizations of cell expression overlaid images. Additionally, we correlated predicted single-cell and computationally aggregated pseudo-ROIs with true (visually based for t-CyCIF) expression of single cells and pseudo-ROIs of the CycIF and CosMx data, to assess how accurately the models capture single-cell and TIME expression patterns based solely on image information. Code, pipelines and tutorial can be found on our github: https://github.com/CancerTargetLab/Image2Count.

Image2Count contributes uniquely to the field by (1) enabling cell count predictions from mIF images, (2) allowing model training on limited spatial expression samples, (3) systematically evaluating performance as a function of spatial coverage, and (4) demonstrating the added value of cell-type-specific marker staining for predicting expression.

## Results

### Overview of the Image2Count approach

**Fig. 1:**
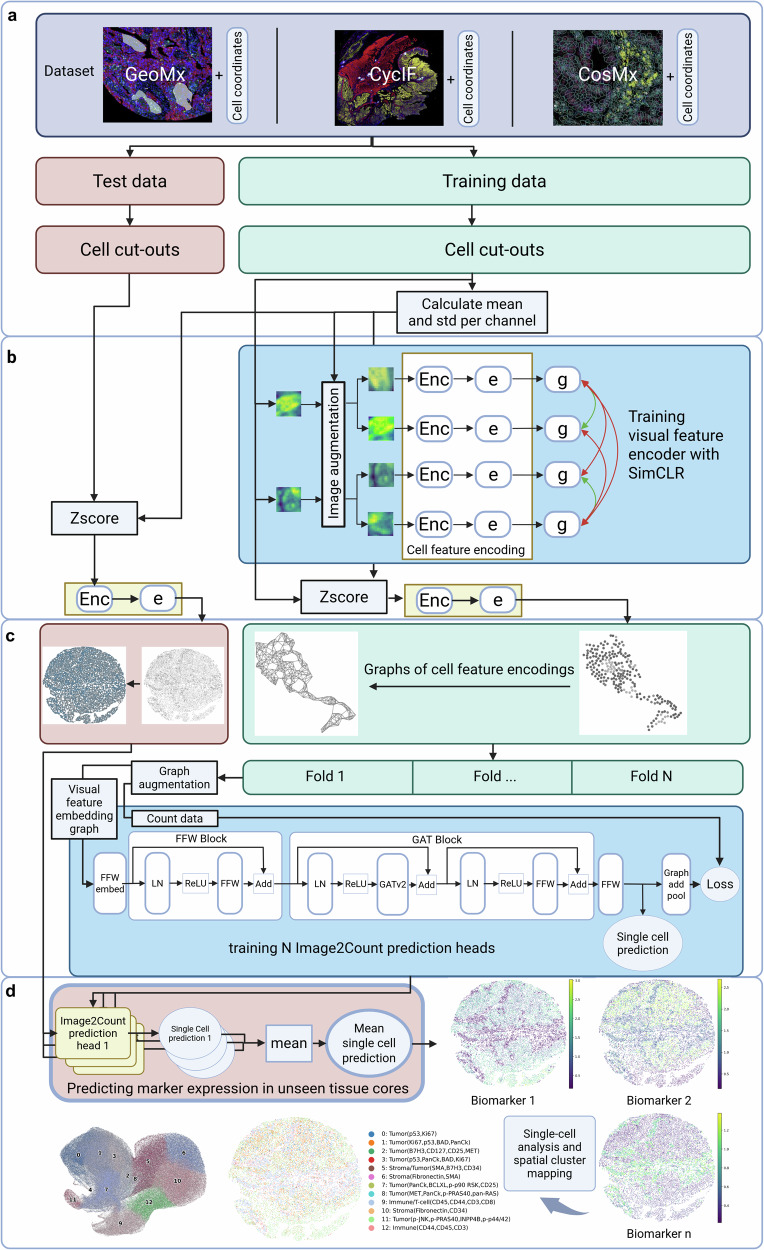
Overview of the Image2Count approach. Image2Count (**a**–**d**) was applied on three different datasets: GeoMx, t-CyCIF, and CosMx. **a** Each dataset, for which cell positions are known, is split into testing and training data over batches (instrument runs) with non-overlapping patient IDs. Each cell is cut-out in a square window. Mean and standard deviation per channel is calculated for the cell cut-outs from training data, and used in **b** to Zscore-normalize images from both training and test data. During pre-training, an encoder ResNet model is trained in a contrastive fashion via SimCLR on the training data to learn visual features of cell cut-outs. **c** From saved embeddings, spatial graph networks are built (per ROI), with nodes representing cells, and edges the spatial distance to the nearest neighbors. Training data is split over patients into N cross-validation folds. N Image2Count prediction heads, consisting of FFW Blocks or FFW and GAT Blocks, perform node feature learning, after which expression counts are predicted for each cell. During model training, predicted counts are summed per protein/transcript per ROI to calculate the loss. **d** The N Image2Count prediction heads estimate the cell expression of the test data. Mean prediction of all *N* models is calculated. Standard downstream analysis and celltype annotation is applied on predicted cell expression. Figure was created in Biorender.

Image2Count was trained and tested for performance in three datasets: An in-house GeoMx dataset from ovarian cancer (OC) with 4-plex IF images and 72-plex protein counts per ROI; a publicly available colorectal cancer (CRC) t-CyCIF dataset^27,28^ from which we created pseudo ROI counts for training purposes; and a publicly available 960-plex non-small cell lung cancer (NSCLC) transcriptomics CosMx dataset^23^ with 5-plex images. The workflow is visualized in Fig. 1. Models were trained for each data type separately. Data were split into test and training sets over data batches (instrument runs) with non-overlapping patients. For each dataset, a visual feature backbone was trained via SimCLR, sampling equally when not otherwise specified from K-Means clusters of centroid pixels of each cell cut-out, leading to a more equal cell class distribution and an oversampling of low-abundance phenotypes. After training the visual feature backbone on the training data, features of each cell cut-out were embedded into spatial cell graphs, with nodes being the learned visual features and edges the Euclidean distance to the six closest cells. Pseudo bulk-counts were constructed for the CosMx and t-CyCIF datasets by adding expression based on marker abundance of all cells in a given area to resemble GeoMx ROI data and train model predictions for cell expression. Training data was split over N cross-validation folds by patients (slides for CosMx), resulting in N Image2Count prediction heads which estimate single-cell expression of the test data. We compared the performance of a linear prediction head, from here referred to as ResNet for simplicity; Image2Count prediction heads consisting of purely residual FFW networks; and Image2Count prediction heads utilizing residual graph convolutions with attention (GAT) and FFW networks to predict cell expression from the constructed cell graph. For CosMx and t-CyCIF data, we also investigated model performance as a function of aggregated area size by creating multiple subgraphs per image, starting from a single cell and expanding *k* times and summing the true and predicted expression of the selected area. Merged predictions were obtained by calculating the mean prediction of the N Image2Count prediction heads.

### Benchmarking models with CosMx data

The publicly available CosMx NSCLC dataset^23^ consists of 8 samples from 5 tissues and donors. Two samples (lung 6 and 13) were used for testing, and the rest for cross-validation training, split over the remaining 6 slides. The samples had been stained for membrane, PanCK, CD45, CD3 and DAPI. Count data were obtained from a 960-plex CosMx RNA panel run on a CosMx SMI instrument prototype, detecting transcripts at a subcellular level and assigning them to detected cells. We created four pseudo ROIs with bulk count data for each field-of-view image (total 732) to train models.

#### Merged model predictions performed better than average cross-validation runs

Marker specific metrics (Spearman and Pearson correlation, Mutual Information (MI), MSE of log1p values) and sample specific metrics (Cosine Similarity, Jenson–Shannon divergence, structural similarity index (SSIM), rand index adjusted for chance (ARI) and normalized mutual information between two clusterings (NMI)) (Tables 1 and S1–S9) were generally higher when merging predictions from all cross-validation models by calculating the mean expression of each marker for each cell. When creating 1800 subgraphs starting from a single cell and expanding to a *k*-hop neighborhood, higher correlation for the summed expression over a larger number of cells was observed, with a significant increase when comparing the summed expression of 7 or more cells to single-cell correlations. Merged GAT Image2Count outperformed other models based on SSIM, MI, Spearman and Pearson correlation; however, merged FFW Image2Count performed better based on MSE and Jensen–Shannon divergence, and merged ResNet performed better based on Cosine Similarity. Average Pearson correlation coefficent and MI across cross-validation folds of GAT Image2Count was 0.067 ± 0.028 (Pearson), 0.018 ± 0.002 (MI) for single cells (0.097 (Pearson) 0.021 (MI) for merged predictions); 0.135 ± 0.045 (Pearson), 0.059 ± 0.010 (MI) for 1-hop subgraphs consisting of an average of 7.7 cells (0.185 (Pearson) 0.070 (MI) for merged predictions); and 0.189 ± 0.049 (Pearson), 0.096 ± 0.021 (MI) for 2-hop subgraphs consisting of an average of 21.3 cells (0.250 (Pearson) 0.116 (MI) for merged predictions). Hence, for 1 and 2-hop subgraphs, the performance was at similar level based on Pearson correlation and MI values to those reported by Wang et al.^13^ who benchmarked several methods for predicting spatial gene expression from H&E images, with the best performing method achieving a Pearson correlation of 0.28 and MI of 0.06 and SIM of 0.22 for spatial transcriptomic spots of 150–200 μm diameter, which also should correspond to a higher number of cells than our 2-hop neighborhoods with an average of 21.3 cells. Merged GAT Image2Count predictions performed better on higher abundant markers, or those with a higher signal-to-noise ratio (SNR). Mean edge length of cells did not have a strong impact on performance; however, for regions with high mean edge length, performance was worse based on Jensen–Shannon divergence (Fig. S1).

**Table 1:** Image2Count performance on CosMx data

Model num hops	PCC *↑*	SCC *↑*	MI *↑*	MSE *↓*	CosSim *↑*	js_div *↓*	SSIM *↑*	ARI *↑*	NMI *↑*
ResNet sc FFW I2C sc GAT I2C sc	0.067 ± 0.104	0.079 ± 0.127	0.017 ± 0.039	0.128 ± 0.442	**0.503 ± 0.139**	**0.389 ± 0.090**	0.326	**0.234**	**0.401**
	0.094 ± 0.089	0.120 ± 0.116	0.021 ± 0.039	**0.124 ± 0.436**	0.487 ± 0.140	0.392 ± 0.087	**0.332**	0.188	0.366
	**0.097 ± 0.096**	**0.122 ± 0.117**	**0.021 ± 0.041**	0.127 ± 0.435	0.490 ± 0.135	0.389 ± 0.088	0.330	0.163	0.355
ResNet dca sc FFW I2C dca sc GAT I2C dca sc	0.128 ± 0.149	0.141 ± 0.190	0.058 ± 0.069	0.091 ± 0.502	**0.454 ± 0.205**	0.194 ± 0.076	0.596	**0.197**	**0.378**
	0.213 ± 0.154	0.296 ± 0.178	**0.104 ± 0.071**	**0.089 ± 0.499**	0.440 ± 0.202	0.194 ± 0.077	0.604	0.166	0.346
	**0.221 ± 0.159**	**0.303 ± 0.176**	0.103 ± 0.074	0.090 ± 0.497	0.442 ± 0.200	**0.193 ± 0.078**	**0.608**	0.148	0.337
ResNet 1-hop FFW I2C 1-hop GAT I2C 1-hop	0.131 ± 0.169	0.144 ± 0.192	0.061 ± 0.095	0.526 ± 1.058	**0.660 ± 0.097**	0.203 ± 0.060	0.383	0.294	0.494
	0.183 ± 0.1444	0.213 ± 0.161	0.067 ± 0.086	0.509 ± 1.021	0.642 ± 0.098	0.203 ± 0.059	0.379	**0.317**	**0.500**
	**0.185 ± 0.149**	**0.214 ± 0.167**	**0.070 ± 0.091**	**0.525 ± 1.058**	0.643 ± 0.094	**0.202 ± 0.059**	**0.386**	0.263	0.472
ResNet dca 1-hop FFW I2C dca 1-hop GAT I2C dca 1-hop	0.205 ± 0.214	0.222 ± 0.239	0.113 ± 0.106	0.347 ± 1.179	**0.539 ± 0.185**	0.141 ± 0.050	0.589	0.299	**0.493**
	0.310 ± 0.210	0.363 ± 0.216	0.155 ± 0.099	**0.338 ± 1.176**	0.522 ± 0.180	0.140 ± 0.051	0.589	**0.300**	0.487
	**0.316 ± 0.215**	**0.371 ± 0.218**	**0.157 ± 0.104**	0.338 ± 1.176	0.523 ± 0.178	**0.140 ± 0.052**	**0.598**	0.260	0.464
ResNet 2-hop FFW I2C 2-hop GAT I2C 2-hop	0.169 ± 0.200	0.178 ± 0.219	0.099 ± 0.128	0.783 ± 1.427	**0.693 ± 0.087**	0.147 ± 0.044	0.457	**0.346**	**0.506**
	0.246 ± 0.170	0.272 ± 0.182	0.112 ± 0.112	**0.747 ± 1.354**	0.675 ± 0.087	0.147 ± 0.044	0.455	0.288	0.494
	**0.250 ± 0.176**	**0.277 ± 0.188**	**0.116 ± 0.117**	0.768 ± 1.421	0.676 ± 0.084	**0.146 ± 0.044**	**0.463**	0.293	0.488
ResNet dca 2-hop FFW I2C dca 2-hop GAT I2C dca 2-hop	0.235 ± 0.237	0.244 ± 0.255	0.138 ± 0.120	0.498 ± 1.474	**0.557 ± 0.172**	0.131 ± 0.045	0.635	**0.346**	**0.477**
	0.351 ± 0.229	0.389 ± 0.229	0.181 ± 0.105	0.477 ± 1.461	0.542 ± 0.166	0.130 ± 0.045	0.636	0.264	0.461
	**0.361 ± 0.236**	**0.404 ± 0.233**	**0.192 ± 0.114**	**0.475 ± 1.463**	0.542 ± 0.165	**0.130 ± 0.046**	**0.646**	0.297	0.473
ResNet 3-hop FFW I2C 3-hop GAT I2C 3-hop	0.174 ± 0.221	0.179 ± 0.238	0.130 ± 0.142	0.902 ± 1.658	**0.707 ± 0.081**	0.124 ± 0.038	0.506	**0.375**	0.482
	0.276 ± 0.186	0.299 ± 0.195	0.147 ± 0.122	**0.849 ± 1.552**	0.688 ± 0.079	0.124 ± 0.037	0.504	0.296	0.482
	**0.283 ± 0.194**	**0.308 ± 0.203**	**0.155 ± 0.128**	0.871 ± 1.634	0.690 ± 0.077	**0.123 ± 0.038**	**0.514**	0.301	**0.492**
ResNet dca 3-hop FFW I2C dca 3-hop GAT I2C dca 3-hop	0.222 ± 0.254	0.225 ± 0.270	0.149 ± 0.125	0.581 ± 1.622	**0.569 ± 0.162**	0.126 ± 0.042	0.660	**0.393**	**0.494**
	0.360 ± 0.237	0.389 ± 0.236	0.193 ± 0.108	0.548 ± 1.580	0.553 ± 0.157	0.125 ± 0.042	0.661	0.292	0.478
	**0.373 ± 0.247**	**0.408 ± 0.242**	**0.207 ± 0.118**	**0.544 ± 1.583**	0.553 ± 0.155	**0.124 ± 0.043**	**0.672**	0.283	0.485
ResNet 5-hop FFW I2C 5-hop GAT I2C 5-hop	0.198 ± 0.246	0.199 ± 0.261	0.192 ± 0.151	0.927 ± 1.816	**0.719 ± 0.073**	0.107 ± 0.034	0.579	0.229	0.426
	0.335 ± 0.207	0.358 ± 0.211	0.220 ± 0.125	**0.860 ± 1.67**	0.700 ± 0.070	0.107 ± 0.033	0.578	0.236	0.424
	**0.345 ± 0.218**	**0.373 ± 0.221**	**0.232 ± 0.131**	0.879 ± 1.762	0.702 ± 0.068	**0.106 ± 0.035**	**0.589**	**0.247**	**0.431**
ResNet dca 5-hop FFW I2C dca 5-hop GAT I2C dca 5-hop	0.237 ± 0.273	0.234 ± 0.287	0.177 ± 0.139	0.640 ± 1.685	**0.582 ± 0.148**	0.120 ± 0.039	0.690	0.258	0.455
	0.399 ± 0.246	0.422 ± 0.244	0.229 ± 0.113	0.597 ± 1.652	0.567 ± 0.144	0.119 ± 0.038	0.692	0.270	0.455
	**0.416 ± 0.259**	**0.447 ± 0.254**	**0.254 ± 0.127**	**0.591 ± 1.656**	0.567 ± 0.142	**0.119 ± 0.040**	**0.703**	**0.302**	**0.465**

Pearson correlation (PCC), spearman correlation (SCC), mutual information (MI), mean squared error of log1p expression (MSE), cosine similarity (CosSim), Jenson-Shannon divergence (js_div), structural similarity index (SSIM), rand index adjusted for chance (ARI) and normalized mutual information between two clusterings (NMI) of merged cross-validation predictions of linear evaluation head of the ResNet visual feature backbone, FFW Image2Count (I2C) and graph-based Image2Count models to counts of the test data from the CosMx dataset, from average per-molecule or per cell/niche metrics. Standard deviation is specified over molecules (PCC, SCC, MI, MSE) and over cells/niches (CosSim, js_div) where possible. Merged predictions were obtained by calculating the mean prediction over all cross-validation runs. DCA signifies correlation of previous predicted counts to imputed count data using DCA (deep-count autoencoder). *K* hop signifies a k-hop subgraph created from a central cell, expanding the graph to neighboring nodes *k* times. 36 (total 1800) subgraphs were created per image. 1-hop subgraphs consist on average of 7.7 cells, 2-hop subgraphs of 21.3 cells, 3-hop subgraphs of 43.0 cells, and 5-hop subgraphs of 109.7 cells. Best performing model per metric per *K* hop is highlighted in bold.

#### Biologically meaningful activity is recovered in predicted data

Enrichment analysis was performed to identify optimal niche size for Image2Count predictions to recover biological processes. Enrichment was calculated per cell/niche, on predicted and true counts, respectively. Based on previous single-cell or k-hop niche clustering, we identified the top 5 pathways enriched per cluster, and filtered those by adjusted *p*-value below 0.05. Next, we calculated the percentage of top 5 pathways per cluster in the predicted data appearing in the same cluster in the true data. As we had two clusterings (based on predicted and on true counts), we divided the two coverage percentages by 2 and added them.

We performed this analysis on transcription factors from CollecTRI^29^, PROGENy^30^, msgidb Hallmark pathways^31^, Reactome^32^ and KEGG^33^ pathways (Tables S10–S14). Of the merged models, the following performance was observed: The ResNet model performed best on CollecTRI (222 total transcription factors) on the single-cell level, with a coverage of 0.087. Coverage was higher for PROGENy (13 pathways), with the best-performing model being the FFW Image2Count prediction head at 2-hop neighborhoods, with a coverage of 0.5 (0.385 on the single-cell level). FFW Image2Count prediction head also performed the best for Hallmark pathways (30 total pathways) at 1-hop neighborhoods, with a coverage of 0.405 (0.390 for single-cell level). GAT Image2Count prediction head performed the best for the top 5 Reactome pathways (106 total pathways) at 2-hop neighborhoods, with a coverage of 0.176 (0.153 for single-cell level). The top-performing model for KEGG pathways (48 total pathways) was the GAT Image2Count prediction head at 3-hop neighborhoods, with a coverage of 0.29 (0.183 for single-cell level). Based on these results, 2-hop neighborhoods were selected as optimal niche size for recovering the most biologically meaningful information.

To identify how well Image2Count recovers cell types, we calculated enrichment scores for lung cell types (*n* = 44) obtained from CellMarker2.0^34^ and calculated top 1, top 3 and top 5 coverage metrics (Table S15). The merged GAT Image2Count prediction head outperformed at all thresholds, with a top 1 coverage of 0.146, top 3 coverage of 0.276 and a top 5 coverage of 0.252. Top 1 coverage correctly recovered T-cell and macrophage clusters, top 3 additionally assigned CD8+ T cell, basal cell, dendritic cell, cancer cell, myofibroblast, stromal, monocyte and fibroblast clusters, and top 5 additionally assigned cancer stem cell cluster correctly. It should be noted that automatic cell typing of CosMx data is inherently challenging, and that our approach was selected for ease of reproducibility. Correctly identified transcription factors, pathways and cell types of merged GAT Image2Count are listed in Tables S16–S21.

#### Performance was improved with data imputation

Like other spatial transcriptomics platforms, CosMx suffers from low detection accuracy of low-abundance transcripts^35^. In the current CosMx dataset, an average of 265 transcripts were associated with every cell. We hypothesized that imputation of missing values of dropout events of the raw counts might provide a more accurate estimate of model performance. The deep count autoencoder (DCA)^36^ was selected as a method to estimate false dropout events and impute missing values. We compared the same model predictions already presented, learned from raw counts, to true counts imputed via DCA. Performance was significantly improved compared to non-imputed counts, based on higher SSIM, MI, Pearson and Spearman correlation and lower MSE and Jensen–Shannon divergence (Tables S1–S4, S6-S7 and Table 1), while cosine similarity, ARI and NMI indicated an overall worse performance on imputed data (Tables S5, S8 and S9). Average Pearson correlation and MI across cross-validation folds of GAT Image2Count was 0.146 ± 0.070 (Pearson), 0.086 ± 0.001 (MI) for single cells (0.221 (Pearson), 0.103 (MI) for merged predictions); 0.228 ± 0.074 (Pearson), 0.128 ± 0.027 (MI) for 1-hop subgraphs consisting of an average of 7.7 cells (0.316 (Pearson), 0.157 (MI) for merged predictions); and 0.274 ± 0.066 (Pearson), 0.144 ± 0.044 (MI) for 2-hop subgraphs consisting of an average of 21.3 cells (0.374 (Pearson) 0.192 (MI) for merged predictions).

While zero-inflated counts can lead to underestimating performance metrics, imputed counts could potentially overestimate correlation metrics. Our results imply that the prediction of raw CosMx counts might underestimate actual model performance, as Image2Count (trained on raw bulk count data) predictions aligned better with DCA imputed counts for all marker-level metrics, with two out of five sample-level metrics outperforming similarly, demonstrating that both models’ predictions learn potentially similar underlying marker expressions. However, three of five sample-level metrics and all coverage metrics indicated underperformance for DCA imputed counts, indicating that DCA and Image2Count recover cellular identities with slight differences.

### Benchmarking models with t-CyCIF data

The publicly available CRC Atlas 2022^27,28^ consists of data and images from whole tissue sections from 17 CRC patients, analyzed using t-CyCIF for high-resolution staining of multiple (*n* = 25) protein markers. To resemble the GeoMx data, which is based on ROIs selected from 4-plex IF, visual representation learning on the t-CyCIF data was based on images from the same four markers (DNA, Keratin, CD45 and CD8a) only. In addition, visual representation learning was also conducted using two markers only (DNA and Keratin) to investigate the performance compared to when immune markers (CD45 and CD8) were included for the downstream task. Whole slide images from CRC samples 3, 5 and 13 were used as test data. Training/validation data were split over 10 cross-validation folds by Patient ID to predict expression. Spatial cell graphs were created for each of the areas (*n* = 49 per whole slide image).

#### Prediction improves with neighborhood size, number of markers used for training, and visual information

Performance for predicted counts was calculated in relation to image-based single-cell counts of the test data (Table 2). In addition, 900 equally distributed subgraphs were created per image, evaluating performance at different niche sizes (Tables S1–S9).

**Table 2:** Image2Count performance on t-CyCIF data

sc t-CyCIF	PCC *↑*	SCC *↑*	MI *↑*	MSE *↓*	CosSim *↑*	js_div *↓*	SSIM *↑*	ARI *↑*	NMI *↑*
ResNet	0.221 ± 0.146	0.343 ± 0.232	0.140 ± 0.138	1.649 ± 2.879	0.829 ± 0.143	0.086 ± 0.051	0.683	0.322	0.507
FFW I2C	0.416 ± 0.230	0.528 ± 0.214	0.302 ± 0.247	0.959 ± 2.011	**0.852 ± 0.140**	0.072 ± 0.048	0.787	0.320	0.523
GAT I2C	**0.423 ± 0.222**	**0.534 ± 0.215**	**0.303 ± 0.236**	**0.927 ± 1.921**	0.851 ± 0.139	**0.071 ± 0.047**	**0.797**	**0.330**	**0.540**
ResNet 2 Channels	0.155 ± 0.126	0.269 ± 0.251	0.105 ± 0.125	1.955 ± 3.602	0.772 ± 0.194	0.112 ± 0.065	0.623	0.263	0.442
FFW I2C 2 Channels	0.322 ± 0.238	0.444 ± 0.249	0.244 ± 0.267	1.102 ± 2.243	0.833 ± 0.154	0.083 ± 0.053	0.747	0.250	0.454
GAT I2C 2 Channels	0.335 ± 0.233	0.446 ± 0.249	0.250 ± 0.265	1.073 ± 2.175	0.835 ± 0.158	0.081 ± 0.054	0.756	0.252	0.471
ResNet no KM	0.161 ± 0.131	0.330 ± 0.190	0.113 ± 0.115	1.918 ± 3.438	0.787 ± 0.162	0.104 ± 0.057	0.648	0.289	0.431
FFW I2C no KM	0.343 ± 0.240	0.456 ± 0.231	0.221 ± 0.198	1.194 ± 2.511	0.781 ± 0.178	0.102 ± 0.060	0.730	0.220	0.409
GAT I2C no KM	0.331 ± 0.227	0.429 ± 0.239	0.201 ± 0.182	1.163 ± 2.320	0.768 ± 0.181	0.104 ± 0.061	0.742	0.304	0.473
ResNet 8 μm	0.246 ± 0.178	0.358 ± 0.220	0.146 ± 0.159	1.901 ± 3.915	0.752 ± 0.190	0.118 ± 0.069	0.646	0.232	0.399
FFW I2C 8 μm	0.378 ± 0.249	0.456 ± 0.230	0.241 ± 0.206	1.096 ± 2.276	0.821 ± 0.156	0.087 ± 0.054	0.755	0.186	0.391
GAT I2C 8 μm	0.387 ± 0.234	0.454 ± 0.243	0.239 ± 0.201	1.059 ± 2.104	0.803 ± 0.165	0.088 ± 0.056	0.771	0.216	0.407
ResNet 20 μm	0.281 ± 0.184	0.412 ± 0.228	0.191 ± 0.193	1.680 ± 3.482	0.796 ± 0.167	0.095 ± 0.056	0.703	0.319	0.491
FFW I2C 20 μm	0.412 ± 0.262	0.494 ± 0.240	0.298 ± 0.274	1.046 ± 2.298	0.838 ± 0.150	0.079 ± 0.051	0.769	0.238	0.463
GAT I2C 20 μm	0.420 ± 0.242	0.512 ± 0.233	0.294 ± 0.247	0.972 ± 2.023	0.833 ± 0.152	0.077 ± 0.051	0.791	0.274	0.474

Pearson correlation (PCC), spearman correlation (SCC), mutual information (MI), mean squared error of log1p expression (MSE), cosine similarity (CosSim), Jenson-Shannon divergence (js_div), structural similarity index (SSIM), rand index adjusted for chance (ARI) and normalized mutual information between two clusterings (NMI) of merged cross-validation predictions of linear evaluation head of the ResNet visual feature backbone, FFW Image2Count (I2C) and graph-based Image2Count models to counts of the test data from the t-CyCIF dataset, from average per-molecule or per cell/niche metrics. Standard deviation specified over proteins (PCC, SCC, MI, MSE) and over cells/niches (CosSim, js_div) where possible. Merged predictions were obtained by calculating the mean prediction over all cross-validation runs. Single cell metrics are shown here. Detailed information on average cross-validation performance and niche size is provided in Tables S1–S9. Best performing model per metric is highlighted in bold.

The 4-channel models (learning from DNA, Keratin, CD45 and CD8) consistently outperformed the 2-channel models (learning from DNA and Keratin only) (Tables 2 and S1–S9). Unsurprisingly, model access to visual marker information greatly helped count prediction of these specific markers (e.g., from mean Spearman values of 0.233 for the DNA/Keratin merged GAT model to 0.694 for the 4-channel merged GAT model for CD8a), as well as for markers for which the model did not have visual marker information (e.g., Spearman values from 0.303 to 0.457 for PD-1). The merged GAT Image2Count model marginally outperformed the merged FFW Image2Count model based on all metrics when training on 4 image channels, which indicates that the immediate cell neighbors are the most impactful for prediction of a cell’s expression. As each cell cut-out was based on a 50 × 50 μm area, more than one cell is likely to be included in a cut-out (mean edge distance of 30 μm in the test dataset). It is therefore reasonable to hypothesize that the most relevant information for a single cell’s expression is already contained within the corresponding cut-out, and that added context of surrounding cells through graph convolutions in general only minimally improves prediction of expression. The 4-channel GAT Image2Count model displayed a higher standard deviation for single-cell performance metrics over all cross-validation folds compared to the FFW Image2Count model, indicating that graph-based models are more data-dependent and might need more diverse and larger quantities of data for optimized learning. For some markers, a graph-based approach increased the average prediction (e.g., CD20 Spearman values for merged FFW Image2Count model: 0.483; graph model: 0.513 (Table S22)), thus spatial context might be more informative for the prediction of certain markers. Spearman correlation increased dramatically when including more than one cut-out, to >0.64 for 1-hop subgraphs consisting of 7.8 cut-outs on average. Further increasing the number of cells up to the training area size (~438.5 cells) continuously improved the performance (Tables S1–S9). This suggests that the models predict expression of all cells contained within a cell cut-out, not just the central one, i.e., learning averaged expressions for a number of cells in each cell cut-out image. Therefore, correlation at the single-cell level is lower compared to when describing an immediate area. Experiments where low-abundance K-Means pixel clusters were not oversampled during the training of the 4-channel visual feature extraction module resulted in inferior performance compared to oversampling low-abundance K-Means pixel clusters. Oversampling low-abundance phenotypes (e.g., CD8+ T-cells) during training of the visual feature module consequently helped significantly in their count prediction when using Image2Count. Creating a consensus prediction by selecting the mean of each predicted cell expression over all cross-validation runs improved single-cell correlation. Window size of the cell cut-out also impacted performance. While larger cut-outs seemed to improve correlation of most predicted markers (e.g., Desmin Spearman correlation 0.157 at 8 μm, 0.240 at 20 μm, 0.378 at 50 μm for the merged GAT Image2Count), other markers performed worse (e.g., CD8a Spearman correlation 0.575 at 8 μm, 0.767 at 20 μm, 0.694 at 50 μm for the merged GAT Image2Count), potentially as a consequence of the tissue distribution of the individual markers.

#### Analysis of predicted single-cell expression identifies spatial patterns and specific cell clusters

**Fig. 2:**
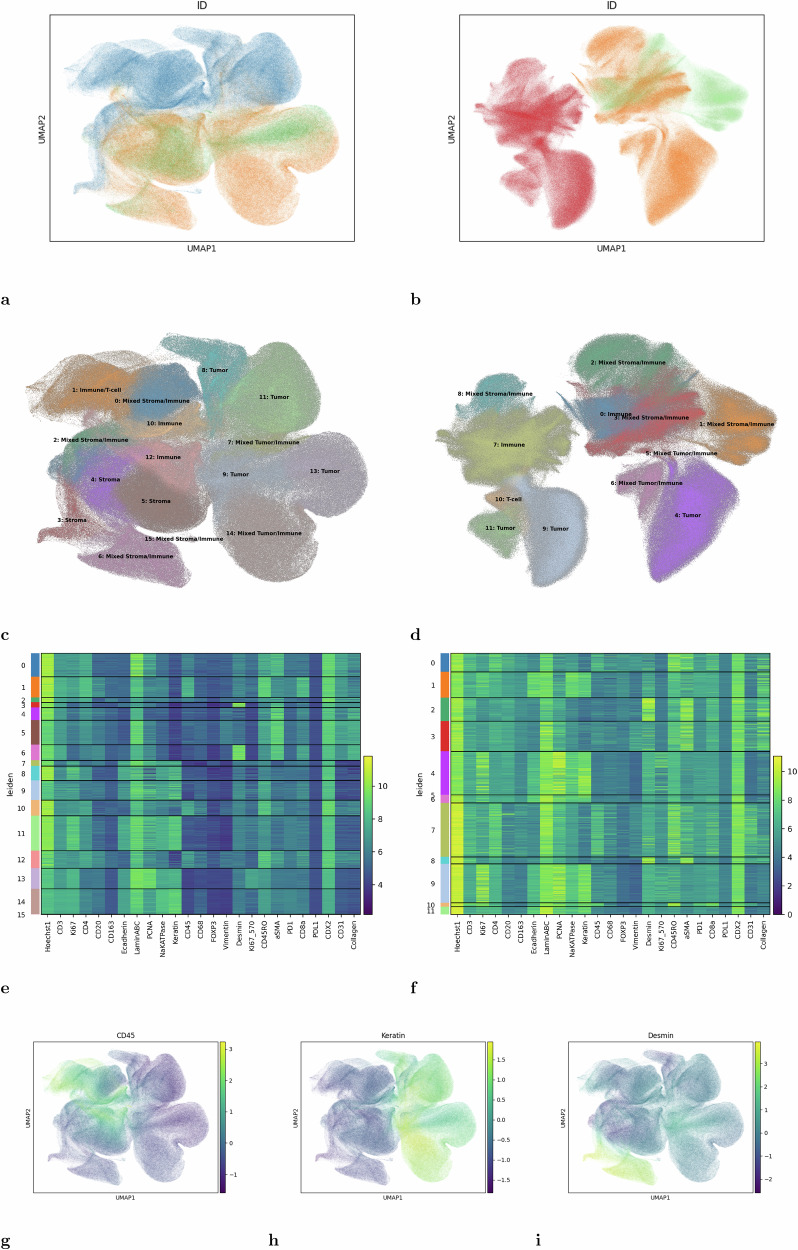
Single-cell analysis of predicted protein expression by a GAT Image2Count model and visual based counts of the test t-CyCIF data. **a** UMAP of predicted cell count colored by Patient ID; **b** UMAP of original image-based cell counts colored by Patient ID; **c** UMAP of predicted cell counts colored by Leiden clusters; **d** UMAP of original image-based cell counts colored by Leiden clusters; **e** heatmap of predicted cell counts by Leiden clusters; **f** heatmap of original image-based cell counts by Leiden clusters; **g**, **h**, **i** UMAP based on predicted cell counts colored by normalized and scaled expression of selected markers of immune (CD45), tumor (Keratin) and stroma (Desmin) cells, respectively.

We performed single-cell analysis using scanpy^37^. In the t-CyCIF data, patients were separated into mainly two large clusters, as shown both for predicted (Fig. 2a) and original (image-based) data (Fig. 2b), with differences being explained visually through varying mean intensities of markers, indicating batch effects (Figs. S2 and S3). Leiden clusters visualized in UMAPs (Fig. 2c, d) were annotated based on Wilcoxon rank-sum predictions (Figs. S4 and S5), revealing clear separation of tumor, stroma and immune clusters, and identification of cell-type-specific clusters based on top-ranked predicted markers.

**Fig. 3:**
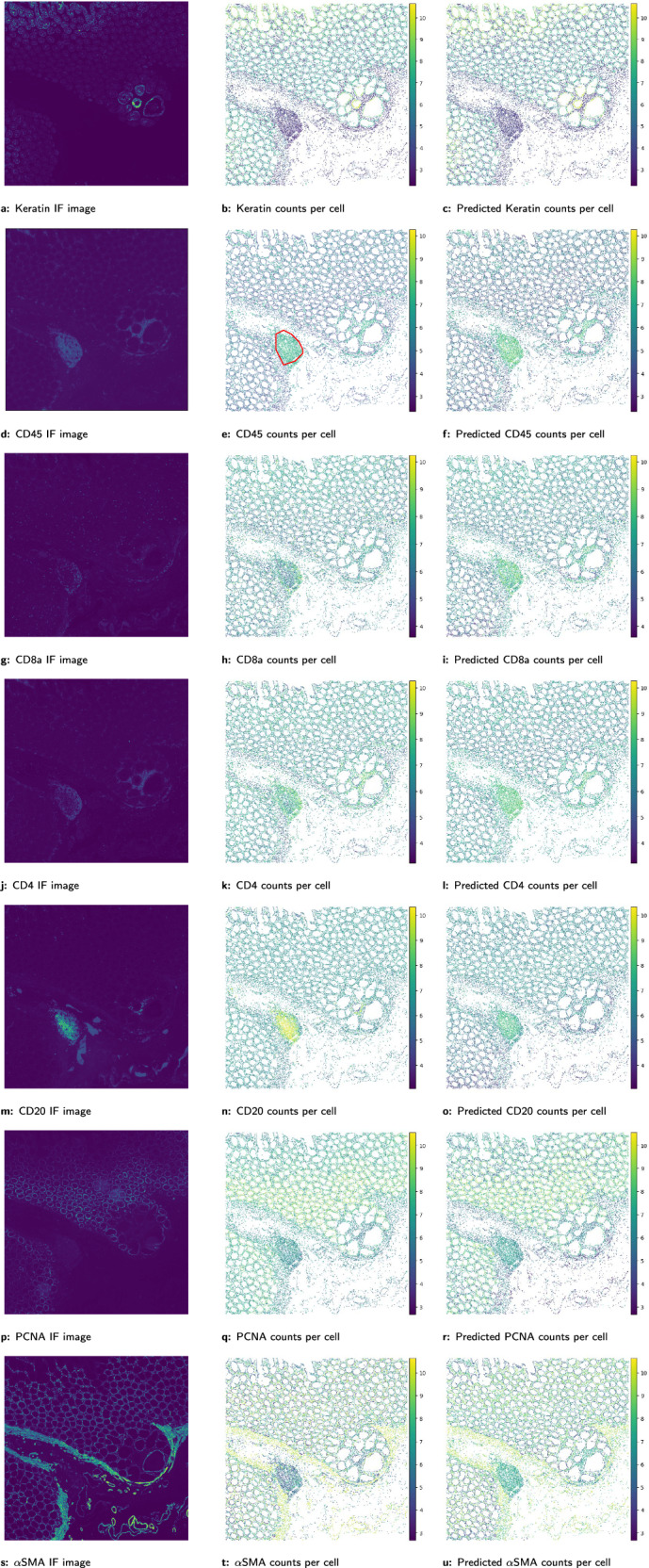
Visualization of raw and Image2Count predicted data. Representative CRC tissue section from the t-CyCIF data (sample CRC03), showing selected markers with the original IF image, the normalized total and log1p transformed image-based cell expressions, and Image2Count predicted cell expressions, respectively (**a**-**u**). Images from the Keratin (**a**), CD45 (**d**), CD8a (**g**) and one of the Hoechst (DNA) channels were the visual channels that the GAT Image2Count model had access to. The tissue area contains a tertiary lymphoid structure, outlined in red in **e**. Protein counts per cell were normalized and log1p transformed. Brightness was increased for **d** for visualization purposes.

**Fig. 4:**
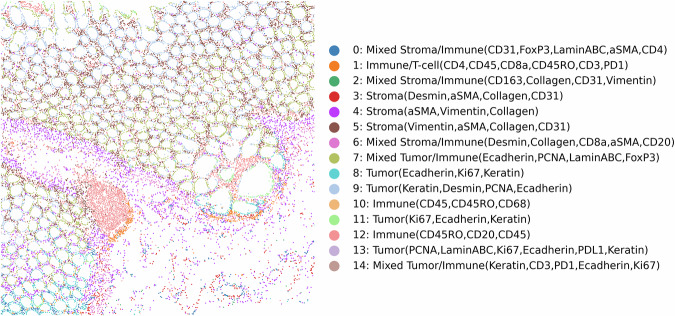
Predicted clusters overlayed the CRC03 tissue section. Coarse annotations with top biomarkers based on ranked prediction values are noted for each cluster. For clusters with several biomarkers showing relatively equal prediction values, more than the top 3 are noted. Predicted marker ranks per cluster is available in Figs. S2 and S3.

To compare predicted spatial patterns with true expression derived from the raw IF images, normalized counts were plotted for selected markers in a representative image (Patient/Slide CRC03) which was part of the test set for the trained model (Fig. 3). In general, predicted cell counts had lower signal peaks than true cell counts, and thus a more “averaged” prediction across the tissue. Hence, small differences in the image color spectrum for higher log1p cell expression resulted in a relatively larger numerical difference. We hypothesize that the model tends to learn more average expressions when not overly penalized by a higher L1 loss. This implies that it more easily learns an average expression for all cells/a cell type, instead of learning the underlying cell expression, especially when considering particularly low-abundant markers. This difference is also evident in the heatmaps (Fig. 2e, f). Despite this variation, similar patterns and structures were observed for predicted cell counts and original cell counts when plotting total cell expression instead of log1p cell expression. Figure 3d–f shows that the CD45 expression in the larger tissue structures, including a tertiary lymphoid structure (TLS) on the tumor border, as well as distinct intra-tumoral immune localization, were correctly learned. The highest expression of CD20 was also correctly localized to the TLS (Fig. 3m–o), exemplifying a marker for which the model did not have visual information when training. The scale of the prediction was, however, lower than the actual counts, explained by the low abundance of CD20+ cells outside of the TLS. Despite the scarcity of CD20+ cells to learn from, areas of B-cell infiltration inside the tumor also seemed to have been accurately predicted. The model, however, largely failed to recapitulate intra-TLS differential spatial distribution of CD20, CD4 and CD8a. The predicted T-cell expression (CD8a and CD4) was more uniform across the TLS compared to the true expression, which showed higher T-cell density towards the TLS boundary (Figure 3g–i, j–l). This indicates that the model is struggling to discriminate between different types of lymphocytes, which are also morphologically highly similar. When reducing the window size of the cell cut-outs to 20 × 20 μm, the model was able to better identify CD8-positive cells, while still predicting uniform CD4 expression within the TLS (Fig. S6). Although the model had visual information of CD8a, other morphological features that are shared between lymphocytes might have driven this particular prediction. In general, TLSs were uncommon in the CRC images, and thus likely underrepresented in the training data, which may explain a generally lower accuracy in cell predictions within this structure. In contrast, predicted expression of tumor markers such as PCNA showed that specific tumor localization had been learned (Fig. 3p–r). Similarly, the primary localization of *α*SMA at the tumor-stroma margin was correctly predicted (Fig. 3s–u). The spatial distribution of Leiden clusters from predicted counts was also visualized (Fig. 4). Cluster 12, which was annotated as primarily B-cells (top predicted markers CD45RO, CD20, and CD45), was clearly localized in the TLS and lymphocyte-infiltrating tumor regions. Compared to true counts, some Cluster 12 cells are, however, more likely to be T-cells, which again indicates the limited ability of the model to clearly discriminate between lymphocyte subsets. Cluster 1 was annotated as the main T-cell cluster (top predicted markers CD4, CD45, CD8a, CD45RO, CD3, PD-1) and was accurately located at the TLS boundary as well as parts of the tumor margin. Individual T-cell subtypes were not clearly discriminated by the model; however, FoxP3 had high prediction for Cluster 0 (top predicted markers CD31, FoxP3, LaminABC, *α*SMA, CD4) and Cluster 7 (top predicted markers E-cadherin, PCNA, LaminABC and FoxP3) (Fig. S5), again demonstrating how primarily spatial co-localization of cell types was captured by the model rather than distinct single-cell expression. Increasing the number of clusters to *n* = 39 indicated individual clusters representing e.g., Tregs and PD-1+ infiltrating T-cells; however, UMAP separation of such a high number of clusters was not convincing (Figs. S7 and S8). Multiple clusters were coarsely annotated as tumor cells. While Keratin and E-cadherin in general had high prediction scores in all tumor clusters, the predicted expression of PCNA and Ki67 varied, with Cluster 7 clearly representing PCNA-positive cells, whose spatial localization correlated well with true PCNA staining (Fig. 3p). Cluster 11 had high prediction scores for Ki67, thus representing proliferating tumor cells. The spatial localization of this cluster correlated well with the true distribution of Ki67 (point-biserial correlation of 0.45).

### Benchmarking models with GeoMx data

Image and count GeoMx data were collected from 254 patients, with 636 4-plex IF images and 72-plex protein counts from corresponding ROIs obtained and normalized using GeoMx workflows^38^. Visual representations were learned and extracted from all four image channels: Syto13 (DNA), PanCk (tumor), CD45 (immune cells) and CD8 (cytotoxic T-cells). Cells were cut out in a 30 × 30 pixel window, corresponding to an area of 12 × 12 μm. The 10 TMA slides were split into a training and test set, seven for training (486 ROIs) and three for testing (150 ROIs). The data used for training the visual representation model were split over patients for tenfold cross-validation to predict cell expression. Larger areas on slide 1C54 for which no GeoMx data had been collected were used in addition to GeoMx ROIs of the test split for inference.

#### Image2Count`s performance is marker dependent

Performance metrics were based on GeoMx ROI counts and the per-ROI summed predictions of model runs (Tables 3 and S1–S9). Metrics were calculated on merged model predictions and the mean over all cross-validation folds. Different cell cut-out sizes were also considered. Merged FFW Image2Count performed the best based on the majority of metrics, with 12 × 12 μm being identified as the optimal cut-out size, based on investigating the per-cell expression of CD45 and CD8 together with their corresponding staining (Figs. S9 and S10). Therefore, we moved forward with the merged prediction of the 12 × 12 μm cut-out FFW Image2Count model run. When filtering for predictions with Benjamini–Hochberg adjusted *p*-values *p* < 0.05, mean Spearman values were 0.314 ± 0.140 over all predicted proteins, with 59 of 72 markers remaining after filtering. The 10 best performing markers were CD45, 0.65; PanCk, 0.58; CD3, 0.55; CD8, 0.54; CD14, 0.53; CD34, 0.53; CD44, 0.50; Fibronectin, 0.49; CD4, 0.47; CD11c, 0.44; CD163: 0.43, with *p*-values approaching 0 (Table S23). Of note, CTLA4 performed the worst, with negatively correlated predictions (Spearman correlation -0.32). PanCk, CD45 and CD8, for which the model had visual information, were among the top 10 predicted markers. However, other markers, in particular immune markers including CD14, CD3 and CD4, as well as stromal markers including CD34 and Fibronectin, showed high correlation for predicted expression as well. Intuitively, visual information for a specific marker should help predictions; however, staining quality and efficacy of the antibody panel used to generate GeoMx counts also impact prediction accuracy. Tumor heterogeneity of this mixed histology cohort might also explain why immune and stroma markers were more represented among the top correlated markers compared to tumor markers.

**Table 3:** Image2Count performance on GeoMx data

ROI GeoMx	PCC *↑*	SCC *↑*	MI *↑*	MSE *↓*	CosSim *↑*	js_div *↓*	SSIM *↑*	ARI *↑*	NMI *↑*
ResNet 8*μ*m	0.141 ± 0.250	0.178 ± 0.229	0.058 ± 0.078	2.553 ± 4.186	0.789 ± 0.156	0.093 ± 0.052	0.478	0.120	0.228
FFW I2C 8*μ*m	0.243 ± 0.177	**0.277 ± 0.161**	**0.067 ± 0.069**	0.759 ± 1.316	0.804 ± 0.146	0.065 ± 0.039	**0.804**	0.175	0.317
GAT I2C 8*μ*m	0.017 ± 0.182	0.010 ± 0.164	0.024 ± 0.040	1.039 ± 1.665	0.754 ± 0.175	0.090 ± 0.047	0.729	0.006	0.061
ResNet 12*μ*m	0.236 ± 0.187	0.261 ± 0.173	0.055 ± 0.068	**0.750 ± 1.299**	**0.808 ± 0.137**	**0.064 ± 0.037**	0.796	0.151	0.289
FFW I2C 12*μ*m	**0.246 ± 0.174**	0.277 ± 0.154	0.059 ± 0.060	0.765 ± 1.341	0.806 ± 0.141	0.066 ± 0.038	0.802	0.159	0.305
GAT I2C 12*μ*m	0.217 ± 0.178	0.245 ± 0.165	0.055 ± 0.056	0.758 ± 1.345	0.805 ± 0.137	0.067 ± 0.037	0.797	**0.189**	**0.339**
ResNet 20*μ*m	0.201 ± 0.195	0.242 ± 0.186	0.067 ± 0.071	0.947 ± 1.563	0.786 ± 0.150	0.073 ± 0.042	0.760	0.164	0.289
FFW I2C 20*μ*m	0.241 ± 0.178	0.273 ± 0.160	0.061 ± 0.061	0.789 ± 1.366	0.799 ± 0.146	0.068 ± 0.039	0.797	0.165	0.310
GAT I2C 20*μ*m	0.232 ± 0.168	0.257 ± 0.159	0.054 ± 0.060	0.790 ± 1.339	0.804 ± 0.149	0.067 ± 0.039	0.795	0.153	0.289

Pearson correlation (PCC), spearman correlation (SCC), mutual information (MI), mean squared error of log1p expression (MSE), cosine similarity (CosSim), Jenson-Shannon divergence (js_div), structural similarity index (SSIM), rand index adjusted for chance (ARI) and normalized mutual information between two clusterings (NMI) of merged cross-validation predictions of linear evaluation head of the ResNet visual feature backbone, FFW Image2Count (I2C), and graph-based Image2Count models to counts of the test data from the GeoMx dataset, from average per-molecule or per-cell/niche metrics. Standard deviation is specified over proteins (PCC, SCC, MI, MSE) and over niches (CosSim, js_div) where possible. Merged predictions were obtained by calculating the mean prediction over all cross-validation runs. Predictions were done on GeoMx test ROIs, consisting on average of 269.8 cells. Best performing model per metric is highlighted in bold.

#### Image2Count predicts cell specific expressions

**Fig. 5:**
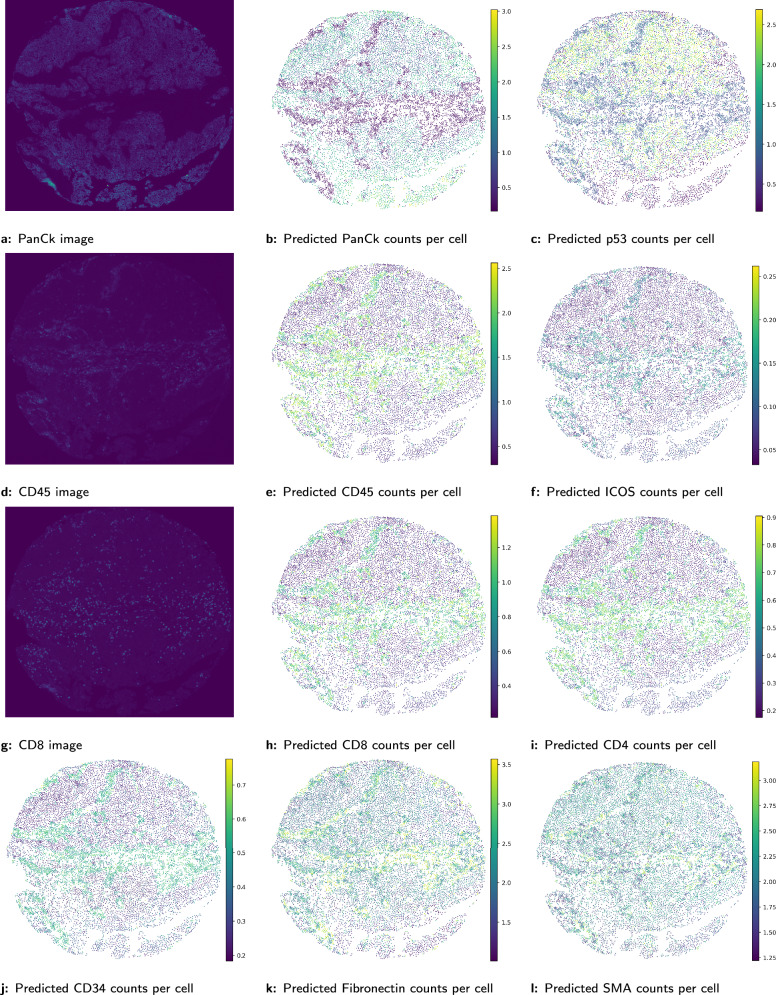
Visualization of predicted data in a full tissue core. Normalized merged FFW Image2Count predictions of cells (**b**, **c**, **e**, **f**, **h**–**l**) in a core on a test slide (010-1C54) of a clear cell ovarian tumor and image channels of selected biomarkers (**a**, **d**, **g**) from a representative test set image.

Predicted cell expressions were spatially mapped onto cell coordinates and compared to the visual markers (PanCk, CD45 and CD8 image channels) (Fig. 5) in a full core of a (clear cell) OC tumor from the test data. Predicted expressions of PanCk (Fig. 5a, b), CD45 (Fig. 5d, e) and CD8 (Fig. 5g, h) matched the true expression in the respective image channels. Cells that were strongly CD45 and CD8 positive also had a higher predicted expression of the respective marker, with neighboring cells also predicted to express CD45 and CD8 when segmented cells were in close proximity. For PanCk, a distinction between positive and negative cells was observed (Fig. 5b) for selected markers. Spatial mapping of predicted p53 expression (Fig. 5c) indicated a largely inverse correlation to PanCk expression, i.e., p53-positive tumor cells were PanCk-low, which could be expected as PanCk expression is known to be lost in less differentiated cells. Cells that were predicted to be highly positive for immune markers (Fig. 5e, f, h, i) displayed low scores for Fibronectin, indicating that the model had learned to distinguish between immune cells and fibroblasts. Cells predicted to express Fibronectin were also mainly located at the tumor boundary (Fig. 5k), which is the expected spatial localization of Fibronectin-positive cells^39^. The spatial mapping indicated that the model occasionally failed to discriminate between T-cell types. For example, in Fig. 5i, cells predicted to be positive for both CD4 and CD8 were observed.

**Fig. 6:**
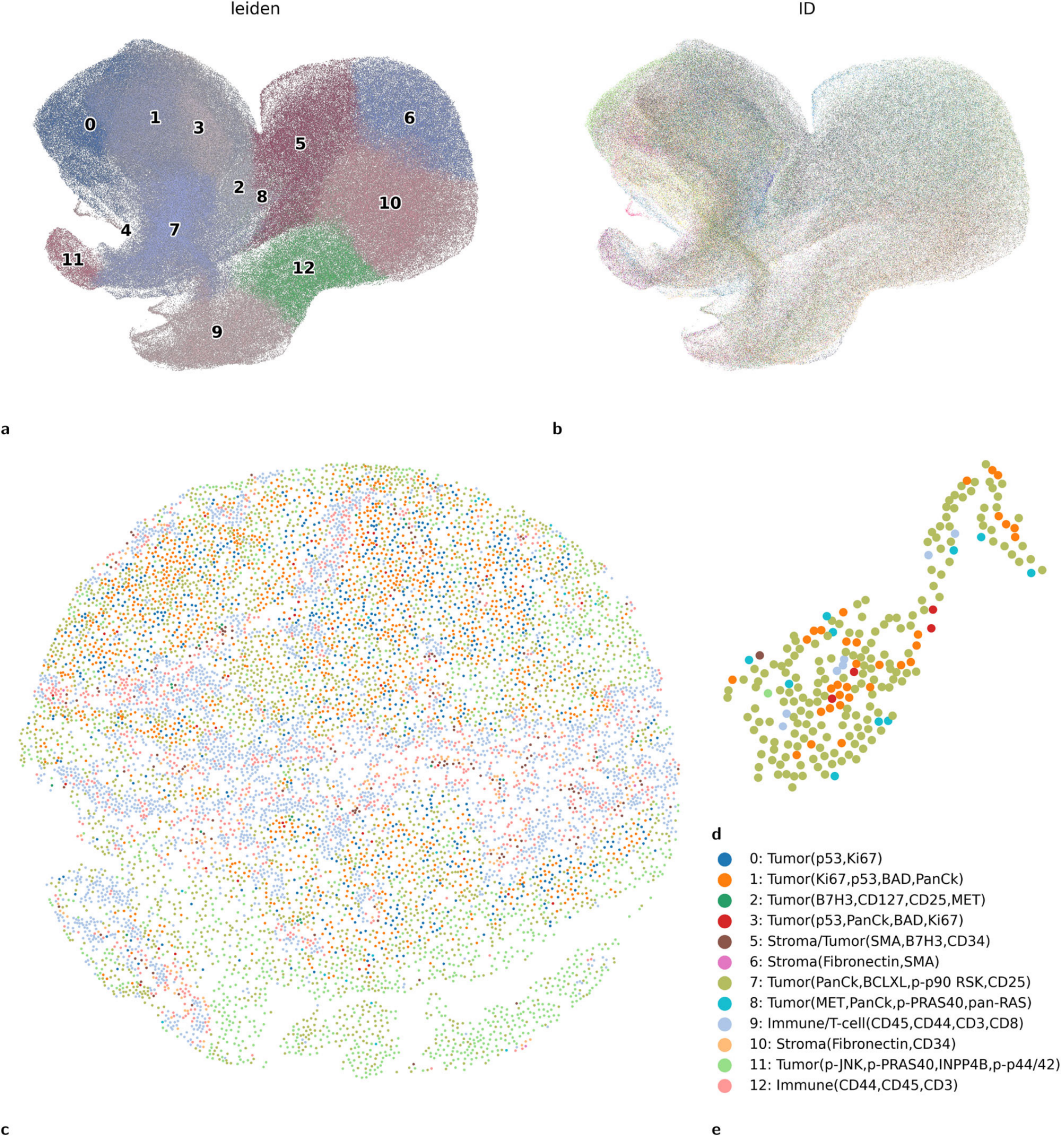
Single-cell analysis and spatial mapping of predicted clusters from GeoMx data. Clusters were constructed from predicted protein expression by graph model of test ROIs and test slide (1C54) cores from the GeoMx data. **a** UMAP shows separation of stroma (right), immune (bottom) and tumor (left) associated clusters. Clusters and their top ranked markers are: 0: Tumor (p53, Ki67), 1: Tumor (Ki67, p53, BAD, PanCk), 2: Tumor (B7H3, CD127, CD25, MET), 3: Tumor (p53, PanCk, BAD, Ki67), 4: Tumor/Immune (p53, CD44, Ki67, IDO1, CD45), 5: Stroma/Tumor (SMA, B7H3, CD34), 6: Stroma (Fibronectin, SMA), 7: Tumor (PanCk, BCLXL, p-p90 RSK, CD25), 8: Tumor (MET, PanCk, p-PRAS40, pan-RAS), 9: Immune/T-cell (CD45, CD44, CD3, CD8), 10: Stroma (Fibronectin, CD34), 11: Tumor (p-JNK, p-PRAS40, INPP4B, p-p44/42), 12: Immune (CD44, CD45, CD3). **b** No apparent clustering based on Patient ID was observed. **c** Predicted clusters mapped onto a full core on a test slide (010-1C54), and **d** Predicted clusters mapped onto ROI 019-1C54. **e** Coarse (tumor/stroma/immune) cluster annotation with top predicted markers per cluster for **c**, **d**.

We further investigated the predicted protein expression of cells from the test dataset and the 1C54 dataset (for which the full tissue cores had been used, thus not corresponding to GeoMx ROIs) through single-cell analysis with Leiden clustering (Fig. 6a). Of note, no clear distinction based on patient ID was observed (Fig. 6b). Cell clusters were visualized spatially for a full TMA core (Fig. 6c) and ROI 019-1C54 (Figs. 6d and S11). Wilcoxon rank-sum test-associated clusters showed a clear separation in the UMAP between stroma, immune and tumor clusters (Figs. 6a, S12 and S13). While cell-type-specific annotations down to individual phenotypes remained hard to define, we identified two immune clusters (cluster 9 and 12), as well as differentiation between different tumor cell clusters, which agreed with QuPath annotation (Fig. S14). When visualizing spatial cluster localization in the tissue of the clear cell ovarian tumor (Fig. 6c), cluster 9 was also clearly localized to tissue areas for which the model predicted CD8 expression to be high (Fig. 5h). Spatial cluster visualization further revealed spatial discrimination of tumor phenotypes, with stronger presence of cluster 1 (Ki67, p53, BAD, PanCk) in the top of the tissue core and cluster 7 (PanCk, BCLXL, p-p90 RSK, CD25) and 11 (p-JNK, p-PRAS40, INPP4B, p-p42/44) in the bottom, indicating that tumor heterogeneity can be largely captured by the model predictions. Similarly, most cluster 12 immune cells were localized in the stroma, in concordance with the corresponding CD45 and CD8 images (Fig. 5e, h).

## Discussion

In this study, we developed a graph-based model termed Image2Count to predict high-plex biomarker single-cell expression by learning from “bulk”-ROI GeoMx counts and 4-plex IF images. We used a publicly available t-CyCIF CRC dataset with “true” single-cell resolution staining of a large number of biomarkers, as well as a publicly available CosMx NSCLC dataset, and showed that prediction accuracy of expression from bulk data increased with cellular neighborhood size. This is specifically relevant when considering correlations between neighborhoods covering tens to hundreds of cells, as much larger neighborhoods might lose meaningful variation in expression. Assessment of Image2Count’s performance in CosMx and t-CyCIF data indicated that the model is best suited for regions containing roughly 8–43 cells to predict biologically meaningful information. Increased resolution, i.e., increasingly smaller regions of tissue or even single cells, showed lower correlation between predicted and true signal. Hence, future work will benefit from considering penalizing models for creating single-cell predictions which are far from observed single-cell expression, similar to what Fatemi et al. proposed for their model^17^. Single-cell expression data (spatial or otherwise) have to be available if such improvement is to be applied, which is not always given, as in the case of GeoMx data.

In general, no significant differences in performance were observed between the FFW and the GAT Image2Count model (Table S24), with only slightly lower prediction performance for the FFW model, for which neighborhood context was disregarded. We believe that the window size for cell cut-outs provides a plausible explanation for this indifference. Cut-outs are centered around a query cell but will in most cases also contain information from closely interacting cells. Hence, most of the relevant information for predicting a cell’s expression may already be contained in the given cell cut-out. The clearest difference in performance between FFW and GAT was observed for the CosMx dataset, indicating that spatial context might be more relevant for high-plex transcriptomic data. Cell cut-out width and height of 10–20 μm performed best for most marker predictions, with smaller cut-outs performing better for low-abundance markers like CD8a.

As expected, model access to visual features was beneficial, as demonstrated by overall high correlation for predictions of CD45, CD8a and Keratin in the t-CyCIF dataset. When testing performance on the t-CyCIF data using a visual feature backbone trained on only DNA and Keratin images, thus excluding immune markers CD45 and CD8, a sharp drop in performance was observed, especially for more low-abundance immune markers. In this study, the choice of visual markers was restricted to the IF panels that had been used for generating the different datasets. Feature selection for identifying marker combinations for optimal prediction of key spatial expression, for example from therapeutic targets and associated cells, could be performed using high-plex protein imaging such as t-CyCIF. Striving for maximal panel condensation with retained prediction power will be important to reduce cost and complexity for staining in the clinical setting.

We also highlight the importance of accurate bulk counts. For CosMx data, Image2Count achieved correlation metrics comparable with reported performance of other models trained on H&E and Visium data^13^. Dropout events, which are common in single-cell transcriptomics data due to low mRNA amounts or insufficient capture, were shown to impact the model’s ability to learn true cell expression, but also downstream analysis of model performance. Actual model performance might be better than currently calculated from raw data, as was indicated through the improved single-cell expression correlation when using imputation to account for dropouts in CosMx data. In the case of the t-CyCIF dataset, high Spearman correlation values (>0.64) were achieved for areas consisting of more than 7.8 cells (see Table S2). Correlation between predicted bulk counts and actual bulk counts of ROIs was substantially lower for the GeoMx dataset, with an average Spearman correlation of 0.27 compared to 0.79 for the t-CyCIF dataset or 0.44 for CosMx on 8-hop subgraphs. This may be due to the significantly higher number of patients in the GeoMx dataset (*n* = 254) compared to the t-CyCIF (*n* = 16) and CosMx (*n* = 5). The GeoMx data was also based on a highly heterogeneous, population-based cohort of mixed OC histotypes, from which only small tissue regions (an average of 2-3 ROIs per tumor) had been collected. Such highly variable patient populations may require more data for model training. ROI collection was also predefined and included mainly tumor (PanCk-positive) regions, while random tissue areas were learned from the t-CyCIF and CosMx data. Selecting more diverse areas for GeoMx ROIs might increase performance, as more variable patterns and mix of phenotypes would be learned, and bleed-over across ROI boundaries could be combated. The impact of the downstream single-cell segmentation quality was not investigated but is also expected to be a limiting factor. Utilizing patch-based predictions (as described in ref. ^13^), which afterwards can be segmented for single-cell expression, might increase spatial specificity of predictions in future work.

When using larger than 20 μm cut-outs, the model showed a tendency of overfitting when training the visual feature backbone on the GeoMx dataset. This can be explained by larger regions leading to lower variability in the image data (and a more average expression of count data) when training on too few cells. In contrast, for the t-CyCIF and CosMx data, the visual feature backbone was trained on a larger total number of cells (160,000 cells for GeoMx; 8.6 million cells for t-CyCIF; 800,000 cells for CosMx), increasing the performance of the visual feature backbone and subsequent count prediction.

We highlight several findings that future work can benefit from: Predicting expression of single cells from training on data containing expression of more than 250 cells can work well. However, our models learned to predict not just single-cell expression, but rather the expression of the single-cell cut-out. Merging model predictions from a cross-validation run to build a consensus increased average single-cell prediction accuracy. Oversampling low-abundance cell phenotypes when learning visual representations also increased performance. Spatial context increased performance marginally when visual information for CD45 and CD8 was given. The inclusion of immune-specific markers improved predictions overall. While foundation models for simple H&E staining are available^40–43^, Gallagher-Syed et. al showed that the addition of other markers leads to misidentifying features, performing similarly to models pre-trained on ImageNet (a non-H&E image dataset)^44^. Hence, one of the challenges ahead is establishing a foundation model that can handle more diverse tissue staining, improving the visual feature backbone. Such a foundation model would also enable defining which condensed imaging panels to use for optimal prediction of cell expression and differentiation between visually similar cell types.

The ability of Image2Count to identify tumor-, immune-, and stromal cell-specific markers, and pathways and cell types for which no visual information was provided indicates that underlying biological patterns were learned, which is important for future applications of stratification based on TIMEs. In conclusion, we demonstrate that deep learning-based image analysis can leverage high-plex spatial omics data generated in research labs to provide detailed predicted tumor profiles from low-plex images from standard pathology specimens. Image2Count is currently most useful in research labs that have large cohorts stained for the same markers, and spatial omics data for a subsection of the cohort. Image2Count can then be applied to predict single-cell spatial omics data, with researchers assessing performance per gene, pathway, or cell type to identify niches for which Image2Count predictions can be trusted in the rest of their cohort. However, with current developments in computer vision and spatio-molecular analyzes, models such as Image2Count have great potential to translate to the clinical setting, to enhance personalized medicine by aiding in guiding immunotherapy and other targeted treatments based on TIME characteristics. To this end, the field would greatly benefit from a channel-invariant foundation model trained on a large variety of data to extract image features, as one of the current limitations is the lack of spatial omics data from large patient populations, and the difference in image data types.

## Methods

### GeoMx digital spatial profiling data acquisition

Ovarian Cancer (OC) patient tissue biopsies^45^ were analyzed using the GeoMx Digital Spatial Profiling platform (Nanostring)^24^. Ten tissue microarray (TMA) slides, with 4 μm thick cuts of 1 mm diameter formalin-fixed paraffin-embedded tissue cores (3 cores per tumor), were stained with SYTO 13 (nuclear stain) and three fluorescence- labeled antibodies against PanCK (tumor), CD45 (immune cells) and CD8 (cytotoxic T-cells), and panels of photocleavable oligonucleotide-tagged antibodies, as described^38^. ROIs were selected using the IF images, probes were collected by exposing ROIs to UV light, followed by quantification using nCounter (Nanostring). The protein panel consisted of 78 antibodies specific for immune and tumor markers (Fig. S15), including 6 control markers not included in our predictions. Based on the images of tumor cores from 254 patients, 636 ROIs were selected from representative tumor, stroma or mixed regions. Normalization of ROI protein counts was performed in the GeoMx analysis suite through linear scaling of protein counts based on the geometric mean of housekeeping protein Histone H3. OME-TIFFs were exported from the GeoMx analysis suite and cell segmentation was performed in QuPath with stardist (dsb2018_heavy_augment.pb)^46,47^.

### t-CyCIF data acquisition

The publicly available CRC Atlas 2022^27,28^ (obtained from the author`s github https://github.com/labsyspharm/CRC_atlas_2022) consists of data and images from 17 colorectal cancer (CRC) patients. The samples have been stained using cyclic immunofluorescence (t-CyCIF), generating single-cell resolution stainings of multiple (*n* = 35) protein markers. DNA staining (Hoechst) was included in each staining cycle; however, we only used the DNA channel from cycle 1. We did not include information from the isotype (negative) control markers. We used data from patients 2–17, consisting of 16 25-plex images, with corresponding spatial data, containing coordinates and marker abundance for each cell. Pseudo-bulk count data were constructed by adding expression based on marker abundance of all cells in a given area to resemble GeoMx data and train model predictions. Note that these visually obtained counts were high (high thousands per protein per cell) compared to GeoMx data (hundreds of protein counts for hundreds of cells together), as they were based on relative fluorescence units, which range between 0 and 65536.

### CosMx NSCLC data acquistion

The publicly available CosMx NSCLC data^23^ (available from Nanostring’s website) consist of CosMx data from eight tissue samples collected from five patients. Tissue had been stained for membrane, PanCK, CD45, CD3 and DAPI, and count data were from a 960-plex CosMx RNA panel. We preprocessed downloaded data into a format compatible with our code, as documented on our github. Dropout of gene counts is a known issue for some spatial omics methods such as CosMx^11^. We tentatively imputed missing transcripts to investigate how well Image2Count trained on non-imputed data reflects imputed cell counts, using a DCA^36^ with the following parameters: ae_type was set to zinb_condisp, lr to 0.0005, batch_size to 256 and hidden_size to (256, 128, 64, 128, 256). DCA is an established method for imputing missing counts for single-cell RNA-seq data.

### Preprocessing

For the t-CyCIF dataset, cell positions and per-cell mean staining intensities had been made available via the tool MCMICRO^48^ for all images^27^. Bottom and top 1% of summed cell expression were filtered to remove outliers. Each cell was then cut out in 50 × 50 (34 × 34 pixels), 20 × 20 (14 × 14 pixels) and 8 × 8 (6 × 6 pixels) μm windows, for different model runs, respectively, for the t-CyCIF dataset. For the GeoMx dataset, cells were cut out in 50 × 50 (124 × 124 pixels), 30 × 30 (74 × 74 pixels), 20 × 20 (50 × 50 pixels), 12 × 12 (30 × 30 pixels) and 8 × 8 (20 × 20 pixels) μm windows, respectively. We do not report on 50 × 50 and 30 × 30 *μ* m windows as the visual feature encoder overfitted given the GeoMx dataset size (Figure S16). For the CosMx dataset, cells were cut out in 18 × 18 (100 × 100 pixel) μm window. For all training data, channel-specific mean and standard deviation were calculated for normalization purposes. For CosMx 4 (total 732), for t-CyCIF 49 (686 total) equally distributed subgraphs, with an 11-hop neighborhood starting from the central cell (containing on average 384 and 260 cells, respectively), per WSI/patient/fov (field of view) were created for model training. When investigating accuracy for differently sized regions on the test data, 36 subgraphs per fov for CosMx were created (total 1800), 49 per WSI for CRC (total 2700). Region size was varied to 1, 2, 3, 5, 8 or 11 hop subgraphs.

### Visual feature extraction

Visual features were extracted via pre-training a ResNet model with the contrastive loss from SimCLR^26,49^, following similar approaches that extract visual features from multiplex IF images^7^. ResNet was chosen as an encoder as cell cut-outs consist of low pixel window sizes (from 100 × 100 to 6 × 6 pixels). Datasets with cell cut-outs below 50 × 50 pixels utilized ResNet18, while datasets with cell cut-outs above 50 × 50 pixels and a sufficient number of cells to train on used ResNet50 as an encoder.

The architecture of the ResNet model had to be modified to allow for training with a contrastive loss to work on small images and to handle image data with more or less channels than three. We therefore modified the first Conv2d layer of a ResNet model obtained via PyTorch to input the number of channels we provided (four if not otherwise specified) and changed the kernel size to (3, 3) and stride to 1, as motivated by ref. ^26^ when working on smaller images. We also followed the recommendation to remove the first max-pooling operation. We did not use these changes when training on images with 74 × 74 pixels or larger. The projection head *g*(.) was a nonlinear MLP with one hidden layer. However, unlike in the SimCLR paper *g* did not work on the avgpooling layer output, but followed the implementation of NaroNet and operated on a nonlinear embedding *e*(.), which does follow the avgpooling layer. This allows for visual features to exist in a smaller feature space *e*, needed for downstream training and tasks, while performing the contrastive loss on outputs of *g*, which is conjectured to lose relevant information as *g* is trained to be more invariant to data transformations^26^.

Stochastic Gradient Descent was utilized as the optimizer with a weight decay of 1*e* − 6 (5*e* − 6 for the GeoMx dataset when training on images of size 50 × 50 μm or larger to combat overfitting), momentum set to 0.9, as well as Nvidia’s apex PyTorch implementation of LARC, following SimCLR^26,50,51^. Learning rate scheduling was performed with PyTorch’s OneCycleLR^52^. OneCycleLR scales the learning rate to the given maximum learning rate in a specified fraction of total training epochs, and then decreases it. Updates to the learning rate are performed for every minibatch.

Image augmentation consisted of random cropping and resizing into the original shape, horizontal and vertical flipping, introduction of random artifacts, random background, Gaussian blurring, Gaussian noise and z-score normalization.

During training of the visual feature extraction module, images of cells that were underrepresented in the data were over-sampled to improve features extracted from low-abundance cell types. This was done by first extracting centroid pixels of every image and then performing K-Means elbow on a random subset of 10,000 pixels 5 times to obtain the optimal number of clusters. The optimal number was then used to run K-Means on all image pixel centroids, sampling from every cluster equally during training.

Visual representations of cells for downstream tasks were obtained from *e*(.) after pre-training the visual feature extraction module once, for all cells in a given training dataset, without further fine-tuning.

### Graph learning

Learned cell representations were transformed into a cell graph with squidpy^53^ by calculating the Euclidean distance to each cell’s 6 nearest neighbors. AdamW^54^ with a weight decay was utilized to optimize model parameters in this part of the model. Visual representations obtained from the previous step were embedded via a 2-layer MLP, as shown in Fig. 1c in the embedding block. *i* FFW blocks, built of pre-LayerNorm and ReLU, followed by a two-layer Feed-Forward Network (FFW) and residual connection, further extracted relevant features from the image embeddings. What follows is *n* GAT^55,56^ blocks when utilizing GAT Image2Count instead of FFW Image2Count models (see GATv2 block in 1c), consisting of pre-normalization and activation, shown to be beneficial for deep networks^57,58^, graph attention convolution v2 (utilizing torch geometric) with *n*_*heads*_ attention heads, which get concatenated, and afterwards transformed into the original embedding dimension via a linear layer, followed by a residual connection and a FFW Block. Li et al. showed that deep graph convolutional networks benefit, similarly to convolutional neural networks (CNNs), from residual connections^59^. We therefore incorporated this concept to facilitate the construction of deep models. The resulting GAT blocks are highly similar to modern transformer blocks.

The last part of Image2Count prediction heads consisted of projecting the learned cell representation via a 2-layer MLP onto expression counts, then calculating the exponential of expression counts and clipping values below 1*e*-5 and above 1*e*+6 (similar to the mean activation used by Eraslan et al.^36^). Downstream analysis used this layer as output to obtain single-cell-level expression predictions. While training, all cell expressions of a graph were summed together via a global add pool, which was used to calculate the L1 loss between predicted expressions and the observed bulk counts of the region/graph after calculating their log + 1 values to achieve more stable learning. We also used 1 − CosineSimilarity(*x*, *y*) as an additional loss term, as can be seen in Equation 1. Values used for *γ*_1_ and *γ*_2_ in Equation 1 were always 1.

1 $$\begin{array}{rcl}{L}_{i}&=&{\gamma }_{1}* L1(\log ({x}_{i}+1),\log ({y}_{1}+1))\\ &&+{\gamma }_{2}* (1-\,{\mathrm{sim}}\,({x}_{i},{y}_{i}))\end{array}$$

Graphs were constructed via KNN, with each cell having edges to its six nearest neighbors. Edge attributes consisted of the distance between cells and node attributes were the visual feature representations. While training, we made use of multiple graph augmentations: Positional jittering, where cell positions are randomly jittered locally in pixel space and recomputing edges and distances without changing the macro topology; as well as randomly dropping edges and nodes from the graph. Data utilized the same training and test splits used for the visual feature extraction, with training data being further split over patients/slides for cross-validation. Training was stopped when no improvement in loss was observed on validation folds. We compared GAT Image2Count to FFW Image2Count and a linear prediction model. Model parameters were chosen based on coarse manual testing of model performance on training data.

### Linear prediction

Linear prediction of gene expression was done through a simple one-layer linear network *f*^*R*×*num*_*genes*^. Batch size of 64, learning rate of 0.005, weight decay of 5*e* − 4, AdamW as optimizer, node dropout of 0.05, early stopping of 25, and tenfold cross-validation were used as parameters for the t-CyCIF dataset. For the GeoMx dataset, the learning rate was adjusted to 0.01; for the CosMx dataset, sixfold cross-validation was used.

### CosMx NSCLC Image2Count model

Visual representation learning was performed using all image channels. Cell cut-outs were clustered with K-Means (*k* = 30) to oversample low-abundance cell types. As base encoder a ResNet50 model was chosen because of the cell cut-out size (18 × 18 μ with 100 × 100 pixels), with batch size of 512, max learning rate of 0.1, 30 warm-up epochs, 300 total epochs, embedding size of 512, loss dimension of 128, data augmentation with default parameters and an additional brightness and contrast augmentation in 80% by randomly adjusting brightness and contrast between 0.5 and 2 for all channels. Lung 6 and Lung 13 were used for test data.

GAT Image2Count prediction head was constructed as follows: 3 linear blocks, 3 GAT blocks, GATv2 node size of 64, 8 attention heads and positional cell jittering of 40. The FFW Image2Count prediction head consisted of 17 linear blocks. Both used batch size of 64, learning rate of 0.0001, an embed node size of 128, weight decay of 5*e* − 3, early stopping of 25, sixfold cross-validation over slides and an embedding dropout of 0.5. Consensus predictions of the test data were obtained by calculating the mean test prediction over all 6 Image2Count prediction heads.

### t-CyCIF CRC Image2Count model

To resemble the GeoMx data, visual representation learning on the t-CyCIF data was done using image channels 0 (Hoechst1), 10 (Keratin), 14 (CD45), and 19 (CD8a). Visual representation learning was also performed on only channel 0 (Hoechst1) and 10 (Keratin) to investigate the benefits of cell type-specific staining for the downstream task. In order to oversample low-abundance clusters during training for a more equal cell class distribution, centroid pixels of each cell cut-out were used for K-Means, with *k* = 30 for 4-channel and *k* = 10 for the 2-channel model determined as a good fit number of clusters via the elbow method. ResNet18 was chosen as the encoder as the cell cut-out pixel size was less then 50 × 50, with a batch size of 4096, a max learning rate of 0.1, 10 warm-up epochs and running for 100 epochs. Embedding size of *e*() was set to 32, while the loss operating on outputs of *g*() had 16 dimensions. Data augmentation was done with our default parameters. We split the 8.6 million cells into 80% for training and 20% for testing by using WSI 3, 5, and 13 for testing and the rest for training, reaching a test loss accuracy after training of 71.16%, while having a performance of 95.06% on the training set with 50 μm cut-outs. We used the following parameters to construct Image2Count prediction heads: GAT Image2Count was done with 24 linear blocks, 6 GAT blocks, GATv2 node size of 32, 8 attention heads, edge dropout of 0.3, and positional cell jittering of 40. FFW Image2Count consisted of 35 linear blocks. Both Image2Count models used a batch size of 64, a learning rate of 0.0001, an embedded node size of 128, weight decay of 5*e* − 4, early stopping of 25, tenfold cross-validation over WSI, and embedding dropout of 0.1. The previous train/test split was utilized, performing tenfold cross-validation on the training data (split over patients) and comparing predictions on the test data. We compared our GAT Image2Count model to a FFW Image2Count and a linear prediction head for the frozen visual feature backbone (see section Graph Learning), when training the visual representation backbone with two channels (DNA and Keratin) and four channels (DNA, Keratin, CD45, CD8a), as well as cell cut-out sizes of 8 × 8 and 20 × 20 μm windows instead of 50 × 50 μm. Consensus or merged model predictions of the test data were obtained by taking the mean test prediction of all 10 cross-validation Image2Count prediction heads.

### GeoMx OC Image2Count model

A ResNet18 was chosen as an encoder, as cell cut-out size in pixels was below 50 × 50, with a batch size of 1024, max learning rate of 0.1, 95 warm-up epochs and training for 950 epochs. As the GeoMx dataset was smaller than the t-CyCIF dataset, we had to reduce the batch size and increase the number of epochs to achieve similar performance. The embedding dimension of layer *e*() was set to 32, and the output dimension of *g*(), upon which the contrastive loss was calculated, was set to 16. Data augmentation was performed using default parameters. The centroid pixel of each cell cut-out was used for K-Means, with *k* = 20 determined as a good fit number of clusters via the elbow method, to oversample low-abundance clusters during training for a more equal cell class distribution. The ten tumor TMAs were split into a train and test set: seven for training (486 ROIs), three for testing (150 ROIs). The contrastive loss accuracy for the test data was 0.67 after training, and on the training data, 0.88.

Larger areas on slide 1C54, part of the test set, for which no count data was available over the whole area, were segmented via QuPath and Stardist to create single-cell expression predictions from the trained model for inference.

We used the following parameters to construct Image2Count prediction heads: GAT Image2Count used 3 linear blocks, 3 GAT blocks, GATv2 node size of 8, 2 attention heads, edge dropout of 0.3, positional cell jittering of 40. FFW Image2Count consisted of 7 linear blocks. Both Image2Count prediction head models used a batch size of 64, a learning rate of 0.0005, an embedded node size of 32, weight decay of 1*e* − 2, early stopping of 50, tenfold cross-validation over patients on the training data and embedding dropout of 0.5. Merged model predictions of the test data were obtained by taking the mean test prediction of all cross-validation Image2Count prediction heads. Coarse manual tests confirmed parameters to be a good fit.

### Single-cell analysis

After training the FFW/GAT Image2Count prediction head models and linear prediction head for the frozen ResNet, we generated single-cell expression predictions of the test data and performed single-cell analysis. Consensus or merged model predictions of the test data were obtained by taking the mean prediction of all cross-validation models trained on the training data. Single-cell analysis was performed with scanpy^37^. Count normalization was performed through median count depth normalization followed by a log1p transformation and scaling to unit variance with a mean of zero. Principal Component Analysis (PCA) was then performed using markers whose prediction over all test ROIs had Spearman *p*-values below 0.05, retaining a maximum of *min*(*number*_*of*_*genes*, 100) components (for the CRC dataset, we also excluded Hoechst, NaKapTase, CDX2 and Ki67_570). Subsequently, the 10 nearest neighbors were determined based on all principal components. Using the nearest neighbors graph, a Uniform Manifold Approximation and Projection (UMAP) was computed to embed the graph into a two-dimensional space for visualization. Cells were assigned to clusters using Leiden clustering, with a resolution of 0.5 for GeoMx and 0.25 for CRC. Proteins associated with clusters were identified using the Wilcoxon rank-sum test.

To visualize predicted expression of cells and their location in the tissue, we plotted the per-cell count normalized and log1p transformed predicted and normalized original cell signals on top of the corresponding cells in an image or image section with squidpy^53^, generating a clearer visualization of the predicted and actual spatial distribution of protein expressions.

### Comparison metrics

Metrics were calculated on the test data. For CosMx and t-CyCIF datasets, ground truth data was available; therefore, metrics were calculated on the single-cell level and at different neighborhood sizes as described in Preprocessing. Pearson correlation is a linear correlation. Spearman correlation is rank-based, indicating capability to rank differences correctly. MI measures dependency between two variables, with higher values indicating higher dependency. MSE of log1p values is the mean squared distance, indicating how close predictions are to observed values. Cosine Similarity measures by how much two vectors point in the same direction, or in our case, how similar gene expression is in two cells, ranging from −1 (opposite direction) to 1 (identical direction). Jensen–Shannon divergence measures how similar two distributions (cell gene expression) are, with lower values indicating higher similarity. SSIM is a metric frequently used in image processing, calculating how structurally similar two images are, or in our case, the gene expression of two identically numbered sets of cells (ranging from 0 to 1, with 1 indicating higher similarity). ARI is a measurement indicating how similar two sets of clusterings are, adjusted for chance, ranging from 0 (no similarity) to 1 (perfect correlation). NMI similarly measures how well two clusterings fit one another by calculating the normalized mutual information, ranging from 0 (no mutual information) to 1 (perfect correlation). Jensen–Shannon divergence, Pearson and Spearman and correlation were calculated through scipy on the marker level; MSE of log1p counts and cosine similarity were calculated through torch; MI, ARI and NMI were calculated through sklearn; and SSIM was calculated through skimage implementation. For ARI and NMI, clusters of the predicted and true count data were obtained by following the clustering procedure described in Single-cell analysis, with the Leiden parameter resolution set to 0.5. For SSIM, count data was normalized by log1p transformation with scanpy, the data_range parameter for normalized predicted *X* and true counts *Y* was set to $\max (\max (X),\max (Y))-\min (\min (X),\min Y)$, and channel_axis to None.

### Enrichment analysis

Enrichment analysis was performed on the true and predicted single-cell or k-hop neighborhood test data of the CosMx dataset. Count data was normalized through median count depth normalization followed by a log1p transformation with scanpy. Clusters were obtained separately for true and predicted count data as described in Single-cell analysis, with the Leiden resolution parameter set to 0.5. We utilized decoupler^60^ to obtain transcription factor (CollecTRI^29^) and pathway (PROGENy^30^, hallmark^31^, Reactome^32^ and KEGG^33^) genesets. Reactome and KEGG gene sets were filtered for gene sets containing 15–500 genes. Gene set enrichments were calculated on a single-cell or k-hop neighborhood basis through ULM (univariate linear model). ULM was chosen as a method as it performed computationally efficiently and compares well to other methods^60^. ULM parameter tmin was always set to 15, ensuring that at least 15 genes of any geneset were in the CosMx dataset. After filtering 222 transcription factors for ColecTRI, 13 pathways for PROGENy, 30 pathways for hallmark, 106 pathways for Reactome and 48 pathways for KEGG remained. Following ULM enrichment, we utilized *dc*. *tl*. *rankby*_*group* to rank the top 5 transcription factors or pathways per cluster, comparing to the rest, and setting the parameter method to *t*-*test*_*overestim*_*var*. All pathways with a score below 0 and and Benjamini–Hochberg adjusted *p*-value below 0.05 were discarded.

As we were interested in quantifying how much Image2Count predicted counts identify the same pathways per cluster as the true counts, we created a symmetric (in respect to predicted and true count clustering) cluster enrichment coverage metric. Let *K* be the set of all clusters, *P*_*i*_ the ranked list of predicted pathways for cluster *i*, *T*_*i*_ the ranked list of true pathways for cluster *i* and top_*k*_(⋅) the first *k* elements of a ranked list.

2 $$\,\mathrm{Cov}(P,T,k)=\frac{{\sum }_{i\in K}| {\mathrm{top}}_{k}({P}_{i})\cap {\mathrm{top}}_{k}({T}_{i})| }{{\sum }_{i\in K}| {\mathrm{top}}_{k}({T}_{i})| }$$

Given cluster *K*_*x*_ of predicted counts *X* and cluster *K*_*y*_ of predicted counts *Y*, we obtain ranked lists per cluster of predicted pathways ${P}_{{K}_{x}}$, ${P}_{{K}_{y}}$ and ranked list per cluster of true pathways ${T}_{{K}_{x}}$, ${T}_{{K}_{y}}$.

3 $$\,{\mathrm{SymCov}}({P}_{{K}_{x}},{P}_{{K}_{y}},{T}_{{K}_{x}},{T}_{{K}_{y}},k)=\frac{{\mathrm{Cov}}({P}_{{K}_{x}},{T}_{{K}_{x}},k)+{\mathrm{Cov}}\,({P}_{{K}_{y}},{T}_{{K}_{y}},k)}{2}$$

We obtained lung celltype genesets from CellMarker2.0^34^ (selecting species human, tissue Lung/Lung and cell types all) to identify how well Image2Count can recapitulate cellular identities from known signatures. Duplicated entries were removed and the decoupler ULM parameter tmin set to 5. After filtering, 44 cell types remained. Analysis was performed only on single cells, and top 5, top 3 and 1 symmetric coverage were calculated after otherwise following the same process as described for the pathway coverage analysis.

### Statistical testing of model performance

Statistical tests were performed to identify difference in model performance between three model architectures (ResNet, FFW, GAT). Metrics that could be calculated per gene (PCC, SCC, MI, MSE) or per cell/niche (CosSim, Jensen–Shannon divergence) at single-cell and different niche size resolutions were assessed via statsmodels mixedlm. To have uniformly higher values representing better performance, MSE and Jensen–Shannon divergence metrics were multiplied by −1. Gene expression (and therefore performance) can be highly correlated as they belong to similar pathways. We collected per gene in the CosMx dataset CollecTRI transcription factors, PROGENy, Hallmark, Reactome and KEGG pathways, filtering out all pathways which contained fewer than 10 genes in the CosMx dataset. Based on pathways/transcription factors per gene, we calculated the Jaccard distance between all genes via scipy. We then performed agglomerative clustering on the Jaccard distance matrix through sklearn, setting the number of clusters to 50, metric to precomputed and linkage to complete. Obtained clusters per gene were then used as groups for the mixedlm model. For cell/niche metrics, we obtained the single-cell clusters of the ground truth data and used those as the groups for mixedlm. The mixedlm formula is set to {metric} ~ architecture. *P*-values of *t*-tests were FDR corrected via Benjamini–Hochberg through scipy. The results of merged models on the CosMx test data is displayed in Table S24.

## Supplementary information

Supplementary Information

## Data Availability

The CosMx NSCLC data are available from the original source. The t-CyCIF CRC 2022 Atlas is available from the original authors' GitHub. The in-house GeoMx OC dataset is available in Zenodo, at DOI: 10.5281/zenodo.17395924. Images can be made available upon request to the corresponding author.

## Code availability

Code is available on github.
