# Supplementary material for: Prediction of single cell expression from low-plex immunofluorescence images for guiding precision oncology
Source: NPJ Precis Oncol. 2026 Jul 25;10:287. doi: 10.1038/s41698-026-01601-z (PMC13401605; doi:10.1038/s41698-026-01601-z)
Supplement: Supplementary file 1 — Supplementary Information [file 41698_2026_1601_MOESM1_ESM.pdf]

Supplementary

	Single Cell	1 hop	2 hop	3 hop	5 hop	8 hop	train size
N cells CosMx	1	7.68	21.28	42.95	109.69	266.01	483.71
ResNet	0.055 ± 0.030	0.109 ± 0.056	0.145 ± 0.068	0.148 ± 0.079	0.172 ± 0.088	0.230 ± 0.087	0.281 ± 0.085
ResNet merged	0.067	0.131	0.169	0.174	0.198	0.258	0.310
FFW I2C	0.063 ± 0.023	0.129 ± 0.044	0.180 ± 0.052	0.200 ± 0.064	0.253 ± 0.066	0.321 ± 0.062	0.370 ± 0.059
FFW I2C merged	0.094	0.183	0.246	0.276	0.335	0.401	0.450
GAT I2C	0.067 ± 0.028	0.135 ± 0.045	0.189 ± 0.049	0.213 ± 0.057	0.275 ± 0.049	0.349 ± 0.036	0.401 ± 0.030
GAT I2C merged	0.097	0.185	0.250	0.283	0.345	0.413	0.462
ResNet DCA	0.103 ± 0.070	0.172 ± 0.095	0.201 ± 0.096	0.191 ± 0.100	0.207 ± 0.100	0.268 ± 0.091	0.320 ± 0.086
ResNet DCA merged	0.128	0.205	0.235	0.222	0.237	0.299	0.352
FFW I2C DCA	0.137 ± 0.058	0.215 ± 0.073	0.256 ± 0.072	0.261 ± 0.079	0.303 ± 0.072	0.367 ± 0.063	0.414 ± 0.059
FFW I2C DCA merged	0.213	0.310	0.351	0.360	0.399	0.455	0.499
GAT I2C DCA	0.146 ± 0.070	0.228 ± 0.074	0.274 ± 0.066	0.284 ± 0.068	0.334 ± 0.050	0.403 ± 0.031	0.452 ± 0.025
GAT I2C DCA merged	0.221	0.316	0.361	0.374	0.416	0.473	0.516
N cells CycIF	1	7.78	19.85	38.25	95.88	234.84	438.54
ResNet	0.211 ± 0.018	0.324 ± 0.015	0.391 ± 0.016	0.447 ± 0.015	0.509 ± 0.014	0.553 ± 0.014	0.580 ± 0.013
ResNet merged	0.221	0.339	0.406	0.463	0.524	0.568	0.594
FFW I2C	0.370 ± 0.005	0.499 ± 0.008	0.547 ± 0.009	0.589 ± 0.010	0.637 ± 0.010	0.665 ± 0.010	0.682 ± 0.010
FFW I2C merged	0.416	0.538	0.581	0.620	0.665	0.689	0.705
GAT I2C	0.371 ± 0.016	0.498 ± 0.015	0.548 ± 0.014	0.591 ± 0.014	0.639 ± 0.013	0.668 ± 0.014	0.685 ± 0.014
GAT I2C merged	0.423	0.540	0.584	0.623	0.668	0.693	0.709
ResNet 2 Channels	0.147 ± 0.009	0.251 ± 0.008	0.311 ± 0.010	0.371 ± 0.011	0.441 ± 0.011	0.483 ± 0.012	0.511 ± 0.012
ResNet 2 Channels merged	0.155	0.262	0.323	0.385	0.455	0.498	0.525
FFW I2C 2 Channels	0.279 ± 0.010	0.391 ± 0.011	0.445 ± 0.012	0.496 ± 0.012	0.554 ± 0.012	0.588 ± 0.012	0.608 ± 0.012
FFW I2C 2 Channels merged	0.322	0.427	0.476	0.523	0.578	0.610	0.628
GAT I2C 2 Channels	0.287 ± 0.006	0.384 ± 0.006	0.437 ± 0.006	0.487 ± 0.006	0.546 ± 0.007	0.581 ± 0.009	0.601 ± 0.009
GAT I2C 2 Channels merged	0.335	0.428	0.476	0.525	0.580	0.614	0.633
ResNet no KM	0.153 ± 0.004	0.264 ± 0.008	0.322 ± 0.010	0.380 ± 0.010	0.449 ± 0.011	0.501 ± 0.010	0.527 ± 0.009
ResNet no KM merged	0.161	0.277	0.336	0.393	0.462	0.514	0.539
FFW I2C no KM	0.225 ± 0.019	0.379 ± 0.018	0.449 ± 0.018	0.506 ± 0.018	0.569 ± 0.017	0.606 ± 0.016	0.627 ± 0.015
FFW I2C no KM merged	0.343	0.480	0.532	0.577	0.626	0.654	0.671
GAT I2C no KM	0.213 ± 0.017	0.391 ± 0.015	0.465 ± 0.013	0.523 ± 0.013	0.584 ± 0.013	0.620 ± 0.013	0.641 ± 0.013
GAT I2C no KM merged	0.331	0.478	0.531	0.576	0.625	0.653	0.670
ResNet 8µm	0.225 ± 0.011	0.370 ± 0.010	0.449 ± 0.009	0.513 ± 0.007	0.580 ± 0.006	0.620 ± 0.006	0.644 ± 0.006
ResNet 8µm merged	0.246	0.389	0.465	0.527	0.591	0.630	0.653
FFW I2C 8µm	0.326 ± 0.024	0.451 ± 0.021	0.503 ± 0.020	0.548 ± 0.018	0.599 ± 0.017	0.630 ± 0.016	0.648 ± 0.016
FFW I2C 8µm merged	0.378	0.489	0.533	0.574	0.620	0.648	0.665
GAT I2C 8µm	0.323 ± 0.028	0.446 ± 0.023	0.500 ± 0.022	0.545 ± 0.020	0.597 ± 0.019	0.628 ± 0.017	0.646 ± 0.017
GAT I2C 8µm merged	0.387	0.489	0.534	0.574	0.621	0.649	0.666
ResNet 20µm	0.247 ± 0.033	0.366 ± 0.032	0.430 ± 0.031	0.479 ± 0.031	0.539 ± 0.030	0.577 ± 0.028	0.601 ± 0.027
ResNet 20µm merged	0.281	0.397	0.460	0.507	0.565	0.601	0.623
FFW I2C 20µm	0.374 ± 0.010	0.484 ± 0.009	0.527 ± 0.010	0.568 ± 0.009	0.616 ± 0.008	0.645 ± 0.008	0.663 ± 0.009
FFW I2C 20µm merged	0.412	0.512	0.550	0.588	0.633	0.659	0.676
GAT I2C 20µm	0.362 ± 0.020	0.473 ± 0.014	0.522 ± 0.014	0.564 ± 0.015	0.614 ± 0.015	0.644 ± 0.015	0.661 ± 0.015
GAT I2C 20µm merged	0.420	0.513	0.554	0.592	0.638	0.664	0.681
N cells GeoMx	-	-	-	-	-	-	269.83
ResNet 8µm	-	-	-	-	-	-	0.140 ± 0.006
ResNet 8µm merged	-	-	-	-	-	-	0.141
FFW I2C 8µm	-	-	-	-	-	-	0.226 ± 0.030
FFW I2C 8µm merged	-	-	-	-	-	-	0.243
GAT I2C 8µm	-	-	-	-	-	-	0.013 ± 0.016
GAT I2C 8µm merged	-	-	-	-	-	-	0.017
ResNet 12µm	-	-	-	-	-	-	0.233 ± 0.007
ResNet 12µm merged	-	-	-	-	-	-	0.236
FFW I2C 12µm	-	-	-	-	-	-	0.227 ± 0.032
FFW I2C 12µm merged	-	-	-	-	-	-	0.246
GAT I2C 12µm	-	-	-	-	-	-	0.196 ± 0.034
GAT I2C 12µm merged	-	-	-	-	-	-	0.217
ResNet 20µm	-	-	-	-	-	-	0.199 ± 0.006
ResNet 20µm merged	-	-	-	-	-	-	0.201
FFW I2C 20µm	-	-	-	-	-	-	0.221 ± 0.027
FFW I2C 20µm merged	-	-	-	-	-	-	0.241
GAT I2C 20µm	-	-	-	-	-	-	0.209 ± 0.028
GAT I2C 20µm merged	-	-	-	-	-	-	0.232

Table S1: Pearson correlation of predictions of linear evaluation head of the ResNet visual feature backbone, FFW Image2Count (I2C) and graph-based Image2Count models to counts of the test data from CosMx, t-CycIF and GeoMx datasets, averaged over all cross-validation runs from average per-gene correlation. Merged predictions were obtained by calculating the mean prediction over all cross-validation runs. DCA signifies correlation of previous predicted counts to imputed count data using DCA (deep-count autoencoder). K hop stands for a k-hop subgraph created from a central cell, expanding the graph to neighboring nodes k times. 36 (total 1800) subgraphs for the CosMx dataset and 900 (total 2700) subgraphs for the t-CycIF datset were created per image.

	Single Cell	1 hop	2 hop	3 hop	5 hop	8 hop	train size
N cells CosMx	1	7.68	21.28	42.95	109.69	266.01	483.71
ResNet	0.067 ± 0.034	0.125 ± 0.060	0.156 ± 0.072	0.157 ± 0.082	0.177 ± 0.089	0.238 ± 0.084	0.298 ± 0.079
ResNet merged	0.079	0.144	0.178	0.179	0.199	0.261	0.324
FFW I2C	0.084 ± 0.038	0.153 ± 0.061	0.202 ± 0.070	0.220 ± 0.083	0.274 ± 0.088	0.341 ± 0.085	0.394 ± 0.080
FFW I2C merged	0.120	0.213	0.272	0.299	0.358	0.423	0.475
GAT I2C	0.085 ± 0.038	0.155 ± 0.058	0.209 ± 0.061	0.232 ± 0.069	0.295 ± 0.061	0.369 ± 0.048	0.425 ± 0.040
GAT I2C merged	0.122	0.214	0.277	0.308	0.373	0.440	0.491
ResNet DCA	0.120 ± 0.091	0.193 ± 0.104	0.215 ± 0.102	0.200 ± 0.103	0.210 ± 0.099	0.275 ± 0.087	0.338 ± 0.078
ResNet DCA merged	0.141	0.222	0.244	0.225	0.234	0.301	0.366
FFW I2C DCA	0.201 ± 0.102	0.259 ± 0.104	0.289 ± 0.097	0.287 ± 0.103	0.325 ± 0.098	0.388 ± 0.088	0.437 ± 0.080
FFW I2C DCA merged	0.296	0.363	0.389	0.389	0.422	0.477	0.524
GAT I2C DCA	0.205 ± 0.099	0.269 ± 0.095	0.306 ± 0.081	0.309 ± 0.082	0.356 ± 0.065	0.424 ± 0.045	0.475 ± 0.035
GAT I2C DCA merged	0.303	0.371	0.404	0.408	0.447	0.500	0.544
N cells CycIF	1	7.78	19.85	38.25	95.88	234.84	438.54
ResNet	0.330 ± 0.013	0.470 ± 0.013	0.539 ± 0.012	0.590 ± 0.011	0.649 ± 0.010	0.688 ± 0.010	0.709 ± 0.009
ResNet merged	0.343	0.484	0.554	0.604	0.663	0.701	0.722
FFW I2C	0.471 ± 0.010	0.598 ± 0.008	0.656 ± 0.008	0.696 ± 0.008	0.739 ± 0.008	0.766 ± 0.008	0.779 ± 0.009
FFW I2C merged	0.528	0.641	0.690	0.725	0.763	0.787	0.799
GAT I2C	0.473 ± 0.017	0.597 ± 0.010	0.654 ± 0.009	0.694 ± 0.008	0.738 ± 0.007	0.765 ± 0.007	0.778 ± 0.007
GAT I2C merged	0.534	0.641	0.689	0.724	0.763	0.787	0.799
ResNet 2 Channels	0.258 ± 0.015	0.365 ± 0.010	0.444 ± 0.009	0.504 ± 0.009	0.568 ± 0.009	0.607 ± 0.009	0.627 ± 0.009
ResNet 2 Channels merged	0.269	0.378	0.458	0.518	0.581	0.619	0.638
FFW I2C 2 Channels	0.387 ± 0.016	0.479 ± 0.012	0.545 ± 0.011	0.595 ± 0.010	0.649 ± 0.009	0.683 ± 0.009	0.700 ± 0.009
FFW I2C 2 Channels merged	0.444	0.520	0.578	0.623	0.671	0.702	0.717
GAT I2C 2 Channels	0.392 ± 0.010	0.470 ± 0.008	0.534 ± 0.007	0.585 ± 0.006	0.640 ± 0.006	0.674 ± 0.007	0.692 ± 0.007
GAT I2C 2 Channels merged	0.446	0.516	0.573	0.619	0.669	0.700	0.717
ResNet no KM	0.314 ± 0.005	0.408 ± 0.006	0.474 ± 0.007	0.527 ± 0.007	0.590 ± 0.008	0.632 ± 0.008	0.655 ± 0.007
ResNet no KM merged	0.330	0.420	0.486	0.539	0.601	0.643	0.665
FFW I2C no KM	0.268 ± 0.023	0.458 ± 0.016	0.545 ± 0.016	0.604 ± 0.015	0.666 ± 0.014	0.704 ± 0.013	0.723 ± 0.012
FFW I2C no KM merged	0.456	0.573	0.632	0.674	0.720	0.748	0.763
GAT I2C no KM	0.231 ± 0.027	0.470 ± 0.015	0.558 ± 0.011	0.617 ± 0.009	0.679 ± 0.008	0.718 ± 0.008	0.736 ± 0.008
GAT I2C no KM merged	0.429	0.566	0.626	0.671	0.718	0.749	0.763
ResNet 8µm	0.336 ± 0.009	0.482 ± 0.009	0.554 ± 0.008	0.612 ± 0.007	0.673 ± 0.007	0.712 ± 0.006	0.730 ± 0.006
ResNet 8µm merged	0.358	0.498	0.568	0.625	0.684	0.721	0.738
FFW I2C 8µm	0.396 ± 0.026	0.537 ± 0.014	0.603 ± 0.013	0.649 ± 0.013	0.698 ± 0.013	0.728 ± 0.013	0.743 ± 0.013
FFW I2C 8µm merged	0.456	0.573	0.631	0.672	0.717	0.745	0.758
GAT I2C 8µm	0.382 ± 0.033	0.522 ± 0.023	0.590 ± 0.019	0.638 ± 0.018	0.690 ± 0.016	0.722 ± 0.016	0.738 ± 0.016
GAT I2C 8µm merged	0.454	0.564	0.624	0.668	0.715	0.744	0.758
ResNet 20µm	0.374 ± 0.029	0.487 ± 0.026	0.553 ± 0.026	0.603 ± 0.025	0.660 ± 0.023	0.696 ± 0.021	0.715 ± 0.020
ResNet 20µm merged	0.412	0.517	0.581	0.630	0.686	0.719	0.736
FFW I2C 20µm	0.443 ± 0.016	0.571 ± 0.008	0.631 ± 0.006	0.673 ± 0.006	0.721 ± 0.006	0.749 ± 0.006	0.762 ± 0.006
FFW I2C 20µm merged	0.494	0.603	0.657	0.695	0.738	0.764	0.776
GAT I2C 20µm	0.442 ± 0.034	0.561 ± 0.015	0.622 ± 0.012	0.666 ± 0.011	0.714 ± 0.010	0.743 ± 0.010	0.757 ± 0.010
GAT I2C 20µm merged	0.512	0.603	0.655	0.695	0.739	0.765	0.778
N cells GeoMx	-	-	-	-	-	-	269.83
ResNet 8µm	-	-	-	-	-	-	0.176 ± 0.008
ResNet 8µm merged	-	-	-	-	-	-	0.178
FFW I2C 8µm	-	-	-	-	-	-	0.263 ± 0.032
FFW I2C 8µm merged	-	-	-	-	-	-	0.277
GAT I2C 8µm	-	-	-	-	-	-	0.013 ± 0.016
GAT I2C 8µm merged	-	-	-	-	-	-	0.010
ResNet 12µm	-	-	-	-	-	-	0.257 ± 0.005
ResNet 12µm merged	-	-	-	-	-	-	0.261
FFW I2C 12µm	-	-	-	-	-	-	0.258 ± 0.038
FFW I2C 12µm merged	-	-	-	-	-	-	0.277
GAT I2C 12µm	-	-	-	-	-	-	0.230 ± 0.042
GAT I2C 12µm merged	-	-	-	-	-	-	0.245
ResNet 20µm	-	-	-	-	-	-	0.240 ± 0.005
ResNet 20µm merged	-	-	-	-	-	-	0.242
FFW I2C 20µm	-	-	-	-	-	-	0.255 ± 0.033
FFW I2C 20µm merged	-	-	-	-	-	-	0.273
GAT I2C 20µm	-	-	-	-	-	-	0.237 ± 0.036
GAT I2C 20µm merged	-	-	-	-	-	-	0.257

Table S2: Spearman correlation of predictions of linear evaluation head of the ResNet visual feature backbone, FFW Image2Count (I2C) and graph-based Image2Count prediction head models to counts of the test data from CosMx, t-CycIF and GeoMx datasets, averaged over all cross-validation runs from average per-gene spearman correlation. Merged predictions were obtained by calculating the mean prediction over all cross-validation runs. DCA signifies spearman correlation of previous predicted counts to imputed count data using DCA (deep-count autoencoder). K hop stands for a k-hop subgraph created from a central cell, expanding the graph to neighboring nodes k times. 36 (total 1800) subgraphs for the CosMx dataset and 900 (total 2700) subgraphs for the t-CycIF datset were created per image.

	Single Cell	1 hop	2 hop	3 hop	5 hop	8 hop	train size
N cells CosMx	1	7.68	21.28	42.95	109.69	266.01	483.71
ResNet	0.016 ± 0.002	0.059 ± 0.007	0.098 ± 0.012	0.131 ± 0.013	0.194 ± 0.014	0.265 ± 0.017	0.318 ± 0.020
ResNet merged	0.017	0.061	0.099	0.130	0.192	0.264	0.317
FFW I2C	0.019 ± 0.002	0.061 ± 0.011	0.098 ± 0.021	0.131 ± 0.025	0.198 ± 0.033	0.275 ± 0.040	0.333 ± 0.043
FFW I2C merged	0.021	0.067	0.112	0.147	0.220	0.295	0.359
GAT I2C	0.018 ± 0.002	0.059 ± 0.010	0.096 ± 0.021	0.129 ± 0.027	0.196 ± 0.034	0.275 ± 0.041	0.337 ± 0.043
GAT I2C merged	0.021	0.070	0.116	0.155	0.232	0.315	0.379
ResNet DCA	0.060 ± 0.009	0.115 ± 0.018	0.139 ± 0.022	0.155 ± 0.019	0.182 ± 0.016	0.229 ± 0.014	0.282 ± 0.015
ResNet DCA merged	0.058	0.113	0.138	0.149	0.177	0.229	0.283
FFW I2C DCA	0.092 ± 0.010	0.135 ± 0.030	0.151 ± 0.045	0.163 ± 0.048	0.200 ± 0.048	0.254 ± 0.051	0.312 ± 0.052
FFW I2C DCA merged	0.104	0.155	0.181	0.193	0.229	0.284	0.347
GAT I2C DCA	0.086 ± 0.010	0.128 ± 0.027	0.145 ± 0.044	0.157 ± 0.048	0.195 ± 0.049	0.256 ± 0.048	0.321 ± 0.045
GAT I2C DCA merged	0.103	0.157	0.192	0.207	0.254	0.314	0.379
N cells CycIF	1	7.78	19.85	38.25	95.88	234.84	438.54
ResNet	0.136 ± 0.007	0.238 ± 0.011	0.304 ± 0.014	0.365 ± 0.016	0.466 ± 0.017	0.573 ± 0.019	0.641 ± 0.021
ResNet merged	0.140	0.249	0.317	0.389	0.488	0.599	0.673
FFW I2C	0.246 ± 0.008	0.389 ± 0.009	0.475 ± 0.011	0.557 ± 0.013	0.685 ± 0.013	0.803 ± 0.014	0.874 ± 0.014
FFW I2C merged	0.302	0.459	0.550	0.641	0.761	0.881	0.943
GAT I2C	0.244 ± 0.017	0.389 ± 0.018	0.470 ± 0.019	0.553 ± 0.020	0.679 ± 0.022	0.798 ± 0.023	0.869 ± 0.023
GAT I2C merged	0.303	0.458	0.544	0.632	0.751	0.878	0.947
ResNet 2 Channels	0.102 ± 0.003	0.177 ± 0.006	0.230 ± 0.008	0.297 ± 0.006	0.399 ± 0.009	0.500 ± 0.010	0.563 ± 0.011
ResNet 2 Channels merged	0.105	0.188	0.241	0.303	0.410	0.516	0.578
FFW I2C 2 Channels	0.198 ± 0.012	0.295 ± 0.009	0.369 ± 0.011	0.445 ± 0.011	0.560 ± 0.012	0.673 ± 0.012	0.739 ± 0.012
FFW I2C 2 Channels merged	0.244	0.342	0.425	0.496	0.620	0.733	0.801
GAT I2C 2 Channels	0.202 ± 0.010	0.287 ± 0.010	0.356 ± 0.011	0.429 ± 0.012	0.544 ± 0.010	0.657 ± 0.010	0.724 ± 0.012
GAT I2C 2 Channels merged	0.250	0.343	0.411	0.496	0.612	0.727	0.794
ResNet no KM	0.109 ± 0.002	0.186 ± 0.004	0.240 ± 0.006	0.305 ± 0.008	0.407 ± 0.009	0.512 ± 0.011	0.577 ± 0.011
ResNet no KM merged	0.113	0.193	0.245	0.313	0.422	0.523	0.592
FFW I2C no KM	0.128 ± 0.016	0.242 ± 0.012	0.330 ± 0.015	0.418 ± 0.020	0.545 ± 0.023	0.669 ± 0.026	0.743 ± 0.028
FFW I2C no KM merged	0.221	0.371	0.467	0.550	0.679	0.801	0.877
GAT I2C no KM	0.096 ± 0.016	0.249 ± 0.014	0.348 ± 0.016	0.437 ± 0.016	0.570 ± 0.016	0.698 ± 0.019	0.775 ± 0.019
GAT I2C no KM merged	0.201	0.366	0.460	0.551	0.676	0.802	0.873
ResNet 8µm	0.135 ± 0.005	0.262 ± 0.011	0.346 ± 0.012	0.430 ± 0.012	0.557 ± 0.012	0.679 ± 0.013	0.756 ± 0.015
ResNet 8µm merged	0.146	0.282	0.366	0.447	0.576	0.706	0.772
FFW I2C 8µm	0.198 ± 0.021	0.338 ± 0.021	0.433 ± 0.022	0.518 ± 0.023	0.645 ± 0.029	0.766 ± 0.030	0.836 ± 0.031
FFW I2C 8µm merged	0.241	0.394	0.486	0.570	0.699	0.823	0.883
GAT I2C 8µm	0.187 ± 0.022	0.331 ± 0.024	0.422 ± 0.026	0.505 ± 0.030	0.632 ± 0.029	0.754 ± 0.033	0.823 ± 0.033
GAT I2C 8µm merged	0.239	0.386	0.482	0.566	0.684	0.810	0.881
ResNet 20µm	0.172 ± 0.018	0.283 ± 0.028	0.349 ± 0.034	0.413 ± 0.040	0.520 ± 0.045	0.632 ± 0.051	0.700 ± 0.053
ResNet 20µm merged	0.191	0.311	0.379	0.447	0.563	0.672	0.747
FFW I2C 20µm	0.248 ± 0.011	0.397 ± 0.008	0.486 ± 0.009	0.571 ± 0.011	0.696 ± 0.012	0.817 ± 0.015	0.885 ± 0.015
FFW I2C 20µm merged	0.298	0.455	0.546	0.624	0.757	0.873	0.945
GAT I2C 20µm	0.233 ± 0.028	0.376 ± 0.022	0.458 ± 0.020	0.544 ± 0.019	0.668 ± 0.020	0.784 ± 0.021	0.854 ± 0.020
GAT I2C 20µm merged	0.294	0.447	0.535	0.616	0.742	0.861	0.930
N cells GeoMx	-	-	-	-	-	-	269.83
ResNet 8µm	-	-	-	-	-	-	0.057 ± 0.003
ResNet 8µm merged	-	-	-	-	-	-	0.058
FFW I2C 8µm	-	-	-	-	-	-	0.062 ± 0.011
FFW I2C 8µm merged	-	-	-	-	-	-	0.067
GAT I2C 8µm	-	-	-	-	-	-	0.026 ± 0.005
GAT I2C 8µm merged	-	-	-	-	-	-	0.024
ResNet 12µm	-	-	-	-	-	-	0.056 ± 0.003
ResNet 12µm merged	-	-	-	-	-	-	0.055
FFW I2C 12µm	-	-	-	-	-	-	0.059 ± 0.013
FFW I2C 12µm merged	-	-	-	-	-	-	0.059
GAT I2C 12µm	-	-	-	-	-	-	0.052 ± 0.011
GAT I2C 12µm merged	-	-	-	-	-	-	0.055
ResNet 20µm	-	-	-	-	-	-	0.062 ± 0.005
ResNet 20µm merged	-	-	-	-	-	-	0.067
FFW I2C 20µm	-	-	-	-	-	-	0.059 ± 0.008
FFW I2C 20µm merged	-	-	-	-	-	-	0.061
GAT I2C 20µm	-	-	-	-	-	-	0.048 ± 0.011
GAT I2C 20µm merged	-	-	-	-	-	-	0.054

Table S3: Mutual information (MI) of predictions of linear evaluation head of the ResNet visual feature backbone, FFW Image2Count (I2C) and graph-based Image2Count models to counts of the test data from CosMx, t-CycIF and GeoMx datasets, averaged over all cross-validation runs from average per-gene MI. Merged predictions were obtained by calculating the mean prediction over all cross-validation runs. DCA signifies MI of previous predicted counts to imputed count data using DCA (deep-count autoencoder). K hop stands for a k-hop subgraph created from a central cell, expanding the graph to neighboring nodes k times. 36 (total 1800) subgraphs for the CosMx dataset and 900 (total 2700) subgraphs for the t-CycIF datset were created per image.

	Single Cell	1 hop	2 hop	3 hop	5 hop	8 hop	train size
N cells CosMx	1	7.68	21.28	42.95	109.69	266.01	483.71
ResNet	0.131 ± 0.002	0.541 ± 0.024	0.806 ± 0.047	0.929 ± 0.061	0.958 ± 0.066	0.897 ± 0.061	0.856 ± 0.058
ResNet merged	0.128	0.526	0.783	0.902	0.927	0.866	0.824
FFW I2C	0.133 ± 0.007	0.546 ± 0.030	0.803 ± 0.048	0.913 ± 0.061	0.927 ± 0.069	0.865 ± 0.067	0.824 ± 0.064
FFW I2C merged	0.124	0.509	0.747	0.849	0.860	0.798	0.758
GAT I2C	0.133 ± 0.006	0.555 ± 0.031	0.812 ± 0.041	0.920 ± 0.048	0.929 ± 0.053	0.863 ± 0.052	0.820 ± 0.050
GAT I2C merged	0.127	0.525	0.768	0.871	0.879	0.814	0.773
ResNet DCA	0.095 ± 0.001	0.366 ± 0.002	0.530 ± 0.002	0.620 ± 0.002	0.683 ± 0.004	0.696 ± 0.005	0.691 ± 0.005
ResNet DCA merged	0.091	0.347	0.498	0.581	0.640	0.653	0.649
FFW I2C DCA	0.099 ± 0.009	0.385 ± 0.038	0.553 ± 0.055	0.637 ± 0.060	0.691 ± 0.058	0.699 ± 0.053	0.693 ± 0.048
FFW I2C DCA merged	0.089	0.338	0.477	0.548	0.597	0.608	0.605
GAT I2C DCA	0.097 ± 0.006	0.378 ± 0.027	0.537 ± 0.036	0.615 ± 0.038	0.663 ± 0.036	0.667 ± 0.033	0.660 ± 0.032
GAT I2C DCA merged	0.090	0.338	0.475	0.544	0.591	0.599	0.596
N cells CycIF	1	7.78	19.85	38.25	95.88	234.84	438.54
ResNet	1.834 ± 0.124	1.108 ± 0.060	0.913 ± 0.048	0.813 ± 0.042	0.712 ± 0.035	0.643 ± 0.033	0.603 ± 0.032
ResNet merged	1.649	1.019	0.845	0.756	0.665	0.604	0.567
FFW I2C	1.141 ± 0.051	0.762 ± 0.021	0.641 ± 0.019	0.579 ± 0.018	0.515 ± 0.019	0.474 ± 0.020	0.452 ± 0.021
FFW I2C merged	0.959	0.668	0.569	0.517	0.463	0.428	0.408
GAT I2C	1.106 ± 0.094	0.760 ± 0.024	0.641 ± 0.016	0.578 ± 0.014	0.516 ± 0.013	0.474 ± 0.013	0.452 ± 0.014
GAT I2C merged	0.927	0.669	0.568	0.515	0.461	0.425	0.406
ResNet 2 Channels	2.285 ± 0.197	1.342 ± 0.066	1.090 ± 0.043	0.956 ± 0.031	0.835 ± 0.023	0.762 ± 0.020	0.727 ± 0.019
ResNet 2 Channels merged	1.955	1.217	1.003	0.889	0.784	0.721	0.689
FFW I2C 2 Channels	1.331 ± 0.112	0.935 ± 0.038	0.793 ± 0.024	0.720 ± 0.019	0.650 ± 0.017	0.604 ± 0.017	0.579 ± 0.017
FFW I2C 2 Channels merged	1.102	0.830	0.716	0.657	0.598	0.559	0.538
GAT I2C 2 Channels	1.256 ± 0.053	0.959 ± 0.040	0.821 ± 0.027	0.748 ± 0.019	0.676 ± 0.016	0.628 ± 0.017	0.601 ± 0.019
GAT I2C 2 Channels merged	1.073	0.841	0.728	0.667	0.606	0.564	0.541
ResNet no KM	2.169 ± 0.047	1.266 ± 0.026	1.026 ± 0.023	0.914 ± 0.023	0.804 ± 0.023	0.730 ± 0.023	0.692 ± 0.023
ResNet no KM merged	1.918	1.172	0.960	0.860	0.760	0.692	0.657
FFW I2C no KM	2.503 ± 0.369	1.236 ± 0.105	0.957 ± 0.069	0.823 ± 0.052	0.698 ± 0.036	0.622 ± 0.029	0.586 ± 0.027
FFW I2C no KM merged	1.194	0.814	0.688	0.623	0.558	0.517	0.495
GAT I2C no KM	2.766 ± 0.344	1.093 ± 0.079	0.851 ± 0.055	0.744 ± 0.047	0.647 ± 0.042	0.586 ± 0.040	0.557 ± 0.040
GAT I2C no KM merged	1.163	0.808	0.687	0.626	0.563	0.520	0.498
ResNet 8µm	2.285 ± 0.080	0.950 ± 0.020	0.766 ± 0.018	0.683 ± 0.017	0.605 ± 0.017	0.554 ± 0.017	0.529 ± 0.018
ResNet 8µm merged	1.901	0.890	0.728	0.653	0.581	0.534	0.511
FFW I2C 8µm	1.431 ± 0.283	0.852 ± 0.056	0.708 ± 0.034	0.643 ± 0.031	0.581 ± 0.033	0.541 ± 0.037	0.522 ± 0.038
FFW I2C 8µm merged	1.096	0.754	0.640	0.587	0.533	0.497	0.479
GAT I2C 8µm	1.384 ± 0.180	0.891 ± 0.040	0.750 ± 0.034	0.683 ± 0.032	0.615 ± 0.030	0.570 ± 0.030	0.548 ± 0.029
GAT I2C 8µm merged	1.059	0.795	0.677	0.620	0.561	0.521	0.501
ResNet 20µm	2.371 ± 0.629	1.163 ± 0.181	0.919 ± 0.125	0.807 ± 0.100	0.696 ± 0.076	0.629 ± 0.061	0.595 ± 0.054
ResNet 20µm merged	1.680	0.966	0.790	0.707	0.623	0.573	0.546
FFW I2C 20µm	1.261 ± 0.116	0.805 ± 0.030	0.677 ± 0.019	0.615 ± 0.017	0.552 ± 0.018	0.513 ± 0.020	0.492 ± 0.021
FFW I2C 20µm merged	1.046	0.727	0.620	0.567	0.512	0.477	0.458
GAT I2C 20µm	1.223 ± 0.213	0.827 ± 0.037	0.699 ± 0.027	0.635 ± 0.024	0.570 ± 0.024	0.527 ± 0.026	0.505 ± 0.027
GAT I2C 20µm merged	0.972	0.738	0.628	0.572	0.514	0.476	0.456
N cells GeoMx	-	-	-	-	-	-	269.83
ResNet 8µm	-	-	-	-	-	-	2.555 ± 0.044
ResNet 8µm merged	-	-	-	-	-	-	2.553
FFW I2C 8µm	-	-	-	-	-	-	0.795 ± 0.026
FFW I2C 8µm merged	-	-	-	-	-	-	0.759
GAT I2C 8µm	-	-	-	-	-	-	1.223 ± 0.219
GAT I2C 8µm merged	-	-	-	-	-	-	1.039
ResNet 12µm	-	-	-	-	-	-	0.763 ± 0.009
ResNet 12µm merged	-	-	-	-	-	-	0.750
FFW I2C 12µm	-	-	-	-	-	-	0.815 ± 0.031
FFW I2C 12µm merged	-	-	-	-	-	-	0.765
GAT I2C 12µm	-	-	-	-	-	-	0.845 ± 0.060
GAT I2C 12µm merged	-	-	-	-	-	-	0.758
ResNet 20µm	-	-	-	-	-	-	0.955 ± 0.037
ResNet 20µm merged	-	-	-	-	-	-	0.947
FFW I2C 20µm	-	-	-	-	-	-	0.837 ± 0.031
FFW I2C 20µm merged	-	-	-	-	-	-	0.789
GAT I2C 20µm	-	-	-	-	-	-	0.838 ± 0.031
GAT I2C 20µm merged	-	-	-	-	-	-	0.790

Table S4: Mean squared error (MSE) of log1p predictions of linear evaluation head of the ResNet visual feature backbone, FFW Image2Count (I2C) and graph-based Image2Count models to counts of the test data from CosMx, t-CycIF and GeoMx datasets, averaged over all cross-validation runs. Merged predictions were obtained by calculating the mean prediction over all cross-validation runs. DCA signifies MSE of previous predicted counts to imputed count data using DCA (deep-count autoencoder). K hop stands for a k-hop subgraph created from a central cell, expanding the graph to neighboring nodes k times. 36 (total 1800) subgraphs for the CosMx dataset and 900 (total 2700) subgraphs for the t-CycIF datset were created per image.

	Single Cell	1 hop	2 hop	3 hop	5 hop	8 hop	train size
N cells CosMx	1	7.68	21.28	42.95	109.69	266.01	483.71
ResNet	0.491 ± 0.016	0.647 ± 0.020	0.680 ± 0.021	0.694 ± 0.021	0.706 ± 0.021	0.715 ± 0.021	0.719 ± 0.022
ResNet merged	0.503	0.660	0.693	0.707	0.719	0.727	0.731
FFW I2C	0.474 ± 0.016	0.627 ± 0.018	0.660 ± 0.017	0.673 ± 0.017	0.685 ± 0.017	0.693 ± 0.018	0.697 ± 0.019
FFW I2C merged	0.487	0.642	0.675	0.688	0.700	0.708	0.712
GAT I2C	0.479 ± 0.015	0.630 ± 0.019	0.662 ± 0.020	0.677 ± 0.020	0.689 ± 0.021	0.698 ± 0.022	0.702 ± 0.022
GAT I2C merged	0.490	0.643	0.676	0.690	0.702	0.710	0.714
ResNet DCA	0.444 ± 0.011	0.528 ± 0.014	0.547 ± 0.015	0.558 ± 0.015	0.572 ± 0.015	0.584 ± 0.016	0.589 ± 0.016
ResNet DCA merged	0.454	0.539	0.557	0.569	0.582	0.594	0.600
FFW I2C DCA	0.429 ± 0.015	0.510 ± 0.016	0.530 ± 0.015	0.542 ± 0.015	0.555 ± 0.015	0.566 ± 0.016	0.572 ± 0.016
FFW I2C DCA merged	0.440	0.522	0.542	0.553	0.567	0.578	0.584
GAT I2C DCA	0.432 ± 0.013	0.512 ± 0.017	0.531 ± 0.017	0.543 ± 0.017	0.557 ± 0.018	0.569 ± 0.018	0.575 ± 0.018
GAT I2C DCA merged	0.442	0.523	0.542	0.553	0.567	0.579	0.585
N cells CycIF	1	7.78	19.85	38.25	95.88	234.84	438.54
ResNet	0.809 ± 0.016	0.879 ± 0.006	0.896 ± 0.005	0.904 ± 0.004	0.911 ± 0.003	0.917 ± 0.004	0.920 ± 0.004
ResNet merged	0.829	0.888	0.903	0.910	0.916	0.921	0.924
FFW I2C	0.826 ± 0.006	0.891 ± 0.004	0.904 ± 0.004	0.910 ± 0.004	0.916 ± 0.004	0.920 ± 0.004	0.922 ± 0.004
FFW I2C merged	0.852	0.903	0.914	0.919	0.923	0.927	0.929
GAT I2C	0.816 ± 0.023	0.889 ± 0.007	0.903 ± 0.006	0.909 ± 0.006	0.915 ± 0.006	0.920 ± 0.006	0.922 ± 0.006
GAT I2C merged	0.851	0.903	0.914	0.919	0.923	0.927	0.929
ResNet 2 Channels	0.742 ± 0.036	0.830 ± 0.017	0.852 ± 0.012	0.864 ± 0.009	0.874 ± 0.006	0.883 ± 0.005	0.886 ± 0.004
ResNet 2 Channels merged	0.772	0.843	0.863	0.872	0.880	0.888	0.891
FFW I2C 2 Channels	0.807 ± 0.009	0.870 ± 0.004	0.885 ± 0.004	0.892 ± 0.004	0.898 ± 0.004	0.903 ± 0.004	0.906 ± 0.004
FFW I2C 2 Channels merged	0.833	0.880	0.892	0.898	0.903	0.907	0.909
GAT I2C 2 Channels	0.811 ± 0.007	0.863 ± 0.006	0.878 ± 0.004	0.885 ± 0.003	0.891 ± 0.003	0.896 ± 0.003	0.898 ± 0.003
GAT I2C 2 Channels merged	0.835	0.874	0.886	0.892	0.897	0.901	0.903
ResNet no KM	0.767 ± 0.006	0.844 ± 0.005	0.866 ± 0.004	0.876 ± 0.003	0.885 ± 0.003	0.892 ± 0.003	0.895 ± 0.003
ResNet no KM merged	0.787	0.856	0.875	0.883	0.891	0.897	0.900
FFW I2C no KM	0.621 ± 0.038	0.806 ± 0.022	0.849 ± 0.018	0.867 ± 0.016	0.881 ± 0.015	0.890 ± 0.014	0.895 ± 0.014
FFW I2C no KM merged	0.781	0.873	0.892	0.900	0.907	0.912	0.915
GAT I2C no KM	0.586 ± 0.034	0.811 ± 0.020	0.854 ± 0.016	0.870 ± 0.015	0.882 ± 0.014	0.891 ± 0.013	0.895 ± 0.013
GAT I2C no KM merged	0.768	0.867	0.885	0.892	0.898	0.904	0.906
ResNet 8µm	0.729 ± 0.008	0.864 ± 0.003	0.888 ± 0.003	0.897 ± 0.003	0.904 ± 0.003	0.911 ± 0.003	0.914 ± 0.003
ResNet 8µm merged	0.752	0.871	0.892	0.900	0.907	0.913	0.916
FFW I2C 8µm	0.777 ± 0.033	0.876 ± 0.007	0.893 ± 0.006	0.899 ± 0.006	0.905 ± 0.006	0.910 ± 0.006	0.913 ± 0.006
FFW I2C 8µm merged	0.821	0.890	0.902	0.907	0.912	0.916	0.918
GAT I2C 8µm	0.741 ± 0.046	0.860 ± 0.013	0.880 ± 0.011	0.888 ± 0.010	0.894 ± 0.010	0.899 ± 0.010	0.902 ± 0.009
GAT I2C 8µm merged	0.803	0.880	0.894	0.899	0.904	0.909	0.911
ResNet 20µm	0.764 ± 0.032	0.865 ± 0.011	0.884 ± 0.009	0.892 ± 0.008	0.900 ± 0.008	0.906 ± 0.007	0.909 ± 0.007
ResNet 20µm merged	0.796	0.877	0.892	0.900	0.906	0.911	0.914
FFW I2C 20µm	0.812 ± 0.009	0.885 ± 0.004	0.898 ± 0.003	0.903 ± 0.003	0.908 ± 0.003	0.913 ± 0.004	0.915 ± 0.004
FFW I2C 20µm merged	0.838	0.894	0.905	0.909	0.914	0.918	0.920
GAT I2C 20µm	0.784 ± 0.043	0.873 ± 0.011	0.889 ± 0.009	0.895 ± 0.009	0.901 ± 0.008	0.906 ± 0.008	0.908 ± 0.008
GAT I2C 20µm merged	0.833	0.888	0.900	0.904	0.909	0.913	0.915
N cells GeoMx	-	-	-	-	-	-	269.83
ResNet 8µm	-	-	-	-	-	-	0.784 ± 0.003
ResNet 8µm merged	-	-	-	-	-	-	0.789
FFW I2C 8µm	-	-	-	-	-	-	0.794 ± 0.004
FFW I2C 8µm merged	-	-	-	-	-	-	0.804
GAT I2C 8µm	-	-	-	-	-	-	0.732 ± 0.018
GAT I2C 8µm merged	-	-	-	-	-	-	0.754
ResNet 12µm	-	-	-	-	-	-	0.803 ± 0.005
ResNet 12µm merged	-	-	-	-	-	-	0.808
FFW I2C 12µm	-	-	-	-	-	-	0.793 ± 0.003
FFW I2C 12µm merged	-	-	-	-	-	-	0.806
GAT I2C 12µm	-	-	-	-	-	-	0.789 ± 0.007
GAT I2C 12µm merged	-	-	-	-	-	-	0.805
ResNet 20µm	-	-	-	-	-	-	0.783 ± 0.002
ResNet 20µm merged	-	-	-	-	-	-	0.786
FFW I2C 20µm	-	-	-	-	-	-	0.787 ± 0.004
FFW I2C 20µm merged	-	-	-	-	-	-	0.799
GAT I2C 20µm	-	-	-	-	-	-	0.792 ± 0.005
GAT I2C 20µm merged	-	-	-	-	-	-	0.804

Table S5: Cosine similarity of predictions of linear evaluation head of the ResNet visual feature backbone, FFW Image2Count (I2C) and graph-based Image2Count models to counts of the test data from CosMx, t-CycIF and GeoMx datasets, averaged over all cross-validation runs. Merged predictions were obtained by calculating the mean prediction over all cross-validation runs. DCA signifies MSE of previous predicted counts to imputed count data using DCA (deep-count autoencoder). K hop stands for a k-hop subgraph created from a central cell, expanding the graph to neighboring nodes k times. 36 (total 1800) subgraphs for the CosMx dataset and 900 (total 2700) subgraphs for the t-CycIF datset were created per image.

	Single Cell	1 hop	2 hop	3 hop	5 hop	8 hop	train size
N cells CosMx	1	7.68	21.28	42.95	109.69	266.01	483.71
ResNet	0.391 ± 0.007	0.206 ± 0.005	0.150 ± 0.005	0.127 ± 0.004	0.111 ± 0.004	0.103 ± 0.004	0.101 ± 0.004
ResNet merged	0.389	0.203	0.147	0.124	0.107	0.100	0.097
FFW I2C	0.397 ± 0.005	0.210 ± 0.004	0.154 ± 0.003	0.130 ± 0.003	0.113 ± 0.003	0.106 ± 0.003	0.103 ± 0.003
FFW I2C merged	0.392	0.203	0.147	0.124	0.107	0.099	0.097
GAT I2C	0.394 ± 0.005	0.208 ± 0.005	0.152 ± 0.004	0.129 ± 0.004	0.112 ± 0.004	0.105 ± 0.004	0.102 ± 0.004
GAT I2C merged	0.389	0.202	0.146	0.123	0.106	0.099	0.096
ResNet DCA	0.198 ± 0.002	0.145 ± 0.002	0.134 ± 0.002	0.129 ± 0.002	0.124 ± 0.002	0.120 ± 0.002	0.118 ± 0.002
ResNet DCA merged	0.194	0.141	0.131	0.126	0.120	0.117	0.115
FFW I2C DCA	0.201 ± 0.004	0.148 ± 0.003	0.137 ± 0.003	0.131 ± 0.003	0.126 ± 0.003	0.122 ± 0.003	0.120 ± 0.003
FFW I2C DCA merged	0.194	0.140	0.130	0.125	0.119	0.115	0.113
GAT I2C DCA	0.199 ± 0.003	0.147 ± 0.004	0.136 ± 0.004	0.131 ± 0.004	0.125 ± 0.004	0.121 ± 0.004	0.119 ± 0.004
GAT I2C DCA merged	0.193	0.140	0.130	0.124	0.119	0.115	0.113
N cells CycIF	1	7.78	19.85	38.25	95.88	234.84	438.54
ResNet	0.095 ± 0.007	0.063 ± 0.003	0.053 ± 0.002	0.049 ± 0.002	0.045 ± 0.002	0.041 ± 0.002	0.040 ± 0.002
ResNet merged	0.086	0.058	0.050	0.046	0.042	0.039	0.038
FFW I2C	0.085 ± 0.003	0.055 ± 0.002	0.047 ± 0.001	0.044 ± 0.001	0.040 ± 0.002	0.038 ± 0.002	0.036 ± 0.002
FFW I2C merged	0.072	0.048	0.042	0.039	0.036	0.034	0.033
GAT I2C	0.087 ± 0.010	0.055 ± 0.002	0.047 ± 0.002	0.044 ± 0.002	0.040 ± 0.002	0.037 ± 0.002	0.036 ± 0.002
GAT I2C merged	0.071	0.048	0.042	0.039	0.036	0.033	0.032
ResNet 2 Channels	0.127 ± 0.015	0.085 ± 0.006	0.073 ± 0.005	0.067 ± 0.004	0.061 ± 0.003	0.057 ± 0.002	0.056 ± 0.002
ResNet 2 Channels merged	0.112	0.078	0.068	0.063	0.058	0.055	0.053
FFW I2C 2 Channels	0.097 ± 0.006	0.068 ± 0.002	0.059 ± 0.002	0.055 ± 0.002	0.051 ± 0.002	0.048 ± 0.002	0.046 ± 0.002
FFW I2C 2 Channels merged	0.083	0.062	0.054	0.051	0.047	0.045	0.044
GAT I2C 2 Channels	0.094 ± 0.004	0.070 ± 0.003	0.062 ± 0.002	0.057 ± 0.001	0.053 ± 0.001	0.050 ± 0.001	0.049 ± 0.001
GAT I2C 2 Channels merged	0.081	0.063	0.056	0.053	0.049	0.047	0.045
ResNet no KM	0.114 ± 0.002	0.078 ± 0.002	0.066 ± 0.002	0.061 ± 0.002	0.055 ± 0.002	0.051 ± 0.002	0.049 ± 0.002
ResNet no KM merged	0.104	0.072	0.062	0.057	0.052	0.048	0.047
FFW I2C no KM	0.193 ± 0.020	0.096 ± 0.009	0.074 ± 0.006	0.064 ± 0.005	0.056 ± 0.005	0.050 ± 0.004	0.048 ± 0.004
FFW I2C no KM merged	0.102	0.061	0.052	0.047	0.043	0.040	0.039
GAT I2C no KM	0.208 ± 0.017	0.089 ± 0.008	0.068 ± 0.006	0.060 ± 0.005	0.053 ± 0.005	0.049 ± 0.005	0.046 ± 0.004
GAT I2C no KM merged	0.104	0.063	0.054	0.049	0.045	0.042	0.041
ResNet 8µm	0.131 ± 0.003	0.068 ± 0.002	0.056 ± 0.001	0.051 ± 0.001	0.046 ± 0.001	0.043 ± 0.002	0.041 ± 0.002
ResNet 8µm merged	0.118	0.064	0.053	0.049	0.045	0.042	0.040
FFW I2C 8µm	0.109 ± 0.017	0.063 ± 0.004	0.054 ± 0.003	0.050 ± 0.003	0.046 ± 0.004	0.044 ± 0.004	0.042 ± 0.004
FFW I2C 8µm merged	0.087	0.056	0.048	0.045	0.042	0.040	0.039
GAT I2C 8µm	0.120 ± 0.021	0.069 ± 0.005	0.059 ± 0.004	0.054 ± 0.004	0.050 ± 0.004	0.047 ± 0.004	0.046 ± 0.004
GAT I2C 8µm merged	0.088	0.060	0.052	0.049	0.045	0.043	0.041
ResNet 20µm	0.114 ± 0.016	0.068 ± 0.005	0.058 ± 0.004	0.053 ± 0.003	0.048 ± 0.003	0.045 ± 0.003	0.043 ± 0.003
ResNet 20µm merged	0.095	0.061	0.053	0.049	0.045	0.042	0.041
FFW I2C 20µm	0.093 ± 0.006	0.059 ± 0.002	0.051 ± 0.002	0.047 ± 0.002	0.044 ± 0.002	0.041 ± 0.002	0.040 ± 0.002
FFW I2C 20µm merged	0.079	0.053	0.047	0.044	0.040	0.038	0.037
GAT I2C 20µm	0.100 ± 0.020	0.063 ± 0.004	0.054 ± 0.003	0.050 ± 0.003	0.046 ± 0.003	0.044 ± 0.003	0.042 ± 0.003
GAT I2C 20µm merged	0.077	0.055	0.048	0.045	0.042	0.039	0.038
N cells GeoMx	-	-	-	-	-	-	269.83
ResNet 8µm	-	-	-	-	-	-	0.095 ± 0.001
ResNet 8µm merged	-	-	-	-	-	-	0.093
FFW I2C 8µm	-	-	-	-	-	-	0.068 ± 0.002
FFW I2C 8µm merged	-	-	-	-	-	-	0.065
GAT I2C 8µm	-	-	-	-	-	-	0.101 ± 0.009
GAT I2C 8µm merged	-	-	-	-	-	-	0.090
ResNet 12µm	-	-	-	-	-	-	0.065 ± 0.001
ResNet 12µm merged	-	-	-	-	-	-	0.064
FFW I2C 12µm	-	-	-	-	-	-	0.071 ± 0.003
FFW I2C 12µm merged	-	-	-	-	-	-	0.066
GAT I2C 12µm	-	-	-	-	-	-	0.073 ± 0.002
GAT I2C 12µm merged	-	-	-	-	-	-	0.067
ResNet 20µm	-	-	-	-	-	-	0.074 ± 0.001
ResNet 20µm merged	-	-	-	-	-	-	0.073
FFW I2C 20µm	-	-	-	-	-	-	0.073 ± 0.003
FFW I2C 20µm merged	-	-	-	-	-	-	0.068
GAT I2C 20µm	-	-	-	-	-	-	0.072 ± 0.003
GAT I2C 20µm merged	-	-	-	-	-	-	0.067

Table S6: Jenson Shannon divergence of predictions of linear evaluation head of the ResNet visual feature backbone, FFW Image2Count (I2C) and graph-based Image2Count models to counts of the test data from CosMx, t-CycIF and GeoMx datasets, averaged over all cross-validation runs (6 for CosMx, 10 for CycIF and GeoMx). Merged predictions were obtained by calculating the mean prediction over all cross-validation runs. DCA signifies models trained on raw data with correlation calculated from predicted counts and imputed count data using DCA (deep-count autoencoder). K hop stands for a k-hop subgraph created from a central cell, expanding the graph to neighboring nodes k times. For each image of the CosMx dataset, $6 \times 6 = 36$ subgraphs for a total of 1800 were created. For the t-CycIF dataset, $30 \times 30 = 900$ subgraphs were created for a total of 2700 subgraphs. Edges were determined via k-NN of spatial cell positions with number of neighbors set to 6. N Cells shows the (average) number of cells whose total expression was used to calculate correlation.

	Single Cell	1 hop	2 hop	3 hop	5 hop	8 hop	train size
N cells CosMx	1	7.68	21.28	42.95	109.69	266.01	483.71
ResNet	0.322 ± 0.018	0.376 ± 0.020	0.450 ± 0.023	0.499 ± 0.024	0.572 ± 0.021	0.639 ± 0.017	0.673 ± 0.015
ResNet merged	0.326	0.383	0.457	0.506	0.579	0.647	0.681
FFW I2C	0.320 ± 0.016	0.358 ± 0.024	0.432 ± 0.029	0.481 ± 0.031	0.556 ± 0.029	0.623 ± 0.026	0.657 ± 0.024
FFW I2C merged	0.332	0.379	0.455	0.504	0.578	0.645	0.679
GAT I2C	0.321 ± 0.012	0.366 ± 0.025	0.440 ± 0.031	0.490 ± 0.033	0.565 ± 0.031	0.631 ± 0.027	0.665 ± 0.025
GAT I2C merged	0.330	0.386	0.463	0.514	0.589	0.655	0.688
ResNet DCA	0.581 ± 0.008	0.570 ± 0.013	0.618 ± 0.015	0.644 ± 0.015	0.677 ± 0.014	0.707 ± 0.012	0.724 ± 0.011
ResNet DCA merged	0.596	0.589	0.635	0.660	0.690	0.719	0.735
FFW I2C DCA	0.565 ± 0.031	0.540 ± 0.026	0.589 ± 0.026	0.619 ± 0.026	0.656 ± 0.025	0.689 ± 0.024	0.706 ± 0.023
FFW I2C DCA merged	0.604	0.589	0.636	0.661	0.692	0.719	0.734
GAT I2C DCA	0.580 ± 0.012	0.554 ± 0.022	0.602 ± 0.025	0.630 ± 0.026	0.667 ± 0.026	0.699 ± 0.025	0.716 ± 0.024
GAT I2C DCA merged	0.608	0.598	0.646	0.672	0.703	0.730	0.744
N cells CycIF	1	7.78	19.85	38.25	95.88	234.84	438.54
ResNet	0.663 ± 0.018	0.768 ± 0.045	0.799 ± 0.033	0.815 ± 0.021	0.836 ± 0.013	0.851 ± 0.006	0.860 ± 0.007
ResNet merged	0.683	0.766	0.798	0.817	0.839	0.857	0.866
FFW I2C	0.751 ± 0.008	0.807 ± 0.006	0.830 ± 0.007	0.846 ± 0.006	0.862 ± 0.007	0.875 ± 0.007	0.883 ± 0.006
FFW I2C merged	0.787	0.829	0.848	0.860	0.875	0.887	0.893
GAT I2C	0.760 ± 0.016	0.811 ± 0.005	0.834 ± 0.004	0.849 ± 0.004	0.866 ± 0.004	0.880 ± 0.004	0.887 ± 0.004
GAT I2C merged	0.797	0.832	0.851	0.864	0.878	0.890	0.896
ResNet 2 Channels	0.585 ± 0.019	0.713 ± 0.061	0.753 ± 0.044	0.776 ± 0.030	0.802 ± 0.021	0.820 ± 0.013	0.830 ± 0.009
ResNet 2 Channels merged	0.623	0.711	0.752	0.777	0.804	0.823	0.833
FFW I2C 2 Channels	0.705 ± 0.019	0.757 ± 0.009	0.784 ± 0.005	0.804 ± 0.006	0.824 ± 0.005	0.840 ± 0.005	0.850 ± 0.005
FFW I2C 2 Channels merged	0.747	0.777	0.801	0.817	0.835	0.850	0.858
GAT I2C 2 Channels	0.719 ± 0.008	0.752 ± 0.007	0.780 ± 0.006	0.799 ± 0.006	0.821 ± 0.005	0.837 ± 0.006	0.847 ± 0.006
GAT I2C 2 Channels merged	0.756	0.777	0.801	0.817	0.836	0.851	0.859
ResNet no KM	0.620 ± 0.009	0.740 ± 0.070	0.775 ± 0.053	0.796 ± 0.042	0.819 ± 0.030	0.839 ± 0.025	0.849 ± 0.022
ResNet no KM merged	0.648	0.733	0.771	0.792	0.818	0.838	0.849
FFW I2C no KM	0.544 ± 0.051	0.716 ± 0.031	0.766 ± 0.020	0.794 ± 0.015	0.823 ± 0.010	0.845 ± 0.009	0.856 ± 0.009
FFW I2C no KM merged	0.730	0.791	0.817	0.832	0.850	0.865	0.873
GAT I2C no KM	0.518 ± 0.050	0.736 ± 0.016	0.784 ± 0.011	0.808 ± 0.010	0.834 ± 0.009	0.852 ± 0.009	0.862 ± 0.008
GAT I2C no KM merged	0.742	0.795	0.818	0.833	0.851	0.865	0.873
ResNet 8µm	0.607 ± 0.009	0.774 ± 0.021	0.803 ± 0.008	0.822 ± 0.007	0.842 ± 0.004	0.858 ± 0.004	0.866 ± 0.004
ResNet 8µm merged	0.646	0.778	0.808	0.826	0.846	0.862	0.869
FFW I2C 8µm	0.701 ± 0.041	0.783 ± 0.011	0.809 ± 0.010	0.826 ± 0.010	0.843 ± 0.011	0.857 ± 0.011	0.865 ± 0.011
FFW I2C 8µm merged	0.755	0.804	0.826	0.840	0.855	0.868	0.875
GAT I2C 8µm	0.709 ± 0.041	0.777 ± 0.014	0.805 ± 0.013	0.821 ± 0.012	0.840 ± 0.011	0.854 ± 0.010	0.862 ± 0.010
GAT I2C 8µm merged	0.771	0.801	0.823	0.836	0.852	0.866	0.873
ResNet 20µm	0.641 ± 0.045	0.765 ± 0.038	0.796 ± 0.024	0.813 ± 0.016	0.835 ± 0.012	0.853 ± 0.010	0.861 ± 0.008
ResNet 20µm merged	0.703	0.779	0.808	0.825	0.846	0.861	0.869
FFW I2C 20µm	0.730 ± 0.019	0.795 ± 0.007	0.819 ± 0.007	0.834 ± 0.006	0.851 ± 0.007	0.865 ± 0.007	0.873 ± 0.006
FFW I2C 20µm merged	0.769	0.813	0.833	0.846	0.862	0.875	0.881
GAT I2C 20µm	0.741 ± 0.034	0.795 ± 0.008	0.819 ± 0.009	0.834 ± 0.009	0.852 ± 0.010	0.866 ± 0.010	0.873 ± 0.010
GAT I2C 20µm merged	0.791	0.817	0.837	0.850	0.866	0.878	0.885
N cells GeoMx	-	-	-	-	-	-	269.83
ResNet 8µm	-	-	-	-	-	-	0.476 ± 0.007
ResNet 8µm merged	-	-	-	-	-	-	0.478
FFW I2C 8µm	-	-	-	-	-	-	0.795 ± 0.007
FFW I2C 8µm merged	-	-	-	-	-	-	0.804
GAT I2C 8µm	-	-	-	-	-	-	0.710 ± 0.021
GAT I2C 8µm merged	-	-	-	-	-	-	0.729
ResNet 12µm	-	-	-	-	-	-	0.793 ± 0.003
ResNet 12µm merged	-	-	-	-	-	-	0.796
FFW I2C 12µm	-	-	-	-	-	-	0.790 ± 0.008
FFW I2C 12µm merged	-	-	-	-	-	-	0.802
GAT I2C 12µm	-	-	-	-	-	-	0.784 ± 0.009
GAT I2C 12µm merged	-	-	-	-	-	-	0.797
ResNet 20µm	-	-	-	-	-	-	0.758 ± 0.009
ResNet 20µm merged	-	-	-	-	-	-	0.760
FFW I2C 20µm	-	-	-	-	-	-	0.784 ± 0.005
FFW I2C 20µm merged	-	-	-	-	-	-	0.797
GAT I2C 20µm	-	-	-	-	-	-	0.783 ± 0.009
GAT I2C 20µm merged	-	-	-	-	-	-	0.795

Table S7: Structural similarity index of predictions of linear evaluation head of the ResNet visual feature backbone, FFW Image2Count (I2C) and graph-based Image2Count models to counts of the test data from CosMx, t-CycIF and GeoMx datasets, averaged over all cross-validation runs (6 for CosMx, 10 for t-CycIF and GeoMx). Merged predictions were obtained by calculating the mean prediction over all cross-validation runs. DCA signifies models trained on raw data with correlation calculated from predicted counts and imputed count data using DCA (deep-count autoencoder). K hop stands for a k-hop subgraph created from a central cell, expanding the graph to neighboring nodes k times. For each image of the CosMx dataset, $6 \times 6 = 36$ subgraphs for a total of 1800 were created. For the t-CycIF dataset, $30 \times 30 = 900$ subgraphs were created for a total of 2700 subgraphs. Edges were determined via k-NN of spatial cell positions with number of neighbors set to 6. N Cells shows the (average) number of cells whose total expression was used to calculate correlation.

	Single Cell	1 hop	2 hop	3 hop	5 hop	8 hop	train size
N cells CosMx	1	7.68	21.28	42.95	109.69	266.01	483.71
ResNet	0.211 ± 0.015	0.354 ± 0.041	0.348 ± 0.018	0.324 ± 0.031	0.244 ± 0.030	0.241 ± 0.044	0.168 ± 0.010
ResNet merged	0.234	0.294	0.346	0.375	0.229	0.268	0.161
FFW I2C	0.186 ± 0.022	0.308 ± 0.039	0.304 ± 0.032	0.278 ± 0.022	0.215 ± 0.027	0.214 ± 0.018	0.171 ± 0.012
FFW I2C merged	0.188	0.317	0.288	0.296	0.236	0.228	0.193
GAT I2C	0.206 ± 0.021	0.287 ± 0.023	0.293 ± 0.021	0.313 ± 0.018	0.229 ± 0.024	0.226 ± 0.015	0.184 ± 0.017
GAT I2C merged	0.163	0.263	0.293	0.301	0.247	0.210	0.178
ResNet DCA	0.184 ± 0.013	0.346 ± 0.056	0.331 ± 0.030	0.321 ± 0.038	0.283 ± 0.019	0.240 ± 0.037	0.198 ± 0.015
ResNet DCA merged	0.197	0.299	0.346	0.393	0.258	0.265	0.184
FFW I2C DCA	0.159 ± 0.018	0.294 ± 0.023	0.280 ± 0.033	0.270 ± 0.020	0.238 ± 0.033	0.212 ± 0.018	0.191 ± 0.008
FFW I2C DCA merged	0.166	0.300	0.264	0.292	0.270	0.233	0.201
GAT I2C DCA	0.175 ± 0.016	0.281 ± 0.027	0.277 ± 0.022	0.301 ± 0.022	0.268 ± 0.030	0.216 ± 0.012	0.202 ± 0.020
GAT I2C DCA merged	0.148	0.260	0.297	0.283	0.302	0.198	0.187
N cells CycIF	1	7.78	19.85	38.25	95.88	234.84	438.54
ResNet	0.338 ± 0.017	0.342 ± 0.028	0.397 ± 0.023	0.398 ± 0.030	0.497 ± 0.029	0.515 ± 0.026	0.483 ± 0.031
ResNet merged	0.322	0.373	0.422	0.401	0.541	0.523	0.517
FFW I2C	0.251 ± 0.015	0.357 ± 0.042	0.402 ± 0.032	0.425 ± 0.028	0.477 ± 0.044	0.508 ± 0.023	0.484 ± 0.011
FFW I2C merged	0.320	0.342	0.409	0.430	0.466	0.513	0.481
GAT I2C	0.297 ± 0.024	0.343 ± 0.033	0.365 ± 0.031	0.410 ± 0.027	0.479 ± 0.051	0.493 ± 0.026	0.491 ± 0.026
GAT I2C merged	0.330	0.343	0.405	0.380	0.497	0.495	0.499
ResNet 2 Channels	0.258 ± 0.013	0.252 ± 0.016	0.347 ± 0.018	0.353 ± 0.020	0.386 ± 0.025	0.397 ± 0.015	0.418 ± 0.019
ResNet 2 Channels merged	0.263	0.278	0.354	0.333	0.392	0.381	0.425
FFW I2C 2 Channels	0.187 ± 0.013	0.262 ± 0.015	0.314 ± 0.024	0.333 ± 0.035	0.348 ± 0.022	0.375 ± 0.012	0.394 ± 0.028
FFW I2C 2 Channels merged	0.250	0.271	0.346	0.316	0.334	0.375	0.398
GAT I2C 2 Channels	0.229 ± 0.024	0.262 ± 0.020	0.309 ± 0.026	0.339 ± 0.026	0.363 ± 0.026	0.380 ± 0.030	0.411 ± 0.036
GAT I2C 2 Channels merged	0.252	0.291	0.317	0.364	0.396	0.339	0.425
ResNet no KM	0.270 ± 0.015	0.238 ± 0.018	0.301 ± 0.019	0.327 ± 0.016	0.421 ± 0.024	0.479 ± 0.028	0.505 ± 0.032
ResNet no KM merged	0.289	0.234	0.317	0.321	0.466	0.436	0.504
FFW I2C no KM	0.156 ± 0.018	0.240 ± 0.012	0.326 ± 0.023	0.370 ± 0.027	0.440 ± 0.024	0.455 ± 0.016	0.473 ± 0.022
FFW I2C no KM merged	0.220	0.334	0.331	0.332	0.491	0.478	0.478
GAT I2C no KM	0.177 ± 0.024	0.243 ± 0.025	0.329 ± 0.027	0.373 ± 0.020	0.449 ± 0.020	0.473 ± 0.025	0.476 ± 0.035
GAT I2C no KM merged	0.304	0.313	0.378	0.383	0.443	0.457	0.441
ResNet 8µm	0.236 ± 0.011	0.298 ± 0.017	0.319 ± 0.013	0.352 ± 0.017	0.417 ± 0.023	0.443 ± 0.026	0.464 ± 0.032
ResNet 8µm merged	0.232	0.287	0.345	0.396	0.374	0.399	0.449
FFW I2C 8µm	0.144 ± 0.008	0.290 ± 0.026	0.320 ± 0.028	0.348 ± 0.025	0.426 ± 0.037	0.424 ± 0.034	0.431 ± 0.022
FFW I2C 8µm merged	0.186	0.302	0.330	0.343	0.389	0.455	0.440
GAT I2C 8µm	0.204 ± 0.028	0.286 ± 0.042	0.323 ± 0.025	0.363 ± 0.034	0.419 ± 0.038	0.450 ± 0.034	0.441 ± 0.030
GAT I2C 8µm merged	0.216	0.292	0.301	0.349	0.451	0.469	0.424
ResNet 20µm	0.326 ± 0.026	0.317 ± 0.012	0.397 ± 0.022	0.423 ± 0.031	0.496 ± 0.029	0.518 ± 0.027	0.512 ± 0.024
ResNet 20µm merged	0.319	0.329	0.408	0.445	0.489	0.527	0.502
FFW I2C 20µm	0.189 ± 0.009	0.339 ± 0.031	0.389 ± 0.025	0.404 ± 0.029	0.504 ± 0.019	0.486 ± 0.037	0.487 ± 0.022
FFW I2C 20µm merged	0.238	0.370	0.421	0.436	0.445	0.526	0.482
GAT I2C 20µm	0.254 ± 0.016	0.325 ± 0.018	0.359 ± 0.031	0.380 ± 0.045	0.459 ± 0.039	0.469 ± 0.016	0.477 ± 0.016
GAT I2C 20µm merged	0.274	0.324	0.355	0.375	0.477	0.496	0.462
N cells GeoMx	-	-	-	-	-	-	269.83
ResNet 8µm	-	-	-	-	-	-	0.122 ± 0.025
ResNet 8µm merged	-	-	-	-	-	-	0.120
FFW I2C 8µm	-	-	-	-	-	-	0.160 ± 0.020
FFW I2C 8µm merged	-	-	-	-	-	-	0.175
GAT I2C 8µm	-	-	-	-	-	-	0.061 ± 0.029
GAT I2C 8µm merged	-	-	-	-	-	-	0.006
ResNet 12µm	-	-	-	-	-	-	0.189 ± 0.040
ResNet 12µm merged	-	-	-	-	-	-	0.151
FFW I2C 12µm	-	-	-	-	-	-	0.158 ± 0.024
FFW I2C 12µm merged	-	-	-	-	-	-	0.159
GAT I2C 12µm	-	-	-	-	-	-	0.181 ± 0.024
GAT I2C 12µm merged	-	-	-	-	-	-	0.189
ResNet 20µm	-	-	-	-	-	-	0.131 ± 0.013
ResNet 20µm merged	-	-	-	-	-	-	0.164
FFW I2C 20µm	-	-	-	-	-	-	0.162 ± 0.027
FFW I2C 20µm merged	-	-	-	-	-	-	0.165
GAT I2C 20µm	-	-	-	-	-	-	0.160 ± 0.017
GAT I2C 20µm merged	-	-	-	-	-	-	0.153

Table S8: Rand index adjusted for chance of clusters of predictions of linear evaluation head of the ResNet visual feature backbone, FFW Image2Count (I2C) and graph-based Image2Count models to clusters of counts of the test data from CosMx, t-CycIF and GeoMx datasets, averaged over all cross-validation runs (6 for CosMx, 10 for t-CycIF and GeoMx). Merged predictions were obtained by calculating the mean prediction over all cross-validation runs. DCA signifies models trained on raw data with correlation calculated from predicted counts and imputed count data using DCA (deep-count autoencoder). K hop stands for a k-hop subgraph created from a central cell, expanding the graph to neighboring nodes k times. For each image of the CosMx dataset, $6 \times 6 = 36$ subgraphs for a total of 1800 were created. For the t-CycIF dataset, $30 \times 30 = 900$ subgraphs were created for a total of 2700 subgraphs. Edges were determined via k-NN of spatial cell positions with number of neighbors set to 6. N Cells shows the (average) number of cells whose total expression was used to calculate correlation.

	Single Cell	1 hop	2 hop	3 hop	5 hop	8 hop	train size
N cells CosMx	1	7.68	21.28	42.95	109.69	266.01	483.71
ResNet	0.389 ± 0.015	0.504 ± 0.023	0.510 ± 0.010	0.471 ± 0.013	0.430 ± 0.019	0.433 ± 0.017	0.457 ± 0.020
ResNet merged	0.401	0.494	0.506	0.482	0.426	0.438	0.456
FFW I2C	0.358 ± 0.011	0.478 ± 0.017	0.494 ± 0.015	0.473 ± 0.013	0.424 ± 0.013	0.438 ± 0.007	0.460 ± 0.015
FFW I2C merged	0.366	0.500	0.494	0.482	0.424	0.447	0.490
GAT I2C	0.363 ± 0.013	0.478 ± 0.012	0.491 ± 0.010	0.488 ± 0.010	0.425 ± 0.010	0.440 ± 0.009	0.463 ± 0.012
GAT I2C merged	0.355	0.472	0.488	0.492	0.431	0.442	0.464
ResNet DCA	0.366 ± 0.013	0.499 ± 0.025	0.480 ± 0.013	0.472 ± 0.016	0.465 ± 0.013	0.436 ± 0.017	0.467 ± 0.019
ResNet DCA merged	0.378	0.493	0.477	0.494	0.455	0.447	0.461
FFW I2C DCA	0.338 ± 0.011	0.471 ± 0.015	0.462 ± 0.015	0.469 ± 0.014	0.451 ± 0.019	0.436 ± 0.008	0.466 ± 0.016
FFW I2C DCA merged	0.346	0.487	0.461	0.478	0.455	0.445	0.493
GAT I2C DCA	0.345 ± 0.013	0.469 ± 0.016	0.467 ± 0.012	0.480 ± 0.008	0.458 ± 0.008	0.439 ± 0.007	0.468 ± 0.012
GAT I2C DCA merged	0.337	0.464	0.473	0.485	0.465	0.442	0.467
N cells CycIF	1	7.78	19.85	38.25	95.88	234.84	438.54
ResNet	0.525 ± 0.006	0.485 ± 0.016	0.535 ± 0.013	0.556 ± 0.011	0.626 ± 0.015	0.650 ± 0.013	0.646 ± 0.013
ResNet merged	0.507	0.496	0.552	0.559	0.640	0.658	0.648
FFW I2C	0.484 ± 0.006	0.507 ± 0.023	0.545 ± 0.016	0.571 ± 0.012	0.621 ± 0.025	0.645 ± 0.013	0.651 ± 0.011
FFW I2C merged	0.523	0.524	0.549	0.587	0.622	0.654	0.653
GAT I2C	0.497 ± 0.013	0.499 ± 0.021	0.525 ± 0.022	0.567 ± 0.016	0.617 ± 0.028	0.638 ± 0.015	0.656 ± 0.012
GAT I2C merged	0.540	0.513	0.548	0.574	0.638	0.644	0.658
ResNet 2 Channels	0.446 ± 0.009	0.409 ± 0.011	0.480 ± 0.009	0.514 ± 0.013	0.551 ± 0.014	0.577 ± 0.010	0.601 ± 0.013
ResNet 2 Channels merged	0.442	0.420	0.488	0.504	0.555	0.569	0.610
FFW I2C 2 Channels	0.412 ± 0.009	0.423 ± 0.010	0.466 ± 0.016	0.502 ± 0.021	0.525 ± 0.012	0.554 ± 0.008	0.580 ± 0.021
FFW I2C 2 Channels merged	0.454	0.435	0.486	0.506	0.527	0.549	0.579
GAT I2C 2 Channels	0.439 ± 0.015	0.425 ± 0.014	0.472 ± 0.018	0.511 ± 0.018	0.532 ± 0.015	0.559 ± 0.017	0.586 ± 0.021
GAT I2C 2 Channels merged	0.471	0.455	0.481	0.526	0.559	0.540	0.606
ResNet no KM	0.432 ± 0.007	0.383 ± 0.015	0.446 ± 0.014	0.478 ± 0.013	0.560 ± 0.008	0.615 ± 0.017	0.633 ± 0.016
ResNet no KM merged	0.431	0.392	0.453	0.471	0.576	0.603	0.631
FFW I2C no KM	0.332 ± 0.013	0.364 ± 0.017	0.451 ± 0.018	0.508 ± 0.015	0.569 ± 0.014	0.601 ± 0.012	0.627 ± 0.012
FFW I2C no KM merged	0.409	0.466	0.484	0.524	0.614	0.627	0.646
GAT I2C no KM	0.341 ± 0.026	0.376 ± 0.022	0.458 ± 0.017	0.513 ± 0.012	0.578 ± 0.014	0.614 ± 0.018	0.630 ± 0.019
GAT I2C no KM merged	0.473	0.452	0.517	0.552	0.589	0.614	0.619
ResNet 8µm	0.392 ± 0.008	0.425 ± 0.008	0.465 ± 0.006	0.501 ± 0.012	0.568 ± 0.010	0.602 ± 0.015	0.628 ± 0.010
ResNet 8µm merged	0.399	0.420	0.483	0.520	0.559	0.581	0.627
FFW I2C 8µm	0.354 ± 0.009	0.424 ± 0.018	0.465 ± 0.018	0.512 ± 0.011	0.565 ± 0.021	0.589 ± 0.015	0.604 ± 0.017
FFW I2C 8µm merged	0.391	0.448	0.482	0.515	0.550	0.600	0.616
GAT I2C 8µm	0.389 ± 0.027	0.424 ± 0.036	0.466 ± 0.020	0.509 ± 0.021	0.566 ± 0.029	0.601 ± 0.024	0.611 ± 0.024
GAT I2C 8µm merged	0.407	0.449	0.464	0.511	0.589	0.607	0.609
ResNet 20µm	0.491 ± 0.006	0.464 ± 0.014	0.521 ± 0.016	0.558 ± 0.019	0.615 ± 0.021	0.644 ± 0.017	0.659 ± 0.011
ResNet 20µm merged	0.491	0.478	0.518	0.560	0.606	0.651	0.669
FFW I2C 20µm	0.433 ± 0.006	0.476 ± 0.017	0.525 ± 0.011	0.545 ± 0.014	0.621 ± 0.016	0.631 ± 0.021	0.648 ± 0.012
FFW I2C 20µm merged	0.463	0.515	0.542	0.561	0.592	0.658	0.652
GAT I2C 20µm	0.457 ± 0.013	0.466 ± 0.010	0.508 ± 0.018	0.534 ± 0.016	0.595 ± 0.020	0.624 ± 0.016	0.639 ± 0.012
GAT I2C 20µm merged	0.474	0.488	0.529	0.545	0.621	0.631	0.638
N cells GeoMx	-	-	-	-	-	-	269.83
ResNet 8µm	-	-	-	-	-	-	0.222 ± 0.030
ResNet 8µm merged	-	-	-	-	-	-	0.228
FFW I2C 8µm	-	-	-	-	-	-	0.286 ± 0.019
FFW I2C 8µm merged	-	-	-	-	-	-	0.317
GAT I2C 8µm	-	-	-	-	-	-	0.113 ± 0.051
GAT I2C 8µm merged	-	-	-	-	-	-	0.061
ResNet 12µm	-	-	-	-	-	-	0.318 ± 0.034
ResNet 12µm merged	-	-	-	-	-	-	0.289
FFW I2C 12µm	-	-	-	-	-	-	0.287 ± 0.029
FFW I2C 12µm merged	-	-	-	-	-	-	0.305
GAT I2C 12µm	-	-	-	-	-	-	0.316 ± 0.024
GAT I2C 12µm merged	-	-	-	-	-	-	0.339
ResNet 20µm	-	-	-	-	-	-	0.255 ± 0.017
ResNet 20µm merged	-	-	-	-	-	-	0.289
FFW I2C 20µm	-	-	-	-	-	-	0.289 ± 0.035
FFW I2C 20µm merged	-	-	-	-	-	-	0.310
GAT I2C 20µm	-	-	-	-	-	-	0.296 ± 0.018
GAT I2C 20µm merged	-	-	-	-	-	-	0.289

Table S9: Normalized Mutual Information of clusters of predictions of linear evaluation head of the ResNet visual feature backbone, FFW Image2Count (I2C) and graph-based Image2Count models to clusters of counts of the test data from CosMx, t-CycIF and GeoMx datasets, averaged over all cross-validation runs (6 for CosMx, 10 for t-CycIF and GeoMx). Merged predictions were obtained by calculating the mean prediction over all cross-validation runs. DCA signifies models trained on raw data with correlation calculated from predicted counts and imputed count data using DCA (deep-count autoencoder). K hop stands for a k-hop subgraph created from a central cell, expanding the graph to neighboring nodes k times. For each image of the CosMx dataset, $6 \times 6 = 36$ subgraphs for a total of 1800 were created. For the t-CycIF dataset, $30 \times 30 = 900$ subgraphs were created for a total of 2700 subgraphs. Edges were determined via k-NN of spatial cell positions with number of neighbors set to 6. N Cells shows the (average) number of cells whose total expression was used to calculate correlation.

	Single Cell	1 hop	2 hop	3 hop	5 hop	8 hop	train size
N cells CosMx	1	7.68	21.28	42.95	109.69	266.01	483.71
ResNet	0.054 ± 0.036	0.053 ± 0.009	0.050 ± 0.013	0.044 ± 0.022	0.040 ± 0.020	0.038 ± 0.020	0.035 ± 0.019
ResNet merged	0.087	0.075	0.064	0.080	0.049	0.053	0.057
FFW I2C	0.040 ± 0.027	0.026 ± 0.009	0.033 ± 0.022	0.029 ± 0.014	0.019 ± 0.010	0.033 ± 0.021	0.031 ± 0.009
FFW I2C merged	0.022	0.013	0.012	0.014	0.007	0.017	0.009
GAT I2C	0.040 ± 0.026	0.028 ± 0.021	0.023 ± 0.017	0.034 ± 0.023	0.029 ± 0.018	0.027 ± 0.010	0.029 ± 0.006
GAT I2C merged	0.020	0.016	0.041	0.054	0.037	0.021	0.029
ResNet DCA	0.064 ± 0.040	0.043 ± 0.029	0.030 ± 0.007	0.027 ± 0.013	0.027 ± 0.010	0.030 ± 0.016	0.023 ± 0.015
ResNet DCA merged	0.076	0.056	0.032	0.021	0.018	0.027	0.016
FFW I2C DCA	0.044 ± 0.026	0.036 ± 0.025	0.045 ± 0.027	0.036 ± 0.032	0.030 ± 0.017	0.033 ± 0.021	0.029 ± 0.008
FFW I2C DCA merged	0.041	0.020	0.038	0.032	0.000	0.017	0.019
GAT I2C DCA	0.050 ± 0.032	0.040 ± 0.023	0.024 ± 0.016	0.038 ± 0.022	0.045 ± 0.022	0.029 ± 0.013	0.029 ± 0.006
GAT I2C DCA merged	0.052	0.029	0.044	0.053	0.050	0.039	0.020

Table S10: Coverage of top 5 CollecTRI transcription factors enriched in clusters of predictions of linear evaluation head of the ResNet visual feature backbone, FFW Image2Count (I2C) and graph-based Image2Count models to transcription factors enriched for clusters of counts of the test data from the CosMx datasets, averaged over all cross-validation runs (6). Merged predictions were obtained by calculating the mean prediction over all cross-validation runs. DCA signifies models trained on raw data with coverage calculated from predicted counts and imputed count data using DCA (deep-count autoencoder). K hop stands for a k-hop subgraph created from a central cell, expanding the graph to neighboring nodes k times. For each image of the CosMx dataset, $6 \times 6 = 36$ subgraphs for a total of 1800 were created. Edges were determined via k-NN of spatial cell positions with number of neighbors set to 6. N Cells shows the (average) number of cells whose total expression was used to calculate correlation. Coverage is how many of the top 5 pathways enriched per single-cell clustering of the predicted data are found in the pathways enriched in the test data.

	Single Cell	1 hop	2 hop	3 hop	5 hop	8 hop	train size
N cells CosMx	1	7.68	21.28	42.95	109.69	266.01	483.71
ResNet	0.288 ± 0.104	0.265 ± 0.088	0.321 ± 0.048	0.318 ± 0.047	0.224 ± 0.041	0.224 ± 0.035	0.231 ± 0.022
ResNet merged	0.269	0.279	0.308	0.321	0.308	0.269	0.231
FFW I2C	0.340 ± 0.084	0.408 ± 0.125	0.378 ± 0.084	0.327 ± 0.121	0.276 ± 0.116	0.290 ± 0.087	0.329 ± 0.093
FFW I2C merged	0.385	0.439	0.500	0.404	0.423	0.423	0.385
GAT I2C	0.397 ± 0.065	0.394 ± 0.070	0.436 ± 0.053	0.401 ± 0.075	0.333 ± 0.076	0.361 ± 0.058	0.370 ± 0.085
GAT I2C merged	0.423	0.401	0.423	0.442	0.500	0.308	0.385
ResNet DCA	0.327 ± 0.076	0.301 ± 0.052	0.276 ± 0.060	0.306 ± 0.065	0.298 ± 0.056	0.285 ± 0.065	0.338 ± 0.042
ResNet DCA merged	0.308	0.269	0.231	0.269	0.269	0.404	0.321
FFW I2C DCA	0.205 ± 0.088	0.365 ± 0.094	0.297 ± 0.094	0.287 ± 0.088	0.343 ± 0.108	0.337 ± 0.082	0.298 ± 0.056
FFW I2C DCA merged	0.231	0.385	0.308	0.346	0.385	0.423	0.308
GAT I2C DCA	0.282 ± 0.065	0.368 ± 0.080	0.365 ± 0.073	0.339 ± 0.079	0.364 ± 0.059	0.422 ± 0.067	0.365 ± 0.104
GAT I2C DCA merged	0.269	0.308	0.346	0.362	0.442	0.423	0.282

Table S11: Coverage of top 5 PROGENy pathways enriched in clusters of predictions of linear evaluation head of the ResNet visual feature backbone, FFW Image2Count (I2C) and graph-based Image2Count models to pathways enriched for clusters of counts of the test data from the CosMx datasets, averaged over all cross-validation runs (6). Merged predictions were obtained by calculating the mean prediction over all cross-validation runs. DCA signifies models trained on raw data with coverage calculated from predicted counts and imputed count data using DCA (deep-count autoencoder). K hop stands for a k-hop subgraph created from a central cell, expanding the graph to neighboring nodes k times. For each image of the CosMx dataset, $6 \times 6 = 36$ subgraphs for a total of 1800 were created. Edges were determined via k-NN of spatial cell positions with number of neighbors set to 6. N Cells shows the (average) number of cells whose total expression was used to calculate correlation. Coverage is how many of the top 5 pathways enriched per single-cell clustering of the predicted data are found in the pathways enriched in the test data.

	Single Cell	1 hop	2 hop	3 hop	5 hop	8 hop	train size
N cells CosMx	1	7.68	21.28	42.95	109.69	266.01	483.71
ResNet	0.273 ± 0.062	0.278 ± 0.096	0.248 ± 0.082	0.258 ± 0.073	0.207 ± 0.081	0.162 ± 0.098	0.151 ± 0.084
ResNet merged	0.260	0.350	0.267	0.216	0.220	0.161	0.130
FFW I2C	0.223 ± 0.071	0.227 ± 0.092	0.224 ± 0.090	0.213 ± 0.064	0.177 ± 0.064	0.131 ± 0.053	0.111 ± 0.049
FFW I2C merged	0.390	0.405	0.321	0.385	0.196	0.149	0.090
GAT I2C	0.275 ± 0.085	0.263 ± 0.096	0.233 ± 0.088	0.249 ± 0.083	0.179 ± 0.092	0.134 ± 0.046	0.135 ± 0.065
GAT I2C merged	0.369	0.273	0.277	0.295	0.201	0.162	0.196
ResNet DCA	0.283 ± 0.068	0.297 ± 0.082	0.243 ± 0.086	0.179 ± 0.092	0.190 ± 0.081	0.159 ± 0.065	0.144 ± 0.047
ResNet DCA merged	0.252	0.334	0.243	0.210	0.194	0.175	0.199
FFW I2C DCA	0.212 ± 0.044	0.212 ± 0.063	0.244 ± 0.076	0.159 ± 0.064	0.156 ± 0.067	0.132 ± 0.060	0.114 ± 0.026
FFW I2C DCA merged	0.307	0.316	0.348	0.205	0.229	0.133	0.107
GAT I2C DCA	0.230 ± 0.068	0.215 ± 0.078	0.196 ± 0.088	0.178 ± 0.073	0.158 ± 0.085	0.167 ± 0.064	0.145 ± 0.061
GAT I2C DCA merged	0.292	0.218	0.225	0.240	0.252	0.167	0.215

Table S12: Coverage of top 5 Hallmark pathways enriched in clusters of predictions of linear evaluation head of the ResNet visual feature backbone, FFW Image2Count (I2C) and graph-based Image2Count models to pathways enriched for clusters of counts of the test data from the CosMx datasets, averaged over all cross-validation runs (6). Merged predictions were obtained by calculating the mean prediction over all cross-validation runs. DCA signifies models trained on raw data with coverage calculated from predicted counts and imputed count data using DCA (deep-count autoencoder). K hop stands for a k-hop subgraph created from a central cell, expanding the graph to neighboring nodes k times. For each image of the CosMx dataset, $6 \times 6 = 36$ subgraphs for a total of 1800 were created. Edges were determined via k-NN of spatial cell positions with number of neighbors set to 6. N Cells shows the (average) number of cells whose total expression was used to calculate correlation. Coverage is how many of the top 5 pathways enriched per single-cell clustering of the predicted data are found in the pathways enriched in the test data.

	Single Cell	1 hop	2 hop	3 hop	5 hop	8 hop	train size
N cells CosMx	1	7.68	21.28	42.95	109.69	266.01	483.71
ResNet	0.184 ± 0.057	0.144 ± 0.022	0.154 ± 0.013	0.086 ± 0.036	0.077 ± 0.027	0.078 ± 0.019	0.064 ± 0.033
ResNet merged	0.166	0.162	0.158	0.137	0.063	0.036	0.070
FFW I2C	0.092 ± 0.048	0.086 ± 0.035	0.096 ± 0.053	0.070 ± 0.042	0.047 ± 0.027	0.057 ± 0.025	0.042 ± 0.017
FFW I2C merged	0.117	0.070	0.140	0.072	0.091	0.085	0.063
GAT I2C	0.106 ± 0.037	0.091 ± 0.049	0.107 ± 0.050	0.083 ± 0.043	0.079 ± 0.040	0.070 ± 0.025	0.061 ± 0.021
GAT I2C merged	0.153	0.080	0.176	0.128	0.144	0.069	0.091
ResNet DCA	0.109 ± 0.049	0.125 ± 0.026	0.123 ± 0.075	0.148 ± 0.056	0.098 ± 0.021	0.082 ± 0.021	0.061 ± 0.020
ResNet DCA merged	0.121	0.220	0.109	0.141	0.096	0.103	0.052
FFW I2C DCA	0.078 ± 0.049	0.064 ± 0.028	0.081 ± 0.042	0.094 ± 0.056	0.079 ± 0.035	0.053 ± 0.022	0.057 ± 0.015
FFW I2C DCA merged	0.087	0.079	0.110	0.093	0.053	0.066	0.060
GAT I2C DCA	0.097 ± 0.021	0.084 ± 0.045	0.112 ± 0.063	0.125 ± 0.073	0.089 ± 0.033	0.073 ± 0.030	0.066 ± 0.032
GAT I2C DCA merged	0.090	0.163	0.242	0.164	0.128	0.091	0.110

Table S13: Coverage of top 5 Reactome pathways enriched in clusters of predictions of linear evaluation head of the ResNet visual feature backbone, FFW Image2Count (I2C) and graph-based Image2Count models to pathways enriched for clusters of counts of the test data from the CosMx datasets, averaged over all cross-validation runs (6). Merged predictions were obtained by calculating the mean prediction over all cross-validation runs. DCA signifies models trained on raw data with coverage calculated from predicted counts and imputed count data using DCA (deep-count autoencoder). K hop stands for a k-hop subgraph created from a central cell, expanding the graph to neighboring nodes k times. For each image of the CosMx dataset, $6 \times 6 = 36$ subgraphs for a total of 1800 were created. Edges were determined via k-NN of spatial cell positions with number of neighbors set to 6. N Cells shows the (average) number of cells whose total expression was used to calculate correlation. Coverage is how many of the top 5 pathways enriched per single-cell clustering of the predicted data are found in the pathways enriched in the test data.

	Single Cell	1 hop	2 hop	3 hop	5 hop	8 hop	train size
N cells CosMx	1	7.68	21.28	42.95	109.69	266.01	483.71
ResNet	0.157 ± 0.054	0.226 ± 0.054	0.254 ± 0.111	0.179 ± 0.055	0.180 ± 0.070	0.150 ± 0.039	0.130 ± 0.067
ResNet merged	0.115	0.244	0.175	0.266	0.236	0.145	0.126
FFW I2C	0.175 ± 0.058	0.147 ± 0.085	0.161 ± 0.061	0.145 ± 0.063	0.155 ± 0.051	0.143 ± 0.045	0.120 ± 0.041
FFW I2C merged	0.211	0.230	0.217	0.233	0.193	0.154	0.226
GAT I2C	0.173 ± 0.029	0.162 ± 0.073	0.224 ± 0.117	0.145 ± 0.033	0.160 ± 0.080	0.149 ± 0.058	0.149 ± 0.048
GAT I2C merged	0.183	0.240	0.276	0.290	0.192	0.182	0.204
ResNet DCA	0.104 ± 0.026	0.198 ± 0.064	0.238 ± 0.082	0.175 ± 0.048	0.138 ± 0.070	0.141 ± 0.048	0.104 ± 0.056
ResNet DCA merged	0.078	0.159	0.233	0.287	0.177	0.151	0.113
FFW I2C DCA	0.133 ± 0.062	0.135 ± 0.045	0.145 ± 0.024	0.127 ± 0.035	0.133 ± 0.041	0.123 ± 0.032	0.089 ± 0.031
FFW I2C DCA merged	0.181	0.123	0.168	0.169	0.121	0.152	0.144
GAT I2C DCA	0.139 ± 0.028	0.150 ± 0.073	0.174 ± 0.080	0.138 ± 0.090	0.115 ± 0.062	0.134 ± 0.040	0.106 ± 0.017
GAT I2C DCA merged	0.177	0.157	0.265	0.223	0.166	0.142	0.137

Table S14: Coverage of top 5 Kegg pathways enriched in clusters of predictions of linear evaluation head of the ResNet visual feature backbone, FFW Image2Count (I2C) and graph-based Image2Count models to pathways enriched for clusters of counts of the test data from the CosMx datasets, averaged over all cross-validation runs (6). Merged predictions were obtained by calculating the mean prediction over all cross-validation runs. DCA signifies models trained on raw data with correlation calculated from predicted counts and imputed count data using DCA (deep-count autoencoder). K hop stands for a k-hop subgraph created from a central cell, expanding the graph to neighboring nodes k times. For each image of the CosMx dataset, $6 \times 6 = 36$ subgraphs for a total of 1800 were created. Edges were determined via k-NN of spatial cell positions with number of neighbors set to 6. N Cells shows the (average) number of cells whose total expression was used to calculate correlation. Coverage is how many of the top 5 pathways enriched per single-cell clustering of the predicted data are found in the pathways enriched in the test data.

Model	Top 1	Top 3	Top 5
ResNet	0.082 ± 0.063	0.207 ± 0.093	0.233 ± 0.091
ResNet merged	0.101	0.235	0.218
FFW I2C	0.043 ± 0.052	0.132 ± 0.069	0.176 ± 0.055
FFW I2C merged	0.056	0.165	0.186
GAT I2C	0.050 ± 0.077	0.121 ± 0.076	0.158 ± 0.077
GAT I2C merged	0.146	0.276	0.252
ResNet DCA	0.163 ± 0.088	0.234 ± 0.085	0.244 ± 0.080
ResNet merged DCA	0.241	0.262	0.216
FFW I2C DCA	0.041 ± 0.034	0.154 ± 0.052	0.178 ± 0.067
FFW I2C merged DCA	0.140	0.162	0.212
GAT I2C DCA	0.071 ± 0.092	0.167 ± 0.097	0.195 ± 0.110
GAT I2C merged DCA	0.100	0.243	0.258

Table S15: Coverage of top 5, top 3 and top 1 lung cell types enriched in clusters of predictions of linear evaluation head of the ResNet visual feature backbone, FFW Image2Count (I2C) and graph-based Image2Count models to cell types enriched for clusters of counts of the test data from the CosMx datasets, averaged over all cross-validation runs (6). Merged predictions were obtained by calculating the mean prediction over all cross-validation runs. DCA signifies models trained on raw data with correlation calculated from predicted counts and imputed count data using DCA (deep-count autoencoder). Coverage is how many of the top n cell types enriched per single-cell clustering of the predicted data are found in the cell types enriched in the test data.

K-Hop	Identified Transcription Factors
1	• ETV4
2	• EWSR1<br>• SOX2
3	• EWSR1<br>• TAL1<br>• PITX2<br>• NFIA
5	• EWSR1<br>• TAL1<br>• STAT1
8	• STAT2<br>• EWSR1
11	• STAT2<br>• EWSR1<br>• PITX2<br>• JUND<br>• NFIA
sc	• NFATC1<br>• PITX2

Table S16: Identified CollecTRI Transcription Factors per niche sizes (predicted counts resulted in: 11 clusters for 1-hop; 12 for 2-hop; 11 for 3-hop; 10 for 5-hop; 13 for 8-hop; 12 for 11-hop; 20 for single cells, true counts in: 9 clusters for 1-hop; 11 for 2-hop; 13 for 3-hop; 20 for 5-hop; 28 for 8-hop; 36 for 11-hop; 14 for single cells).

K-Hop	Identified PROGENy Pathways
1	• TGFb<br>• EGFR<br>• TNFa<br>• NFkB<br>• JAK-STAT
2	• TGFb<br>• EGFR<br>• TNFa<br>• NFkB<br>• JAK-STAT<br>• MAPK

K-Hop	Identified PROGENy Pathways
**3**	• TGFb<br>• WNT<br>• TNFa<br>• NFkB<br>• JAK-STAT<br>• MAPK<br>• EGFR
**5**	• NFkB<br>• TGFb<br>• TNFa<br>• Hypoxia<br>• JAK-STAT<br>• MAPK<br>• WNT<br>• EGFR
**8**	• MAPK<br>• NFkB<br>• JAK-STAT<br>• TNFa<br>• TGFb
**11**	• TGFb<br>• TNFa<br>• NFkB<br>• JAK-STAT<br>• MAPK<br>• EGFR
**sc**	• Hypoxia<br>• TGFb<br>• TNFa<br>• EGFR<br>• NFkB<br>• JAK-STAT<br>• MAPK<br>• PI3K

Table S17: Identified PROGENy Pathway Activities per niche sizes (predicted counts resulted in: 11 clusters for 1-hop; 12 for 2-hop; 11 for 3-hop; 10 for 5-hop; 13 for 8-hop; 12 for 11-hop; 20 for single cells, true counts in: 9 clusters for 1-hop; 11 for 2-hop; 13 for 3-hop; 20 for 5-hop; 28 for 8-hop; 36 for 11-hop; 14 for single cells).

K-Hop	Identified Hallmark Pathways
**1**	• UV_RESPONSE_UP<br>• ESTROGEN_RESPONSE_LATE<br>• INTERFERON_ALPHA_RESPONSE<br>• EPITHELIAL_MESENCHYMAL_TRANSITION<br>• P53_PATHWAY<br>• ADIPOGENESIS<br>• UV_RESPONSE_DN<br>• MYOGENESIS<br>• PI3K_AKT_MTOR_SIGNALING
**2**	• UV_RESPONSE_UP<br>• ESTROGEN_RESPONSE_LATE<br>• ADIPOGENESIS<br>• INTERFERON_ALPHA_RESPONSE<br>• EPITHELIAL_MESENCHYMAL_TRANSITION<br>• PI3K_AKT_MTOR_SIGNALING<br>• MYOGENESIS<br>• P53_PATHWAY<br>• IL6_JAK_STAT3_SIGNALING<br>• TNFA_SIGNALING_VIA_NFKB<br>• INFLAMMATORY_RESPONSE<br>• UV_RESPONSE_DN
**3**	• UV_RESPONSE_UP<br>• COMPLEMENT<br>• XENOBIOTIC_METABOLISM<br>• ESTROGEN_RESPONSE_LATE<br>• ADIPOGENESIS<br>• INTERFERON_ALPHA_RESPONSE<br>• EPITHELIAL_MESENCHYMAL_TRANSITION<br>• MYOGENESIS<br>• TNFA_SIGNALING_VIA_NFKB<br>• INFLAMMATORY_RESPONSE<br>• EPITHELIAL_MESENCHYMAL_TRANSITION<br>• TNFA_SIGNALING_VIA_NFKB
**5**	• UV_RESPONSE_UP<br>• XENOBIOTIC_METABOLISM<br>• INTERFERON_ALPHA_RESPONSE<br>• MYOGENESIS<br>• INFLAMMATORY_RESPONSE<br>• EPITHELIAL_MESENCHYMAL_TRANSITION<br>• TNFA_SIGNALING_VIA_NFKB<br>• TGF_BETA_SIGNALING<br>• INFLAMMATORY_RESPONSE
**8**	• UV_RESPONSE_UP<br>• XENOBIOTIC_METABOLISM<br>• INFLAMMATORY_RESPONSE<br>• INTERFERON_ALPHA_RESPONSE<br>• TGF_BETA_SIGNALING<br>• MYOGENESIS<br>• TNFA_SIGNALING_VIA_NFKB

K-Hop	Identified Hallmark Pathways
**11**	• UV_RESPONSE_UP<br>• XENOBIOTIC_METABOLISM<br>• INTERFERON_ALPHA_RESPONSE<br>• INFLAMMATORY_RESPONSE<br>• TNFA_SIGNALING_VIA_NFKB<br>• IL6_JAK_STAT3_SIGNALING<br>• UV_RESPONSE_DN<br>• MYOGENESIS<br>• P53_PATHWAY
**sc**	• UV_RESPONSE_UP<br>• UV_RESPONSE_DN<br>• INTERFERON_ALPHA_RESPONSE<br>• EPITHELIAL_MESENCHYMAL_TRANSITION<br>• ANGIOGENESIS<br>• MYOGENESIS<br>• P53_PATHWAY<br>• INTERFERON_GAMMA_RESPONSE<br>• ESTROGEN_RESPONSE_EARLY<br>• ESTROGEN_RESPONSE_LATE<br>• ALLOGRAFT_REJECTION<br>• MYOGENESIS<br>• KRAS_SIGNALING_DN<br>• IL6_JAK_STAT3_SIGNALING

Table S18: Identified MSigDB Hallmark Pathways per niche sizes (predicted counts resulted in: 11 clusters for 1-hop; 12 for 2-hop; 11 for 3-hop; 10 for 5-hop; 13 for 8-hop; 12 for 11-hop; 20 for single cells, true counts in: 9 clusters for 1-hop; 11 for 2-hop; 13 for 3-hop; 20 for 5-hop; 28 for 8-hop; 36 for 11-hop; 14 for single cells).

K-Hop	Identified Reactome Pathways
**1**	• REACTOME_CELL_CELL_COMMUNICATION<br>• REACTOME_REGULATION_OF_INSULIN_LIKE_GROWTH_FACTOR_IGF_TRANSPORT_AND_UPTAKE_BY_INSULIN_LIKE_GROWTH_FACTOR_BINDING_PROTEINS_IGFBPS<br>• REACTOME_TRANSCRIPTIONAL_REGULATION_BY_RUNX2<br>• REACTOME_INTERFERON_GAMMA_SIGNALING<br>• REACTOME_COMPLEMENT_CASCADE
**2**	• REACTOME_CELL_CELL_COMMUNICATION<br>• REACTOME_FORMATION_OF_THE_CORNIFIED_ENVELOPE<br>• REACTOME_COLLAGEN_CHAIN_TRIMERIZATION<br>• REACTOME_COMPLEMENT_CASCADE<br>• REACTOME_CO_INHIBITION_BY_PD_1<br>• REACTOME_INTERLEUKIN_4_AND_INTERLEUKIN_13_SIGNALING<br>• REACTOME_COLLAGEN_BIOSYNTHESIS_AND_MODIFYING_ENZYMES<br>• REACTOME_REGULATION_OF_INSULIN_LIKE_GROWTH_FACTOR_IGF_TRANSPORT_AND_UPTAKE_BY_INSULIN_LIKE_GROWTH_FACTOR_BINDING_PROTEINS_IGFBPS<br>• REACTOME_DEVELOPMENTAL_CELL_LINEAGES

K-Hop	Identified Reactome Pathways
**3**	• REACTOME_ANTIVIRAL_MECHANISM_BY_IFN_STIMULATED_GENES<br>• REACTOME_CELL_CELL_COMMUNICATION<br>• REACTOME_COMPLEMENT_CASCADE<br>• REACTOME_REGULATION_OF_INSULIN_LIKE_GROWTH_FACTOR_IGF_TRANSPORT_AND_UPTAKE_BY_INSULIN_LIKE_GROWTH_FACTOR_BINDING_PROTEINS_IGFBPS<br>• REACTOME_INTERLEUKIN_3_INTERLEUKIN_5_AND_GM_CSF_SIGNALING<br>• REACTOME_COLLAGEN_CHAIN_TRIMERIZATION<br>• REACTOME_COLLAGEN_BIOSYNTHESIS_AND_MODIFYING_ENZYMES<br>• REACTOME_DEVELOPMENTAL_CELL_LINEAGES
**5**	• REACTOME_CELL_CELL_COMMUNICATION<br>• REACTOME_REGULATION_OF_INSULIN_LIKE_GROWTH_FACTOR_IGF_TRANSPORT_AND_UPTAKE_BY_INSULIN_LIKE_GROWTH_FACTOR_BINDING_PROTEINS_IGFBPS<br>• REACTOME_ANTIVIRAL_MECHANISM_BY_IFN_STIMULATED_GENES<br>• REACTOME_INTERLEUKIN_10_SIGNALING<br>• REACTOME_INTERLEUKIN_2_FAMILY_SIGNALING<br>• REACTOME_SYNDECAN_INTERACTIONS<br>• REACTOME_NON_INTEGRIN_MEMBRANE_ECM_INTERACTIONS<br>• REACTOME_CELL_CELL_COMMUNICATION<br>• REACTOME_INTERLEUKIN_3_INTERLEUKIN_5_AND_GM_CSF_SIGNALING<br>• REACTOME_COLLAGEN_CHAIN_TRIMERIZATION<br>• REACTOME_COLLAGEN_BIOSYNTHESIS_AND_MODIFYING_ENZYMES
**8**	• REACTOME_REGULATION_OF_INSULIN_LIKE_GROWTH_FACTOR_IGF_TRANSPORT_AND_UPTAKE_BY_INSULIN_LIKE_GROWTH_FACTOR_BINDING_PROTEINS_IGFBPS<br>• REACTOME_INTERLEUKIN_3_INTERLEUKIN_5_AND_GM_CSF_SIGNALING<br>• REACTOME_ANTIVIRAL_MECHANISM_BY_IFN_STIMULATED_GENES<br>• REACTOME_MYD88_INDEPENDENT_TLR4_CASCADE<br>• REACTOME_INTERLEUKIN_2_FAMILY_SIGNALING<br>• REACTOME_NON_INTEGRIN_MEMBRANE_ECM_INTERACTIONS
**11**	• REACTOME_ANTIVIRAL_MECHANISM_BY_IFN_STIMULATED_GENES<br>• REACTOME_INTERLEUKIN_2_FAMILY_SIGNALING<br>• REACTOME_REGULATION_OF_INSULIN_LIKE_GROWTH_FACTOR_IGF_TRANSPORT_AND_UPTAKE_BY_INSULIN_LIKE_GROWTH_FACTOR_BINDING_PROTEINS_IGFBPS<br>• REACTOME_INTERLEUKIN_3_INTERLEUKIN_5_AND_GM_CSF_SIGNALING<br>• REACTOME_CELL_CELL_COMMUNICATION<br>• REACTOME_RSV_HOST_INTERACTIONS<br>• REACTOME_NON_INTEGRIN_MEMBRANE_ECM_INTERACTIONS

K-Hop	Identified Reactome Pathways
sc	• REACTOME_INTERFERON_GAMMA_SIGNALING<br>• REACTOME_FORMATION_OF_THE_CORNIFIED_ENVELOPE<br>• REACTOME_CLASS_A_1_RHODOPSIN_LIKE_RECEPTORS<br>• REACTOME_KERATINIZATION<br>• REACTOME_SIGNALING_BY_TGFBR3<br>• REACTOME_MET_ACTIVATES_PTK2_SIGNALING<br>• REACTOME_INTERLEUKIN_6_FAMILY_SIGNALING<br>• REACTOME_IMMUNOREGULATORY_INTERACTIONS_BETWEEN_A_LYMPHOID_AND_A_NON_LYMPHOID_CELL<br>• REACTOME_INTERLEUKIN_20_FAMILY_SIGNALING<br>• REACTOME_RESPIRATORY_SYNCYTIAL_VIRUS_INFECTION_PATHWAY<br>• REACTOME_RSV_HOST_INTERACTIONS

Table S19: Identified Reactome Pathways per niche sizes (predicted counts resulted in: 11 clusters for 1-hop; 12 for 2-hop; 11 for 3-hop; 10 for 5-hop; 13 for 8-hop; 12 for 11-hop; 20 for single cells, true counts in: 9 clusters for 1-hop; 11 for 2-hop; 13 for 3-hop; 20 for 5-hop; 28 for 8-hop; 36 for 11-hop; 14 for single cells).

K-Hop	Identified KEGG Pathways
1	• KEGG_GRAFT_VERSUS_HOST_DISEASE<br>• KEGG_AUTOIMMUNE_THYROID_DISEASE<br>• KEGG_ALLOGRAFT_REJECTION<br>• KEGG_BLADDER_CANCER<br>• KEGG_TYPE_I_DIABETES_MELLITUS<br>• KEGG_INTESTINAL_IMMUNE_NETWORK_FOR_IGA_PRODUCTION<br>• KEGG_HEMATOPOIETIC_CELL_LINEAGE<br>• KEGG_ALLOGRAFT_REJECTION<br>• KEGG_REGULATION_OF_ACTIN_CYTOSKELETON<br>• KEGG_LEISHMANIA_INFECTION<br>• KEGG_NATURAL_KILLER_CELL_MEDIATED_CYTOTOXICITY<br>• KEGG_HEMATOPOIETIC_CELL_LINEAGE
2	• KEGG_GRAFT_VERSUS_HOST_DISEASE<br>• KEGG_BLADDER_CANCER<br>• KEGG_INTESTINAL_IMMUNE_NETWORK_FOR_IGA_PRODUCTION<br>• KEGG_HEMATOPOIETIC_CELL_LINEAGE<br>• KEGG_ECM_RECEPTOR_INTERACTION<br>• KEGG_GRAFT_VERSUS_HOST_DISEASE<br>• KEGG_AUTOIMMUNE_THYROID_DISEASE<br>• KEGG_TOLL_LIKE_RECEPTOR_SIGNALING_PATHWAY<br>• KEGG_TYPE_I_DIABETES_MELLITUS<br>• KEGG_REGULATION_OF_ACTIN_CYTOSKELETON<br>• KEGG_HYPERTROPHIC_CARDIOMYOPATHY_HCM<br>• KEGG_CELL_ADHESION_MOLECULES_CAMS<br>• KEGG_NATURAL_KILLER_CELL_MEDIATED_CYTOTOXICITY<br>• KEGG_ECM_RECEPTOR_INTERACTION

K-Hop	Identified KEGG Pathways
3	• KEGG_NOD_LIKE_RECEPTOR_SIGNALING_PATHWAY<br>• KEGG_TOLL_LIKE_RECEPTOR_SIGNALING_PATHWAY<br>• KEGG_CELL_ADHESION_MOLECULES_CAMS<br>• KEGG_HEMATOPOIETIC_CELL_LINEAGE<br>• KEGG_LEUKOCYTE_TRANSENDOTHELIAL_MIGRATION<br>• KEGG_AUTOIMMUNE_THYROID_DISEASE<br>• KEGG_ALLOGRAFT_REJECTION<br>• KEGG_DILATED_CARDIOMYOPATHY<br>• KEGG_TOLL_LIKE_RECEPTOR_SIGNALING_PATHWAY<br>• KEGG_REGULATION_OF_ACTIN_CYTOSKELETON<br>• KEGG_HYPERTROPHIC_CARDIOMYOPATHY_HCM<br>• KEGG_WNT_SIGNALING_PATHWAY<br>• KEGG_NATURAL_KILLER_CELL_MEDIATED_CYTOTOXICITY<br>• KEGG_INTESTINAL_IMMUNE_NETWORK_FOR_IGA_PRODUCTION<br>• KEGG_HEMATOPOIETIC_CELL_LINEAGE<br>• KEGG_ECM_RECEPTOR_INTERACTION<br>• KEGG_ADHERENS_JUNCTION
5	• KEGG_GRAFT_VERSUS_HOST_DISEASE<br>• KEGG_NOD_LIKE_RECEPTOR_SIGNALING_PATHWAY<br>• KEGG_TYPE_I_DIABETES_MELLITUS<br>• KEGG_B_CELL_RECEPTOR_SIGNALING_PATHWAY<br>• KEGG_HYPERTROPHIC_CARDIOMYOPATHY_HCM<br>• KEGG_CELL_ADHESION_MOLECULES_CAMS<br>• KEGG_INTESTINAL_IMMUNE_NETWORK_FOR_IGA_PRODUCTION<br>• KEGG_HEMATOPOIETIC_CELL_LINEAGE<br>• KEGG_LEUKOCYTE_TRANSENDOTHELIAL_MIGRATION<br>• KEGG_TOLL_LIKE_RECEPTOR_SIGNALING_PATHWAY<br>• KEGG_CELL_ADHESION_MOLECULES_CAMS<br>• KEGG_NATURAL_KILLER_CELL_MEDIATED_CYTOTOXICITY<br>• KEGG_ADHERENS_JUNCTION
8	• KEGG_NOD_LIKE_RECEPTOR_SIGNALING_PATHWAY<br>• KEGG_DILATED_CARDIOMYOPATHY<br>• KEGG_PRIMARY_IMMUNODEFICIENCY<br>• KEGG_TOLL_LIKE_RECEPTOR_SIGNALING_PATHWAY<br>• KEGG_HYPERTROPHIC_CARDIOMYOPATHY_HCM<br>• KEGG_HEMATOPOIETIC_CELL_LINEAGE<br>• KEGG_GRAFT_VERSUS_HOST_DISEASE<br>• KEGG_AUTOIMMUNE_THYROID_DISEASE<br>• KEGG_PRIMARY_IMMUNODEFICIENCY<br>• KEGG_TOLL_LIKE_RECEPTOR_SIGNALING_PATHWAY<br>• KEGG_B_CELL_RECEPTOR_SIGNALING_PATHWAY<br>• KEGG_HYPERTROPHIC_CARDIOMYOPATHY_HCM<br>• KEGG_ADHERENS_JUNCTION

K-Hop	Identified KEGG Pathways
11	• KEGG_DILATED_CARDIOMYOPATHY<br>• KEGG_TOLL_LIKE_RECEPTOR_SIGNALING_PATHWAY<br>• KEGG_HEDGEHOG_SIGNALING_PATHWAY<br>• KEGG_HYPERTROPHIC_CARDIOMYOPATHY_HCM<br>• KEGG_WNT_SIGNALING_PATHWAY<br>• KEGG_HEMATOPOIETIC_CELL_LINEAGE<br>• KEGG_RIG_I_LIKE_RECEPTOR_SIGNALING_PATHWAY<br>• KEGG_DILATED_CARDIOMYOPATHY<br>• KEGG_PRIMARY_IMMUNODEFICIENCY<br>• KEGG_TOLL_LIKE_RECEPTOR_SIGNALING_PATHWAY<br>• KEGG_HYPERTROPHIC_CARDIOMYOPATHY_HCM<br>• KEGG_CELL_ADHESION_MOLECULES_CAMS<br>• KEGG_TGF_BETA_SIGNALING_PATHWAY<br>• KEGG_HEMATOPOIETIC_CELL_LINEAGE<br>• KEGG_ADHERENS_JUNCTION
sc	• KEGG_CHEMOKINE_SIGNALING_PATHWAY<br>• KEGG_ECM_RECEPTOR_INTERACTION<br>• KEGG_JAK_STAT_SIGNALING_PATHWAY<br>• KEGG_GRAFT_VERSUS_HOST_DISEASE<br>• KEGG_AUTOIMMUNE_THYROID_DISEASE<br>• KEGG_BLADDER_CANCER<br>• KEGG_TYPE_I_DIABETES_MELLITUS<br>• KEGG_GLIOMA<br>• KEGG_JAK_STAT_SIGNALING_PATHWAY<br>• KEGG_ECM_RECEPTOR_INTERACTION

Table S20: Identified KEGG Pathways per niche sizes of the merged GAT I2C prediction heads (predicted counts resulted in: 11 clusters for 1-hop; 12 for 2-hop; 11 for 3-hop; 10 for 5-hop; 13 for 8-hop; 12 for 11-hop; 20 for single cells, true counts in: 9 clusters for 1-hop; 11 for 2-hop; 13 for 3-hop; 20 for 5-hop; 28 for 8-hop; 36 for 11-hop; 14 for single cells).

Compartment	Enriched Cell Types
Cancer / Epithelial	• Cancer cell<br>• Cancer stem cell<br>• Basal cell
Stromal	• Fibroblast<br>• Stromal cell<br>• Myofibroblast
Immune	• T cell<br>• CD8+ T cell<br>• Macrophage<br>• Monocyte<br>• Dendritic cell

Table S21: Top 5 enriched cell type populations correctly identified in CosMx single cells from merged GAT Image2Count (predicted counts resulted in 13 clusters, true counts in 19).

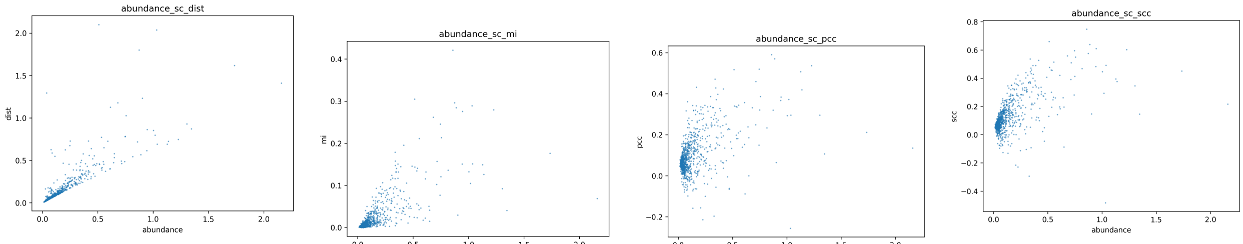

a Sc marker abundance to log1p MSE

b Sc marker abundance to MI

c Sc marker abundance to PCC

d Sc marker abundance to log1p SCC

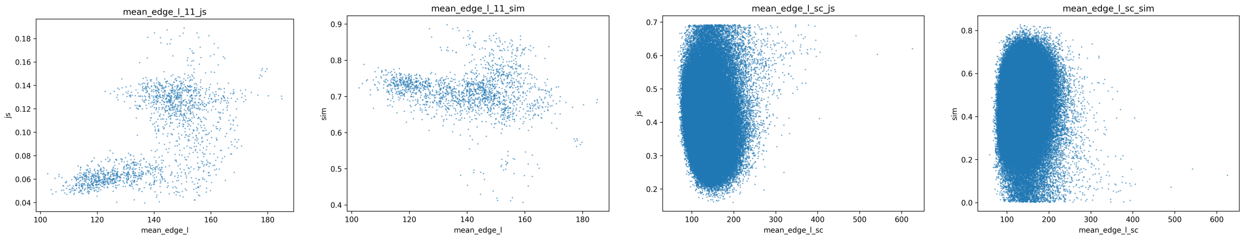

e 11-hop subgraph mean edge length to js_div

f 11-hop subgraph mean edge length to CosSim

g Sc mean edge length to js_div

h Sc mean edge length to CosSim

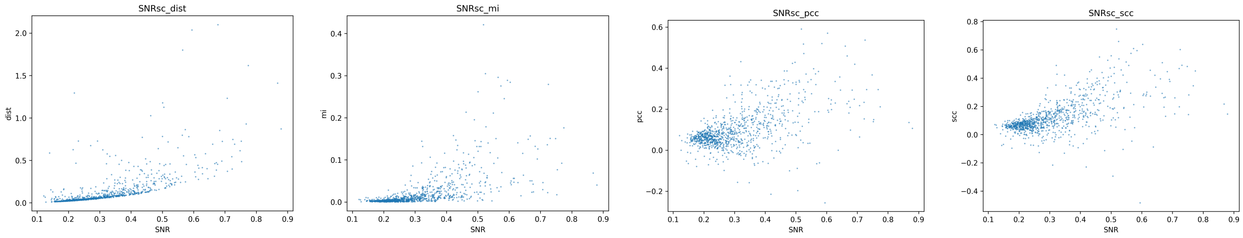

i Sc marker SNR to log1p MSE

j Sc marker SNR to MI

k Sc marker SNR to PCC

l Sc marker SNR to SCC

Figure S1: Predicted CosMx single cell protein expression performance of merged GAT I2C prediction heads in relation to marker abundance (Fig. S1 **a**-**d**, mean edge length (Fig S1 **e**-**h** and SNR (Fig. 1 **i**-**l**.

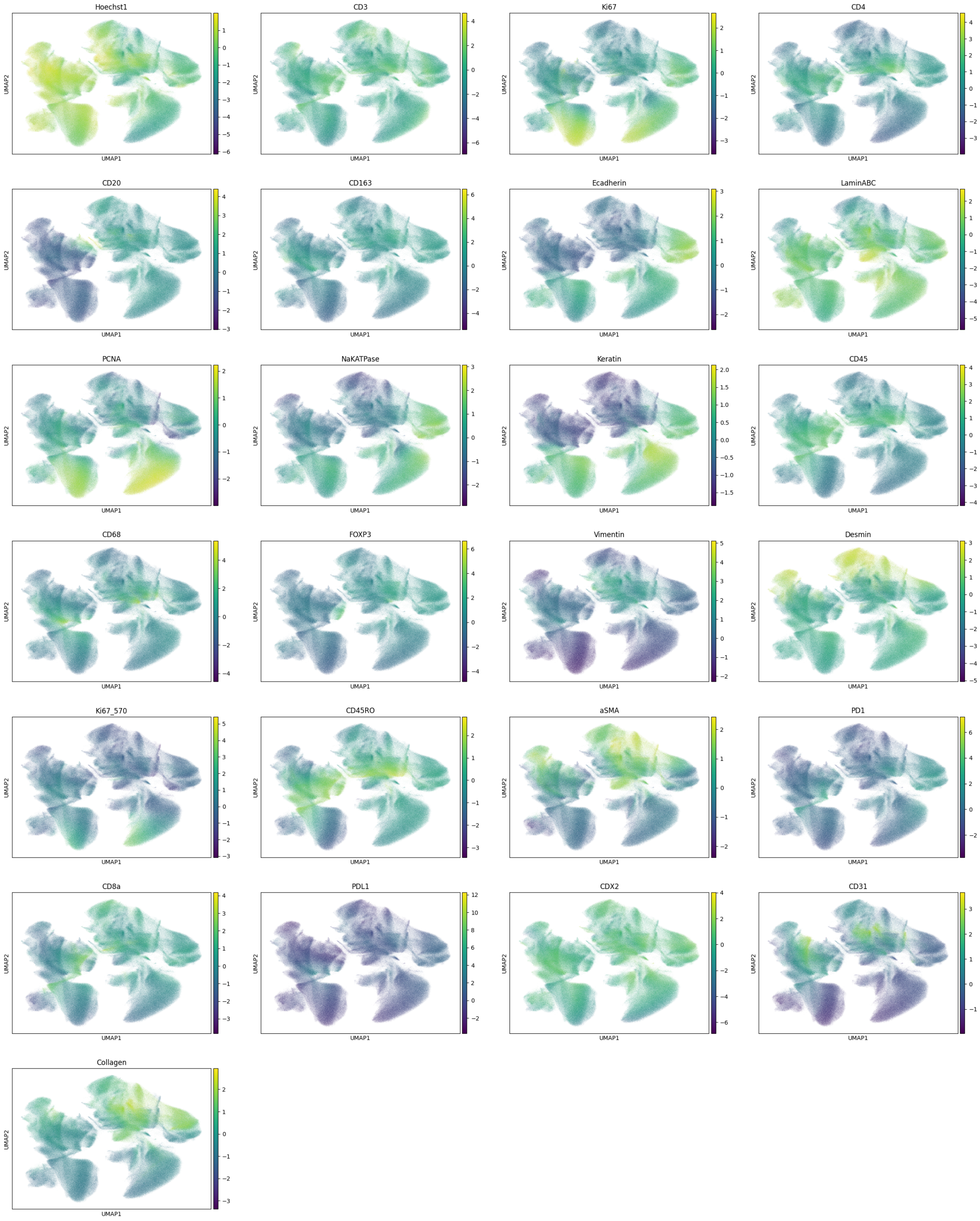

Figure S2: Normalized expression of cells in UMAP for all markers in the original t-CycIF CRC test data.

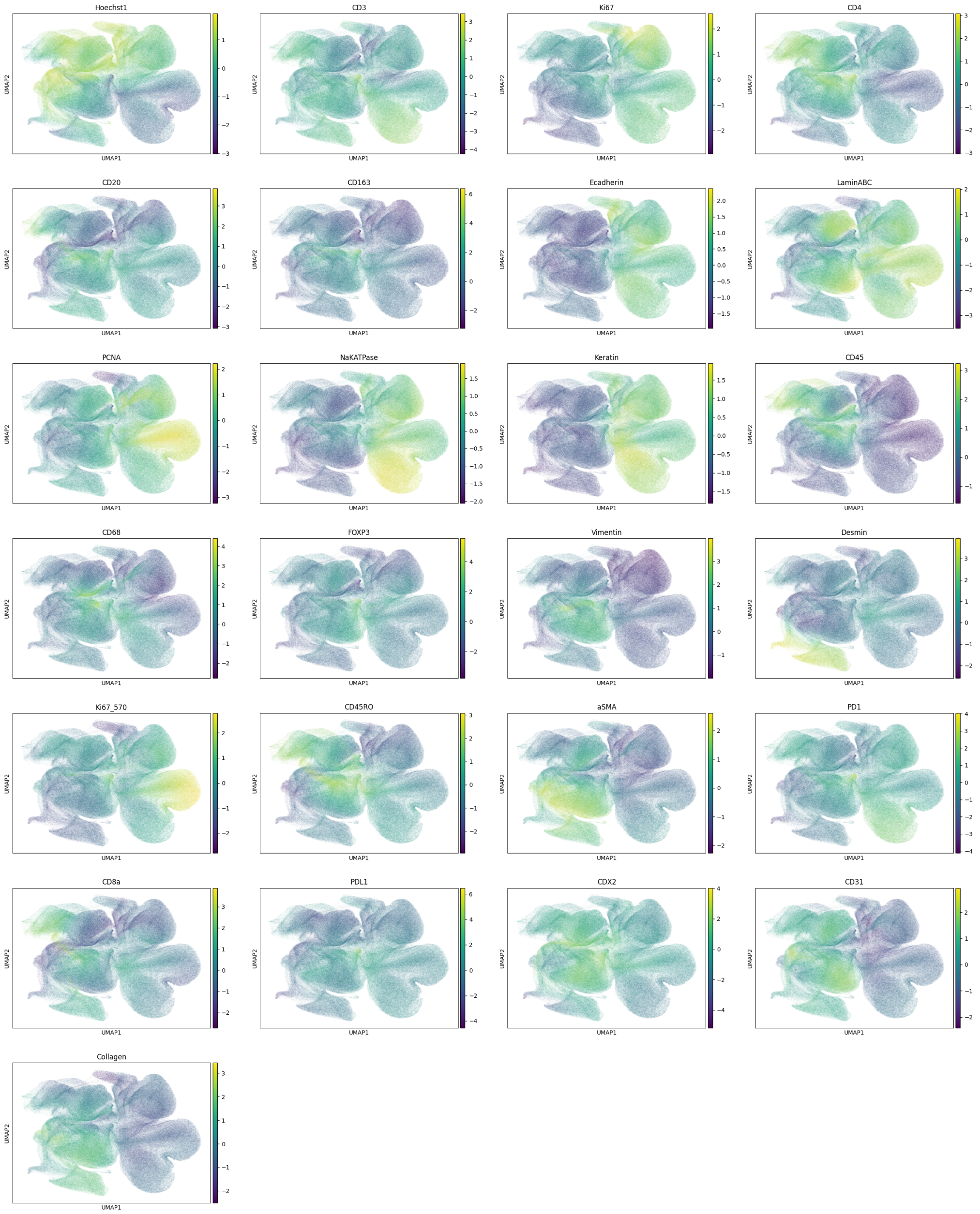

Figure S3: Normalized expression of cells in UMAP for t-CycIF CRC GAT Image2Count model predictions on test data.

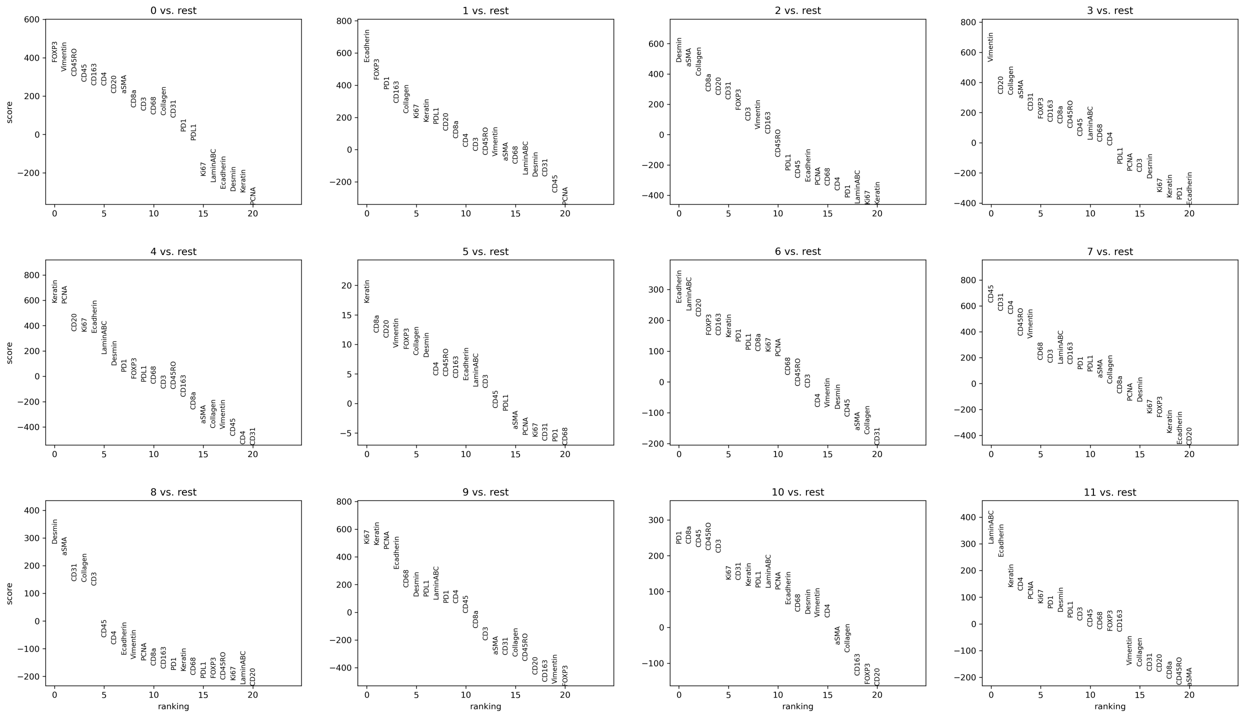

Figure S4: Wilcoxon rank-sum test associated proteins and leiden clusters for t-CycIF CRC test data.

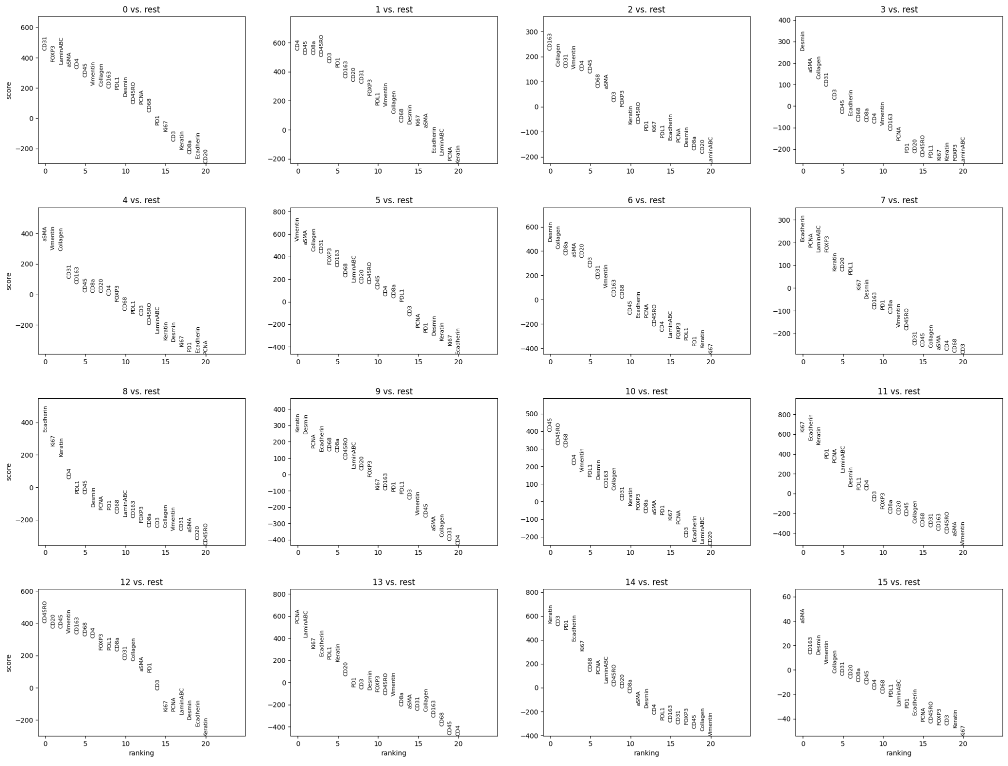

Figure S5: Wilcoxon rank-sum test associated proteins and Leiden clusters with resolution of 0.25 for t-CycIF CRC GAT Image2Count model predictions on test data.

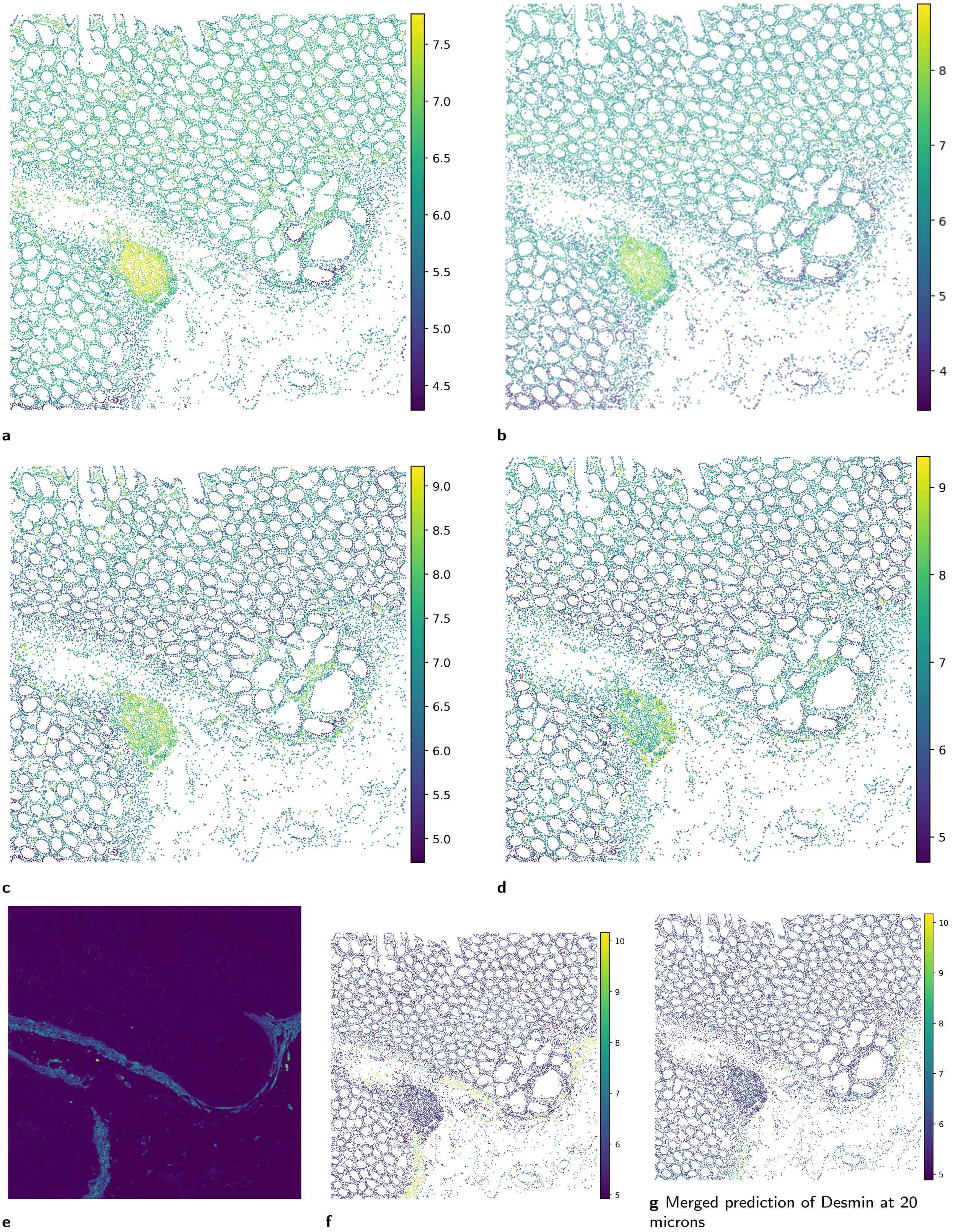

g Merged prediction of Desmin at 20 microns

Figure S6: Predicted CRC single cell protein expression of different GAT Image2Count models. **a** Merged prediction of CD20 at 50 microns; **b** Non merged prediction of CD20 at 50 microns; **c** Merged prediction of CD8a at 50 microns; **d** Merged prediction of CD8a at 20 microns; **e** Desmin channel; **f** Merged prediction of Desmin at 50 microns; **g** Merged prediction of Desmin at 50 microns

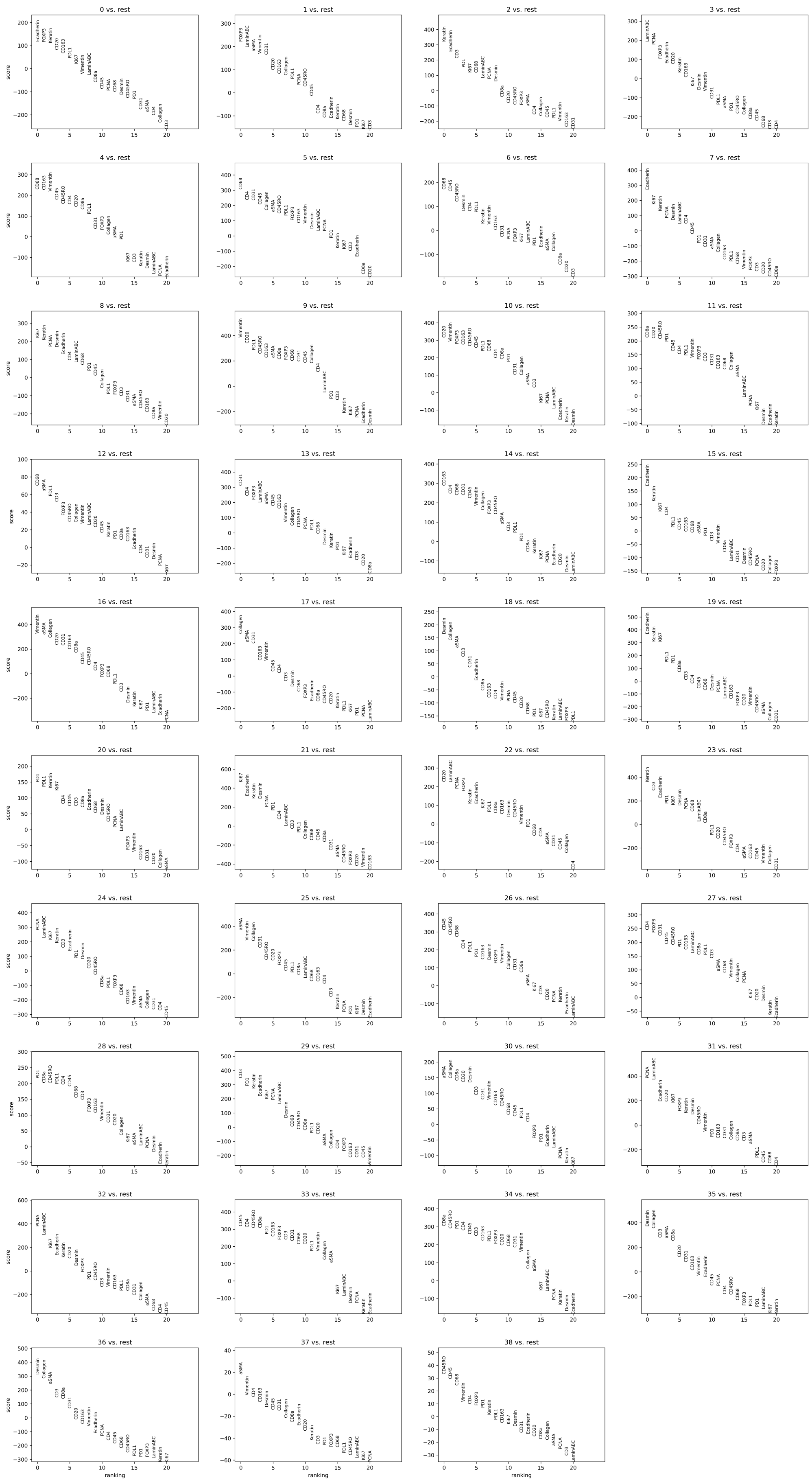

Figure S7: Wilcoxon rank-sum test associated proteins and Leiden clusters with resolution of 1 for t-CycIF CRC GAT Image2Count model predictions on test data.

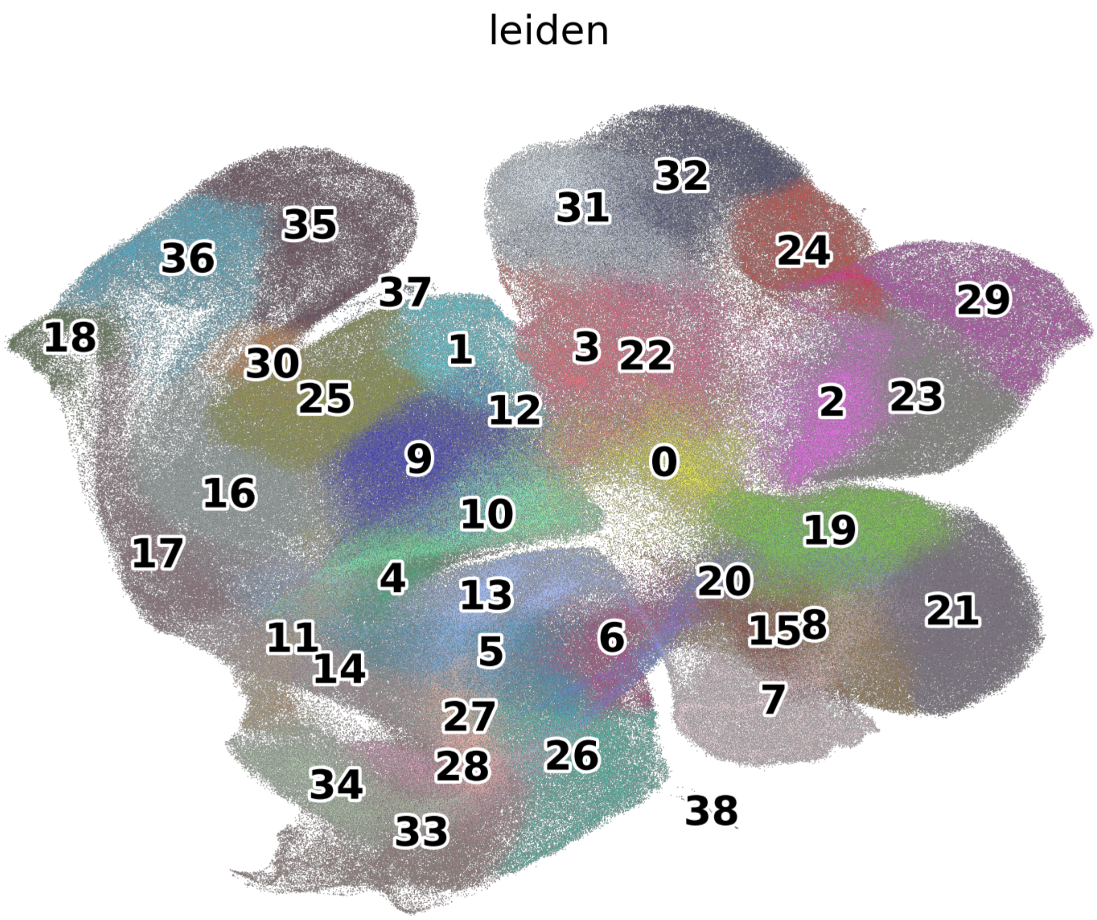

Figure S8: UMAP and leiden clusters with resolution of 1 for t-CycIF CRC GAT Image2Count model predictions on test data.

markers	mi	dist	pcc	scc	kcc
Hoechst1	0.772714	0.124538	0.834222	0.880646	0.682919
CD3	0.036124	1.054042	0.203365	0.200021	0.132351
Ki67	0.388706	1.385858	0.529505	0.719106	0.524245
CD4	0.301045	0.581185	0.520823	0.611157	0.429786
CD20	0.214673	0.772867	0.280326	0.512837	0.344814
CD163	0.122898	0.431151	0.241031	0.416420	0.287559
Ecadherin	0.553282	0.590281	0.581625	0.755958	0.524145
LaminABC	0.105571	0.784915	0.229382	0.329015	0.222091
PCNA	0.201743	2.552219	0.337412	0.433842	0.314398
NaKATPase	0.496663	0.693865	0.576530	0.772981	0.566022
Keratin	1.011716	0.457549	0.899013	0.913004	0.746583
CD45	0.609214	0.453817	0.744611	0.794482	0.602916
CD68	0.090072	1.077224	0.251212	0.332729	0.226267
FOXP3	0.089020	0.557674	0.133080	0.305717	0.211697
Vimentin	0.379222	0.708028	0.449622	0.728638	0.528180
Desmin	0.231686	1.324365	0.530419	0.377592	0.264712
Ki67_570	0.128574	1.487465	0.216644	0.265753	0.190480
CD45RO	0.373589	0.742431	0.657088	0.674597	0.491031
aSMA	0.279776	1.831156	0.475560	0.584308	0.402291
PD1	0.175438	0.379382	0.346094	0.456997	0.316275
CD8a	0.409350	0.437547	0.709511	0.693922	0.513137
PDL1	0.042868	0.150372	0.107027	0.166675	0.114510
CDX2	0.050711	0.432485	0.195532	0.264334	0.178527
CD31	0.335159	1.815371	0.323995	0.659224	0.458044
Collagen	0.173304	2.326235	0.202811	0.494079	0.343250

Table S22: Comparison of KCC, SCC, PCC, MSE of log1p counts and MI Metrics across Markers for single-cell data of CRC GAT Image2Count merged model predictions on test data. FDR corrected P-values are well below 0.00001.

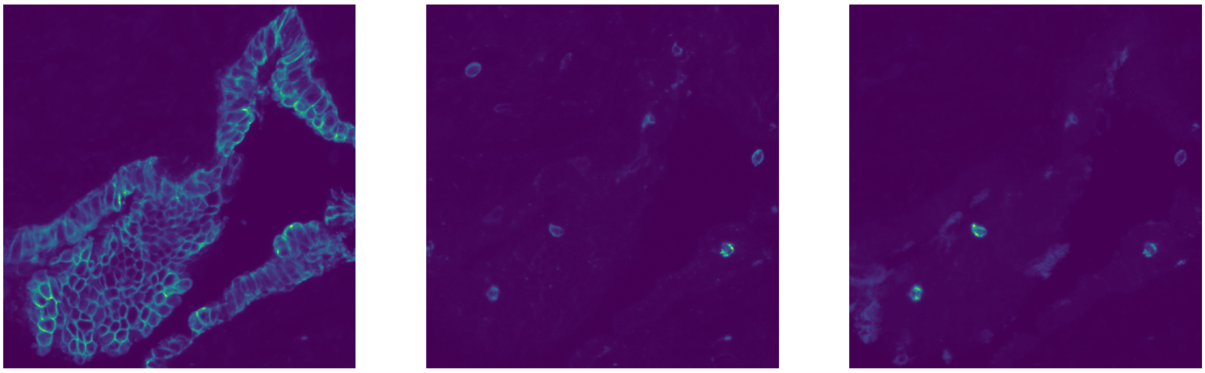

a b c

Figure S9: PanCk **a**, CD45 **b** and CD8 **c** image channels of GeoMx ROI 019-1C54.

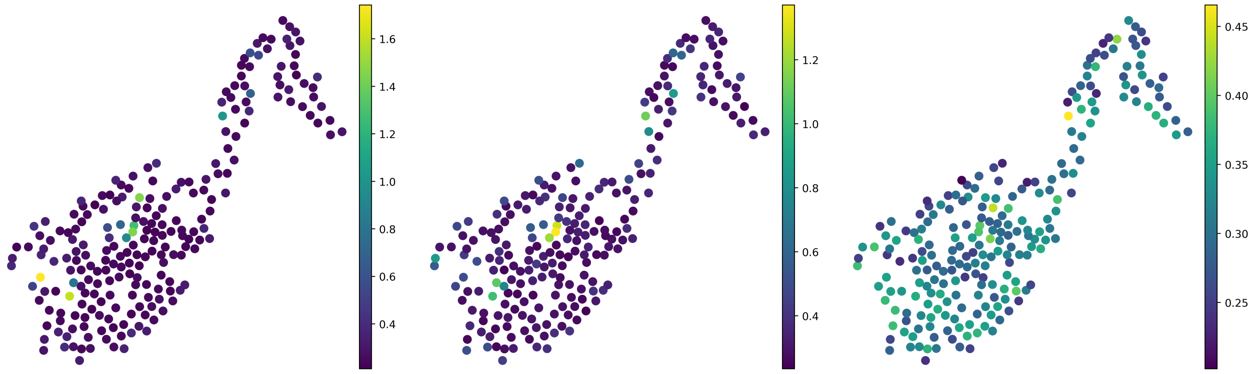

a b c

Figure S10: Normalized merged FFW Image2Count model predictions of CD8 at 8 (**a**), 14 (**b**) and 20 (**c**) microns of a representative test set image including GeoMx ROI 019-1C54.

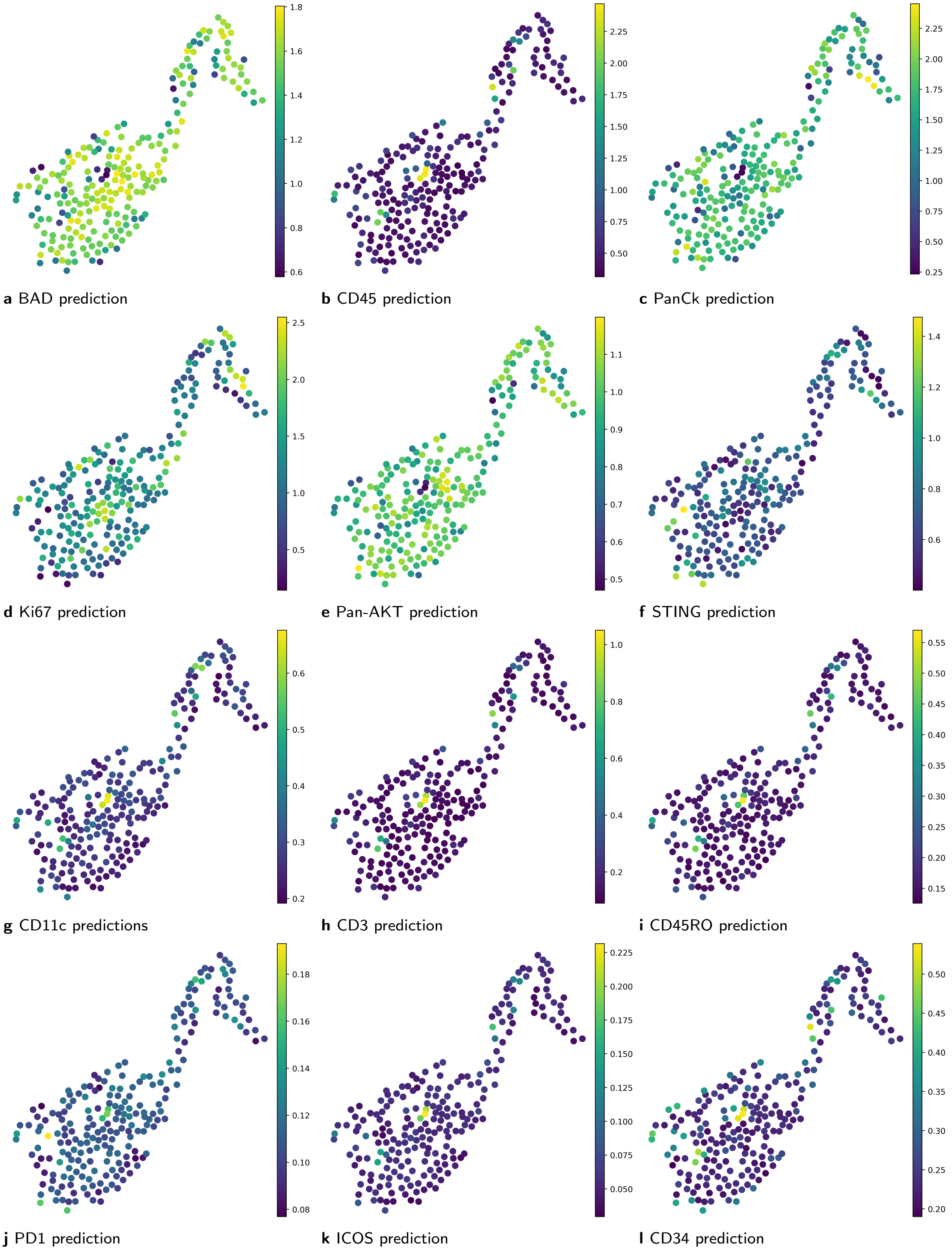

Figure S11: Normalized merged FFW Image2Count predictions of 12 microns cell cut-outs and image channels of a representative test set image, GeoMx ROI 019-1C54.

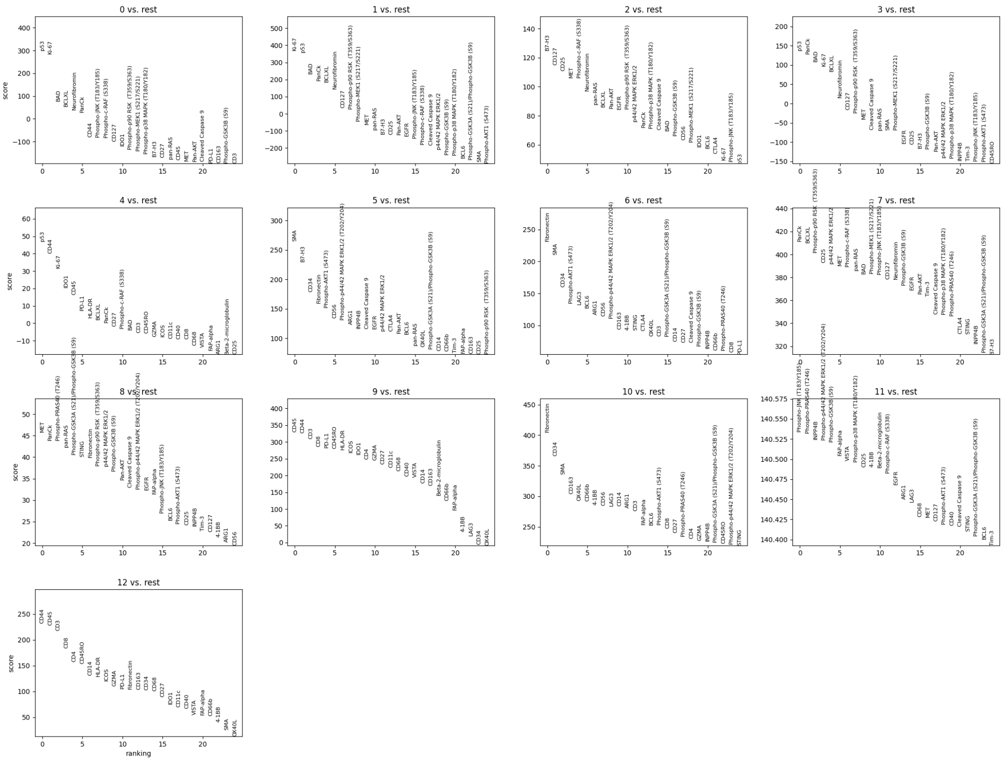

Figure S12: Wilcoxon rank-sum test associated proteins and Leiden clusters with resolution of 0.25 for GeoMx OC merged FFW Image2Count prediction head model predictions on test data.

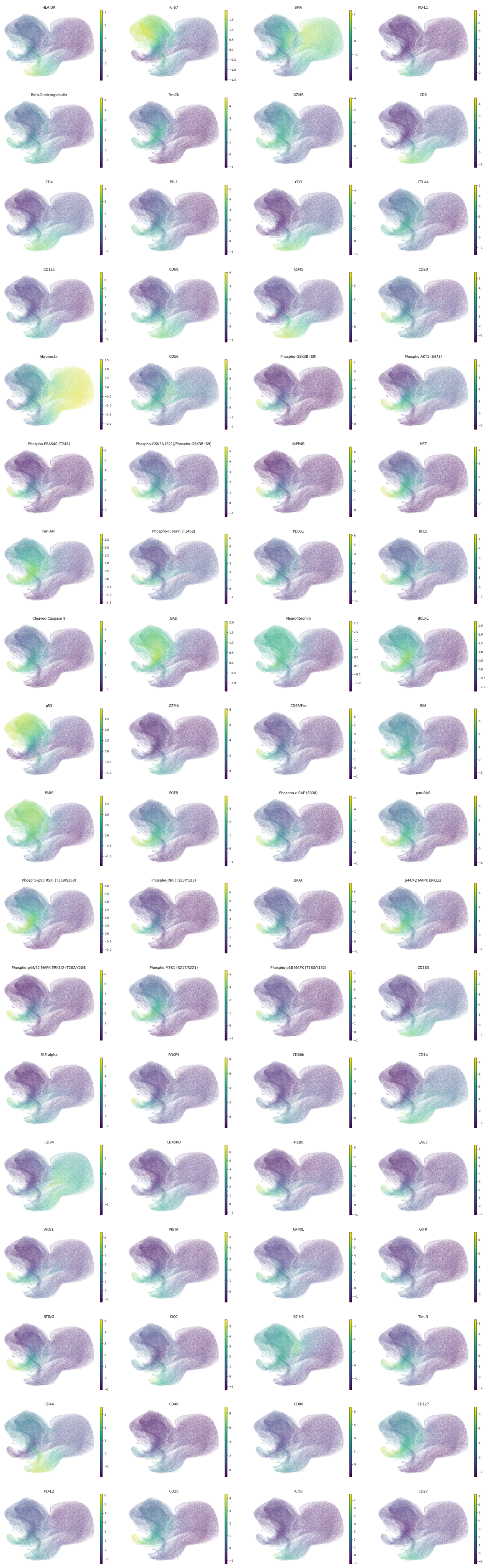

Figure S13: Normalized expression of cells in UMAP for GeoMx OC FFW Image2Count model predictions on test data.

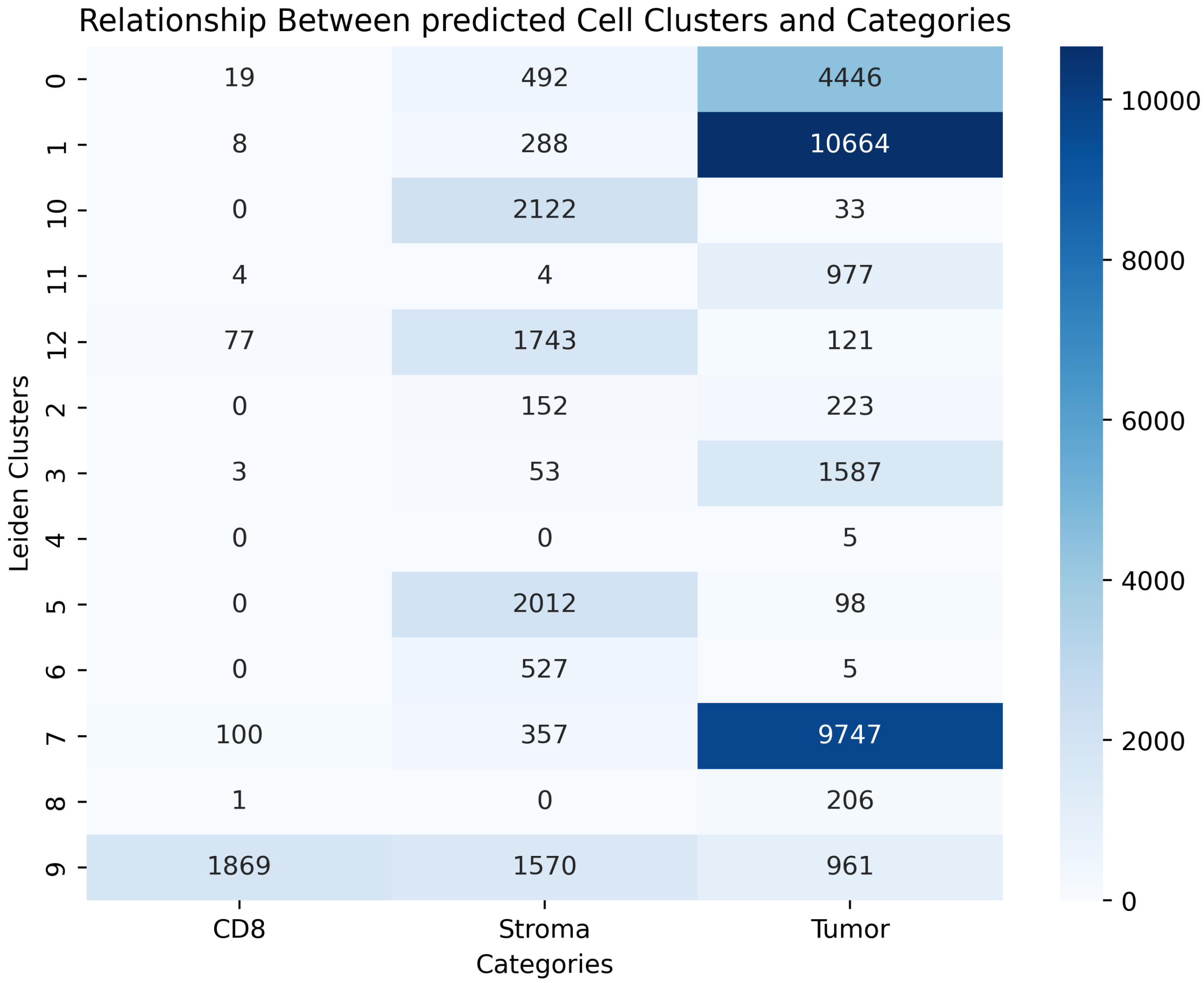

Figure S14: Leiden Clusters annotated in Figure S12 of GeoMx OC merged FFW Image2Count prediction head predictions and corresponding QuPath annotation of cells in GeoMx test ROIs, with annotation categories CD8, Tumor and Stroma based on IF staining, with number of cells per Leiden cluster/QuPath annotation.

Immune Cell Profiling Panel *Human Protein Core*						IO Drug Target Panel *Human Protein Module*	
Beta-2-microglobulin	CD3	CD56	CTLA4	GZMB	PD-1	4-1BB	LAG3
CD11c	CD4	CD68	Pan-cytokeratin	HLA-DR	PD-L1	ARG1	OX40L
CD20	CD45	CD8	Fibronectin	Ki-67	SMA	B7-H3	STING
Ms IgG2a			Histone H3			GITR	TIM-3
Ms IgG1			S6			IDO1	VISTA
Rb IgG			GAPDH				

Immune Activation Status Panel *Human Protein Module*		Cell Death Panel *Human Protein Module*		PI3K/AKT Signaling Panel *Human Protein Module*	
CD127	CD80	BAD	GZMA	Pan-AKT	Phospho-GSK3A (S21)/Phospho-GSK3B (S9)
CD25	ICOS	BCL6	p53	MET	INPP4B
CD27	PD-L2	BCLXL	PARP	Phospho-AKT1 (S473)	PLCG1
CD40		BIM	Cleaved Caspase 9	Phospho-GSK3B (S9)	Phospho-PRAS40 (T246)
CD44		CD95/Fas	Neurofibromin	Phospho-Tuberin (T1462)	

Immune Cell Typing Panel *Human Protein Module*		MAPK Signaling Panel *Human Protein Module*	
CD14	FAPalpha	EGFR	Phospho-MEK1 (S217/S221)
CD163	FOXP3	pan-RAS	Phospho-p38 MAPK (T180/Y182)
CD34		BRAF	Phospho-p44/42 MAPK ERK1/2 (T202/Y204)
CD45RO		Phospho-c-RAF (S338)	p44/42 MAPK ERK1/2
CD66b		Phospho-JNK (T183/Y185)	Phospho-p90 RSK (T359/S363)

Figure S15: Antibody specificities used for GeoMx ROIs.

markers	SCC	SCC_pval	PCC	PCC_pval	MI
mean	0.276567	0.038768	0.246218	0.093973	0.058624
std	0.155608	0.114339	0.175770	0.179168	0.060719
CD45	0.657450	0.000000	0.630704	0.000000	0.220734
PanCk	0.580856	0.000000	0.655010	0.000000	0.178077
CD3	0.554316	0.000000	0.559815	0.000000	0.096299
CD8	0.544065	0.000000	0.486475	0.000000	0.130738
CD14	0.533209	0.000000	0.350711	0.000052	0.146621
CD34	0.532801	0.000000	0.564761	0.000000	0.146495
CD44	0.503338	0.000000	0.175425	0.051430	0.000000
Fibronectin	0.490096	0.000000	0.773253	0.000000	0.231207
CD4	0.473097	0.000000	0.434057	0.000000	0.124497
CD11c	0.449906	0.000000	0.380260	0.000010	0.108956
CD163	0.434485	0.000000	0.283795	0.001154	0.065474
VISTA	0.428323	0.000000	0.253883	0.003868	0.098874
CD45RO	0.406078	0.000001	0.411954	0.000001	0.133221
CD68	0.400272	0.000002	0.282645	0.001177	0.105782
ICOS	0.382216	0.000007	0.348577	0.000056	0.098100
ARG1	0.376698	0.000009	0.343249	0.000072	0.060995
Phospho-AKT1 (S473)	0.370440	0.000013	0.357417	0.000039	0.014162
IDO1	0.367325	0.000015	0.396273	0.000003	0.065658
SMA	0.351694	0.000039	0.596297	0.000000	0.040138
FAP-alpha	0.345941	0.000052	0.217984	0.013603	0.058449
STING	0.342876	0.000060	0.351687	0.000052	0.036995
BAD	0.342076	0.000060	0.322040	0.000183	0.018846
pan-RAS	0.339103	0.000068	0.042616	0.621866	0.205840
Phospho-p90 RSK (T359/S363)	0.317580	0.000225	0.336569	0.000096	0.068911
GZMA	0.313562	0.000269	0.101309	0.244533	0.040735
HLA-DR	0.307528	0.000354	0.103042	0.239494	0.082739
BCLXL	0.306991	0.000354	0.286378	0.001056	0.000000
Pan-AKT	0.304499	0.000390	0.233777	0.007758	0.029136
CD27	0.298372	0.000500	0.314346	0.000268	0.024123
INPP4B	0.289814	0.000726	0.521502	0.000000	0.157539
Beta-2-microglobulin	0.289750	0.000726	0.175055	0.051430	0.000000
MET	0.288306	0.000756	0.336534	0.000096	0.092410
Phospho-MEK1 (S217/S221)	0.283673	0.000922	0.231739	0.008195	0.000000
CD40	0.274425	0.001395	0.172605	0.052008	0.048262
CD25	0.270080	0.001661	0.151338	0.091067	0.044388
4-1BB	0.268668	0.001725	0.185960	0.038920	0.116501
Phospho-PRAS40 (T246)	0.261283	0.002349	0.326183	0.000162	0.048759
p53	0.255010	0.003021	0.163084	0.067811	0.018060
Tim-3	0.250164	0.003633	0.176191	0.051430	0.000000
CD56	0.249481	0.003650	0.016205	0.855849	0.083024
Cleaved Caspase 9	0.246804	0.003976	0.241494	0.005819	0.000000
Phospho-p38 MAPK (T180/Y182)	0.246349	0.003976	0.267335	0.002272	0.013279
CD127	0.245040	0.004106	0.245595	0.005129	0.000000
LAG3	0.236204	0.005783	0.129655	0.143747	0.000000
BCL6	0.231408	0.006860	0.322614	0.000183	0.001632
Ki-67	0.222060	0.009670	0.130200	0.143747	0.069317
Phospho-p44/42 MAPK ERK1/2 (T202/Y204)	0.220349	0.010107	0.067445	0.430104	0.000000
B7-H3	0.219802	0.010109	0.246361	0.005129	0.000000
p44/42 MAPK ERK1/2	0.218931	0.010238	0.120281	0.168328	0.113733
Phospho-GSK3A (S21)/Phospho-GSK3B (S9)	0.216947	0.010816	0.245192	0.005129	0.022928
Phospho-JNK (T183/Y185)	0.211410	0.013021	0.325800	0.000162	0.014956
Phospho-c-RAF (S338)	0.206411	0.015310	0.146808	0.099196	0.001912
Phospho-GSK3B (S9)	0.204992	0.015808	0.186689	0.038920	0.000000
PARP	0.192590	0.023849	0.205090	0.021266	0.000000
EGFR	0.191765	0.024082	0.172967	0.052008	0.100196
CD66b	0.189777	0.025285	0.293155	0.000783	0.000000
PD-L1	0.179661	0.034526	0.095628	0.262635	0.000315
OX40L	0.170384	0.045283	0.077843	0.363941	0.017275
BRAF	0.163934	0.054012	0.262409	0.002737	0.143236

Continued on next page...

Table – continued from previous page

markers	SCC	SCC_pval	PCC	PCC_pval	MI
GZMB	0.160204	0.059239	0.172629	0.052008	0.000000
Neurofibromin	0.156258	0.065263	0.156158	0.081156	0.000000
PLCG1	0.148428	0.079859	0.121610	0.165860	0.071339
Phospho-Tuberin (T1462)	0.135549	0.110420	0.108023	0.218604	0.082419
PD-1	0.127382	0.131909	0.125455	0.155599	0.019447
PD-L2	0.125142	0.136529	0.139007	0.118417	0.107147
CD20	0.123147	0.141108	0.124992	0.155599	0.000000
BIM	0.122269	0.141996	0.150274	0.091979	0.000000
CD95/Fas	0.104618	0.208423	0.096342	0.262635	0.000000
GITR	0.071301	0.391348	0.099602	0.249507	0.054601
FOXP3	0.016305	0.843019	0.006296	0.939052	0.017709
CD80	-0.127183	0.131909	-0.138723	0.118417	0.000000
CTLA4	-0.301260	0.000446	-0.267240	0.002272	0.128724

Table S23: Comparison of SCC, PCC, and MI metrics across markers for ROI data of GeoMx OC FFW Image2Count merged model predictions on test data. P-values are FDR corrected.

metric	6_6-17_0	lin-17_0	lin-6_6
mi_1_coef	0.002839	-0.005387	-0.008227
mi_1_pv	0.605093	0.287725	0.081620
dist_1_coef	-0.015620	-0.016985	-0.001365
dist_1_pv	0.630838	0.611505	0.955784
pcc_1_coef	0.001780	-0.052733	-0.054513
pcc_1_pv	0.837790	0.000000	0.000000
scc_1_coef	0.000809	-0.069232	-0.070040
scc_1_pv	0.931786	0.000000	0.000000
mi_2_coef	0.003530	-0.012992	-0.016521
mi_2_pv	0.614979	0.028642	0.003842
dist_2_coef	-0.021041	-0.035642	-0.014602
dist_2_pv	0.621072	0.380659	0.730756
pcc_2_coef	0.003820	-0.076778	-0.080599
pcc_2_pv	0.729172	0.000000	0.000000
scc_2_coef	0.004917	-0.094098	-0.099015
scc_2_pv	0.673829	0.000000	0.000000
mi_3_coef	0.007085	-0.017345	-0.024430
mi_3_pv	0.335397	0.006036	0.000059
dist_3_coef	-0.021753	-0.052876	-0.031123
dist_3_pv	0.647252	0.239814	0.506519
pcc_3_coef	0.006985	-0.102245	-0.109230
pcc_3_pv	0.566668	0.000000	0.000000
scc_3_coef	0.009135	-0.120244	-0.129379
scc_3_pv	0.454069	0.000000	0.000000
mi_5_coef	0.012146	-0.027460	-0.039606
mi_5_pv	0.087334	0.000014	0.000000
dist_5_coef	-0.019439	-0.067640	-0.048201
dist_5_pv	0.714475	0.159140	0.335397
pcc_5_coef	0.010336	-0.136622	-0.146958
pcc_5_pv	0.415605	0.000000	0.000000
scc_5_coef	0.015130	-0.158644	-0.173774
scc_5_pv	0.239814	0.000000	0.000000
mi_8_coef	0.019470	-0.030921	-0.050390
mi_8_pv	0.000981	0.000000	0.000000
dist_8_coef	-0.016439	-0.067754	-0.051316
dist_8_pv	0.764644	0.176710	0.325313
pcc_8_coef	0.012116	-0.142823	-0.154939
pcc_8_pv	0.375722	0.000000	0.000000
scc_8_coef	0.016909	-0.161636	-0.178545
scc_8_pv	0.210059	0.000000	0.000000
mi_11_coef	0.020063	-0.041873	-0.061937

Continued on next page...

Table – continued from previous page

metric	6_6-17_0	lin-17_0	lin-6_6
mi_11_pv	0.000632	0.000000	0.000000
dist_11_coef	-0.015007	-0.066080	-0.051072
dist_11_pv	0.782984	0.193317	0.331910
pcc_11_coef	0.012204	-0.139759	-0.151962
pcc_11_pv	0.380659	0.000000	0.000000
scc_11_coef	0.015732	-0.151032	-0.166763
scc_11_pv	0.239814	0.000000	0.000000
mi_sc_coef	0.000516	-0.004191	-0.004707
mi_sc_pv	0.828473	0.035612	0.015967
dist_sc_coef	-0.002398	-0.003362	-0.000963
dist_sc_pv	0.837763	0.775437	0.931786
pcc_sc_coef	0.003863	-0.026188	-0.030051
pcc_sc_pv	0.490577	0.000000	0.000000
scc_sc_coef	0.001244	-0.041803	-0.043047
scc_sc_pv	0.847185	0.000000	0.000000
sim_sc_coef	0.002621	0.015308	0.012687
sim_sc_pv	0.000000	0.000000	0.000000
js_div_sc_coef	0.002511	0.002586	0.000076
js_div_sc_pv	0.000000	0.000000	0.790255
sim_1_coef	0.001578	0.018241	0.016663
sim_1_pv	0.664072	0.000000	0.000000
js_div_1_coef	0.001677	0.000365	-0.001312
js_div_1_pv	0.406486	0.847185	0.538311
sim_2_coef	0.001119	0.018120	0.017001
sim_2_pv	0.714475	0.000000	0.000000
js_div_2_coef	0.001121	-0.000072	-0.001193
js_div_2_pv	0.387946	0.952434	0.367063
sim_3_coef	0.001440	0.018307	0.016867
sim_3_pv	0.614979	0.000000	0.000000
js_div_3_coef	0.000983	-0.000216	-0.001199
js_div_3_pv	0.348767	0.837790	0.239814
sim_5_coef	0.001797	0.018788	0.016991
sim_5_pv	0.380659	0.000000	0.000000
js_div_5_coef	0.000840	-0.000418	-0.001257
js_div_5_pv	0.239814	0.592372	0.056813
sim_8_coef	0.002033	0.019339	0.017306
sim_8_pv	0.230607	0.000000	0.000000
js_div_8_coef	0.000748	-0.000530	-0.001278
js_div_8_pv	0.185595	0.364701	0.011204
sim_11_coef	0.002023	0.019460	0.017437
sim_11_pv	0.149386	0.000000	0.000000
js_div_11_coef	0.000689	-0.000574	-0.001264
js_div_11_pv	0.151808	0.239814	0.002996

Table S24: Comparison of SCC, PCC, and MI metrics performance across markers for ROI data of CosMx GAT Image2Count merged model (6_6), FFW Image2Count merged model (17_0) and ResNet model (lin) predictions on test data at single-cell and different k-hop resolution. P-values are FDR corrected.

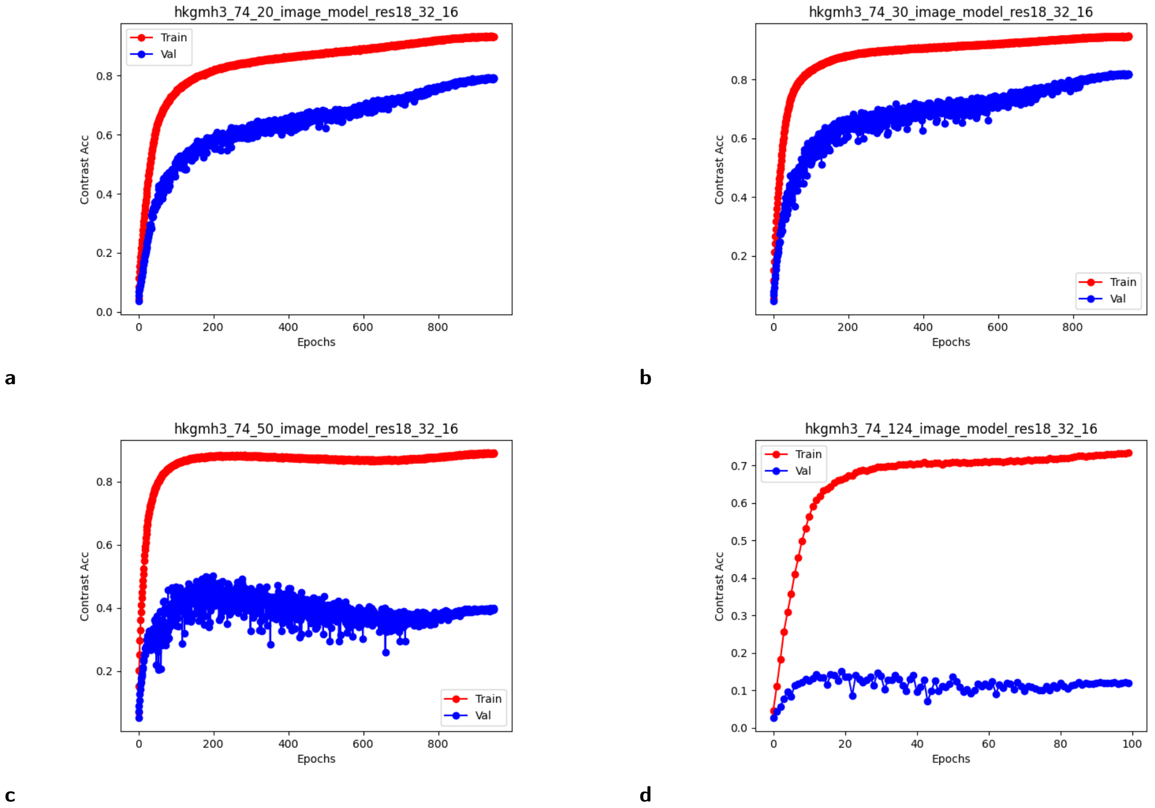

Figure S16: SimCLR contrast accuracy of visual feature encoders trained on the GeoMx dataset. **a** ResNet18 trained on GeoMx training data with cell cut-outs of size 8×8 µm (20×20 pixels); **b** ResNet18 trained on GeoMx training data with cell cut-outs of size 12×12 µm (30×30 pixels); **c** ResNet18 trained on GeoMx training data with cell cut-outs of size 20×20 µm (50×50 pixels); **d** ResNet18 trained on GeoMx training data with cell cut-outs of size 50×50 µm (124×124 pixels)
